# Supplementary material for: The experience of loneliness among people with psychosis: Qualitative meta-synthesis
Source: PLoS One. 2024 Dec 31;19(12):e0315763. doi: 10.1371/journal.pone.0315763 (PMC11687762; doi:10.1371/journal.pone.0315763)
Supplement: S2 Appendix — (DOCX) [file pone.0315763.s002.docx]

**Appendix S2: ENTREQ Checklist**

Table. Enhancing transparency in reporting the synthesis of qualitative research: ENTREQ Checklist (Tong, et al., 2012)

| Item No. | Guide and Description | Report Location |
| --- | --- | --- |
| 1. Aim | State the research question the synthesis addresses | Introduction |
| 2. Synthesis methodology | Identify the synthesis methodology or theoretical framework which underpins the synthesis, and describe the rationale for choice of methodology (e.g. meta-ethnography, thematic synthesis, critical interpretive synthesis, grounded theory synthesis, realist synthesis, meta-aggregation, meta-study, framework synthesis) | Methods – *Design* and *Data analysis* |
| 3. Approach to searching | Indicate whether the search was pre-planned (comprehensive search strategies to seek all available studies) or iterative (to seek all available concepts until they theoretical saturation is achieved) | Methods – *Database and search strategy* |
| 4. Inclusion criteria | Specify the inclusion/exclusion criteria (e.g. in terms of population, language, year limits, type of publication, study type) | Methods - *Inclusion criteria* and Table 1 |
| 5. Data sources | Describe the information sources used (e.g. electronic databases (MEDLINE, EMBASE, CINAHL, PsycInfo), grey literature databases (digital thesis, policy reports), relevant organisational websites, experts, information specialists, generic web searches (Google Scholar) hand searching, reference lists) and when the searches conducted; provide the rationale for using the data sources | Methods – *Database and search strategy* |
| 6. Electronic Search strategy | Describe the literature search (e.g. provide electronic search strategies with population terms, clinical or health topic terms, experiential or social phenomena related terms, filters for qualitative research, and search limits) | Appendix S3 – *Search terms and strategy* |
| 7. Study screening methods | Describe the process of study screening and sifting (e.g. title, abstract and full text review, number of independent reviewers who screened studies) | Methods – *Data screening* |
| 8. Study characteristics | Present the characteristics of the included studies (e.g. year of publication, country, population, number of participants, data collection, methodology, analysis, research questions) | Appendix S5 includes table and summary of study characteristics in *Results* |
| 9. Study selection results | Identify the number of studies screened and provide reasons for study exclusion (e.g. for comprehensive searching, provide numbers of studies screened and reasons for exclusion indicated in a figure/flowchart; for iterative searching describe reasons for study exclusion and inclusion based on modifications to the research question and/or contribution to theory development) | Fig 1 - PRISMA flow diagram |
| 10. Rationale for appraisal | Describe the rationale and approach used to appraise the included studies or selected findings (e.g. assessment of conduct (validity and robustness), assessment of reporting (transparency), assessment of content and utility of the findings) | Methods – *Quality appraisal* |
| 11. Appraisal items | State the tools, frameworks and criteria used to appraise the studies or selected findings (e.g. Existing tools: CASP, QARI, COREQ, Mays and Pope [25]; reviewer developed tools; describe the domains assessed: research team, study design, data analysis and interpretations, reporting) | Methods – *Quality appraisal*  (CASP) |
| 12. Appraisal process | Indicate whether the appraisal was conducted independently by more than one reviewer and if consensus was required | Methods – *Quality appraisal* |
| 13. Appraisal results | Present results of the quality assessment and indicate which articles, if any, were weighted/excluded based on the assessment and give the rationale | Appendix S6 – Evaluation of study quality according to CASP |
| 14. Data extraction | Indicate which sections of the primary studies were analysed and how were the data extracted from the primary studies? (e.g. all text under the headings “results /conclusions” were extracted electronically and entered into a computer software) | Methods – *Data analysis* |
| 15. Software | State the computer software used, if any | Methods – *Data analysis*    NVivo 14 |
| 16. Number of reviewers | Identify who was involved in coding and analysis | Methods – *Data analysis* |
| 17. Coding | Describe the process for coding of data (e.g. line by line coding to search for concepts) | Methods – *Data analysis* |
| 18. Study comparison | Describe how were comparisons made within and across studies (e.g. subsequent studies were coded into pre-existing concepts, and new concepts were created when deemed necessary) | Methods – *Data analysis* and Figure 2 represents the thematic framework |
| 19. Derivation of themes | Explain whether the process of deriving the themes or constructs was inductive or deductive | Inductive process - see *Data analysis*, *thematic synthesis*, Appendix S7 and Figure 2 for thematic framework |
| 20. Quotations | Provide quotations from the primary studies to illustrate themes/constructs, and identify whether the quotations were participant quotations of the author’s interpretation | Findings -  Quotations and all sources given, also Appendix S7 include further exemplar quotes |
| 21. Synthesis output | Present rich, compelling and useful results that go beyond a summary of the primary studies (e.g. new interpretation, models of evidence, conceptual models, analytical framework, development of a new theory or construct) | Fig 2 – Map of meta-themes and sub-themes, findings and discussion |

*Tong A, Flemming K, McInnes E, Oliver S, Craig J. Enhancing transparency in reporting the synthesis of qualitative research: ENTREQ. BMC Med Res Methodol. 2012 Nov 27;12:181. doi: 10.1186/1471-2288-12-181. PMID: 23185978; PMCID: PMC3552766.
