# Supplementary material for: The experience of loneliness among people with psychosis: Qualitative meta-synthesis
Source: PLoS One. 2024 Dec 31;19(12):e0315763. doi: 10.1371/journal.pone.0315763 (PMC11687762; doi:10.1371/journal.pone.0315763)
Supplement: S3 Appendix — (DOCX) [file pone.0315763.s003.docx]

**Appendix S3: Search Strategy**

**Concept 1: Psychosis**

MeSH terms: Psychotic disorders (exp)

Psychosis OR psychotic disorder OR psychotic OR schiz* OR halluc* OR paran* OR delus*

AND

**Concept 2: Loneliness**

MeSH terms: Loneliness (exp)

Lonel* OR perceived social isolation OR social isolat* OR emotional isolat* OR social network* OR social support OR social contact OR social relation* OR social capita OR alienat* OR social interact* OR social activ* OR confiding OR confide OR interpersonal relation*

AND

**Concept 3: Qualitative method**

MeSH terms: Qualitative research (exp)

Qualitative* OR lived experience OR experience OR interview* OR focus group* OR IPA OR interpretive* OR grounded theory OR narrative* OR discourse* OR thematic* OR content analysis OR ethnograph* OR phenomenolog* OR hermeneutic OR semistructured* OR semi-structured OR unstructured* OR guided interview* OR guided discussion* OR group discussion OR transcribe OR open-ended OR mixed method OR mixed-method OR framework approach
