# Supplementary material for: The experience of loneliness among people with psychosis: Qualitative meta-synthesis
Source: PLoS One. 2024 Dec 31;19(12):e0315763. doi: 10.1371/journal.pone.0315763 (PMC11687762; doi:10.1371/journal.pone.0315763)
Supplement: S4 Appendix — (DOCX) [file pone.0315763.s004.docx]

**Appendix S4: List of studies excluded at full text screening**

| N | **Item** | **Screen on Full Text** |
| --- | --- | --- |
| 1 | A grounded theory study... (Hall) (ID:72082265) | -EXCLUDE on "did not focus on loneliness experience" *No focus on loneliness experience* |
| 2 | A Qualitative Evaluation... (Lavi-Rotenberg) (ID:72082091) | -EXCLUDE on "did not focus on loneliness experience" *A qualitative evaluation of an intervention* |
| 3 | Ackerson (2003) (ID:72082414) | -EXCLUDE on "did not meet the 50% threshold of psychosis sample" |
| 4 | Adler (2008) (ID:72019133) | -EXCLUDE on "dissertation & conference abstracts" |
| 5 | Aggelidou (2017) (ID:72017862) | -EXCLUDE on "did not include population with psychosis" |
| 6 | Agrest (2018) (ID:72012896) | -EXCLUDE on "did not meet the 50% threshold of psychosis sample" |
| 7 | Ahmad (2017) (ID:72013115) | -EXCLUDE on "solely using a quantitative method" *The abstract states semi-structured interview but reading methods it seems as if psychometric measures we used and quantitative data collected. Results reported numerically also, no quotes etc* |
| 8 | Aldersey (2015) (ID:72083079) | -EXCLUDE on "did not meet the 50% threshold of psychosis sample" |
| 9 | Alshowkan (2015) (ID:72013431) | -EXCLUDE on "did not focus on loneliness experience" |
| 10 | An exploration of patient... (Griffiths) (ID:72082799) | -EXCLUDE on "did not meet the 50% threshold of psychosis sample"  *although majority of participants included were diagnosed with psychosis, it is not clearly specifies which quotes were obtained from those participants.* |
| 11 | Angell (2000) (ID:72019793) | -EXCLUDE on "dissertation & conference abstracts" |
| 12 | Angell (2003) (ID:72014964) | -EXCLUDE on "did not focus on loneliness experience" |
| 13 | Angermeyer (2004) (ID:72014915) | -EXCLUDE on "did not meet the 50% threshold of psychosis sample" |
| 14 | Anke (2019) (ID:72012694) | -EXCLUDE on "did not include population with psychosis" |
| 15 | Anttinen (1983) (ID:72020272) | -EXCLUDE on "non-primary research" *This seems to be a book that compile a number of research papers.* |
| 16 | Arampatzi (2022) (ID:92341045) | -EXCLUDE on language |
| 17 | Archie (2013) (ID:72013843) | -EXCLUDE on "did not include population with psychosis" |
| 18 | Argentzell (2012) (ID:72016114) | -EXCLUDE on "did not meet the 50% threshold of psychosis sample" |
| 19 | Argentzell (2014) (ID:72081667) | -EXCLUDE on "solely using a quantitative method" |
| 20 | Asbo (2023) (ID:92339884) | -EXCLUDE on "dissertation & conference abstracts" |
| 21 | Aschbrenner (2012) (ID:72081669) | -EXCLUDE on "did not meet the 50% threshold of psychosis sample"  *Although majority of the participants had psychosis diagnosis, it is not specified which quotes were taken from those participants.* |
| 22 | Aschbrenner (2013) (ID:72081648) | -EXCLUDE on "did not focus on loneliness experience" *Exclude on focus - not about loneliness, about social support as facilitator to achieving health goals* |
| 23 | Aschbrenner (2016) (ID:72013209) | -EXCLUDE on "did not focus on loneliness experience" *There was no focus on the subjective experience of participants with schizophrenia.  It also wasn't specified if the information gathered was related to the participants' thoughts regarding their schizophrenia diagnosis or their substance use.* |
| 24 | Asher (2010) (ID:72016179) | -EXCLUDE on "did not focus on loneliness experience" |
| 25 | Baier (1996) (ID:72019943) | -EXCLUDE on "dissertation & conference abstracts" |
| 26 | Ball (2005) (ID:72016510) | -EXCLUDE on "did not meet the 50% threshold of psychosis sample"  *Although majority of the participants in the paper do have a psychosis diagnosis, it is not clear which quotes/information were gathered from those participants.* |
| 27 | Balogun-Katung (2021) (ID:72011796) | -EXCLUDE on "non-primary research" |
| 28 | Barrenger (2020) (ID:72012403) | -EXCLUDE on "did not meet the 50% threshold of psychosis sample" |
| 29 | Bassilios (2014) (ID:72013601) | -EXCLUDE on "did not focus on loneliness experience" |
| 30 | Beal (1998) (ID:72019868) | -EXCLUDE on "did not include population with psychosis" |
| 31 | Beal (1999) (ID:72016717) | -EXCLUDE on "did not meet the 50% threshold of psychosis sample" |
| 32 | Beal (2005) (ID:72083208) | -EXCLUDE on "did not focus on loneliness experience" |
| 33 | Beck (2002) (ID:72016624) | -EXCLUDE on "did not meet the 50% threshold of psychosis sample"  *9 out of 17 of the participants had a personality disorder diagnosis and the other 8 had psychosis diagnosis, meaning that participants with psychosis made up less than 50% of the participants in this paper.* |
| 34 | Becker (2008) (ID:72014645) | -EXCLUDE on "non-primary research" |
| 35 | Beckwith (2021) (ID:72081674) | -EXCLUDE on "did not focus on loneliness experience" *Although some of the themes mentioned in the paper are related to loneliness, social network and social support, it was mainly in relation to the EPS program rather than the general experience of loneliness that the participants go through.* |
| 36 | Bellass (2021) (ID:72012124) | -EXCLUDE on "did not meet the 50% threshold of psychosis sample"  *Out of 78 participants, only 26 had experienced psychosis (22 schizophrenia, 2 schizoaffective disorder, 2 depressive psychosis). Also study results not focused on loneliness.* |
| 37 | Bendel-Rozow (2020) (ID:72017417) | -EXCLUDE on "dissertation & conference abstracts" |
| 38 | Bird (2022) (ID:72011846) | -EXCLUDE on "did not include population with psychosis" |
| 39 | Bjornestad (2017) (ID:72013036) | -EXCLUDE on "did not focus on loneliness experience" |
| 40 | Bjornestad (2018) (ID:72017681) | -EXCLUDE on "did not focus on loneliness experience" |
| 41 | Blixen (2016) (ID:72013315) | -EXCLUDE on "did not meet the 50% threshold of psychosis sample"  *Less than 50% of the participants have a psychosis diagnosis.* |
| 42 | Bogen-Johnston (2019) (ID:72015591) | -EXCLUDE on "did not meet the 50% threshold of psychosis sample" |
| 43 | Boll (1999) (ID:72019830) | -EXCLUDE on "solely using a quantitative method" |
| 44 | Booysen (2021) (ID:72012098) | -EXCLUDE on "did not focus on loneliness experience" |
| 45 | Borba (2011) (ID:72014309) | -EXCLUDE on "did not meet the 50% threshold of psychosis sample"  *Less than 50% of the participants have a psychosis diagnosis.* |
| 46 | Botero-Rodriguez (2021) (ID:72012202) | -EXCLUDE on "did not meet the 50% threshold of psychosis sample"  *Less than 50% of the participants had a psychosis diagnosis.* |
| 47 | Bourdeau (2015) (ID:72013537) | -EXCLUDE on "solely using a quantitative method" |
| 48 | Boydell (2002) (ID:72015036) | -EXCLUDE on "did not meet the 50% threshold of psychosis sample"  *Not able to tell if participants met 50% threshold of psychosis sample. Only states that participant either had unipolar depression, manic depression or schizophrenia* |
| 49 | Boydell (2006) (ID:72014776) | -EXCLUDE on "did not focus on loneliness experience" |
| 50 | Boydell (2013) (ID:72013844) | -EXCLUDE on "did not include population with psychosis" |
| 51 | Boyle (2015) (ID:72013495) | -EXCLUDE on "dissertation & conference abstracts" |
| 52 | Bradshaw (1998) (ID:72082232) | -EXCLUDE on "solely using a quantitative method" *Semi-structured interviews were conducted but there is no section on analysis and it seems as if a content analysis of a befriending intervention was conducted. Could also be excluded on focus, as more of an evaluation of an intervention than focus on peoples experiences of loneliness* |
| 53 | Bril-Barniv (2017) (ID:72013117) | -EXCLUDE on "did not meet the 50% threshold of psychosis sample" |
| 54 | Bromley (2013) (ID:72083126) | -EXCLUDE on "did not meet the 50% threshold of psychosis sample"  *13 out of 30 participants had psychotic disorders* |
| 55 | Brooke (2018) (ID:72012919) | -EXCLUDE on "did not meet the 50% threshold of psychosis sample" |
| 56 | Brooke (2020) (ID:72012519) | -EXCLUDE on "did not meet the 50% threshold of psychosis sample" |
| 57 | Brooke (2022) (ID:92341064) | -EXCLUDE on "did not focus on loneliness experience" |
| 58 | Brooke-Sumner (2017) (ID:72017868) | -EXCLUDE on "did not focus on loneliness experience" |
| 59 | Brooke-Sumner (2018) (ID:72012784) | -EXCLUDE on "did not focus on loneliness experience" |
| 60 | Brooks (2020) (ID:72083059) | -EXCLUDE on "did not meet the 50% threshold of psychosis sample"  *The paper didn't specify the percentage of participants with a psychosis diagnosis.* |
| 61 | Brooks (2021) (ID:72012200) | -EXCLUDE on "did not meet the 50% threshold of psychosis sample" |
| 62 | Brown (2010) (ID:72014439) | -EXCLUDE on "non-primary research" |
| 63 | Browne (2005) (ID:72014742) | -EXCLUDE on "did not focus on loneliness experience" |
| 64 | Buchanan (2020) (ID:72012387) | -EXCLUDE on "did not meet the 50% threshold of psychosis sample" |
| 65 | Buchberg (2015) (ID:72018160) | -EXCLUDE on "did not include population with psychosis" |
| 66 | Buizza (2007) (ID:72014724) | -EXCLUDE on "did not focus on loneliness experience" |
| 67 | Burton (2016) (ID:72013250) | -EXCLUDE on "dissertation & conference abstracts" |
| 68 | Butcher (2020) (ID:72012324) | -EXCLUDE on "did not focus on loneliness experience" |
| 69 | Byrne (2010) (ID:72014487) | -EXCLUDE on "did not include population with psychosis" |
| 70 | Cabassa (2013) (ID:72013848) | -EXCLUDE on "did not meet the 50% threshold of psychosis sample"  *5 out of 16 had schizophrenia* |
| 71 | Carpenter-Song (2014) (ID:72083390) | -EXCLUDE on "did not include population with psychosis" |
| 72 | Carpentier (2006) (ID:72014793) | -EXCLUDE on "did not focus on loneliness experience" |
| 73 | Carswell (2022) (ID:92340167) | -EXCLUDE on "did not focus on loneliness experience" |
| 74 | Cechnicki (2011) (ID:72014236) | -EXCLUDE on "solely using a quantitative method" |
| 75 | Chadwick (2006) (ID:72014777) | -EXCLUDE on "non-primary research" |
| 76 | Chadzynska (2014) (ID:72018219) | -EXCLUDE on "solely using a quantitative method" |
| 77 | Chai (2021) (ID:72012258) | -EXCLUDE on "did not meet the 50% threshold of psychosis sample" |
| 78 | Chan (2014) (ID:72013803) | -EXCLUDE on "non-primary research" |
| 79 | Chandler (1996) (ID:72016779) | -EXCLUDE on "solely using a quantitative method" |
| 80 | Charette-Dussault (2019) (ID:72012575) | -EXCLUDE on "non-primary research" |
| 81 | Charles (2012) (ID:72018582) | -EXCLUDE on "non-primary research" |
| 82 | Chee (2019) (ID:72012457) | -EXCLUDE on "did not focus on loneliness experience" |
| 83 | Chen (2013) (ID:72013999) | -EXCLUDE on "did not focus on loneliness experience" |
| 84 | Chen (2017) (ID:72013070) | -EXCLUDE on "did not meet the 50% threshold of psychosis sample" |
| 85 | Chen (2020) (ID:72012275) | -EXCLUDE on "loneliness as a fleeting topic" |
| 86 | Cheng (2016) (ID:72017996) | -EXCLUDE on "did not focus on loneliness experience" |
| 87 | Chernomas (2008) (ID:72014597) | -EXCLUDE on "did not focus on loneliness experience" |
| 88 | Chevalier (2018) (ID:72083007) | -EXCLUDE on "did not meet the 50% threshold of psychosis sample" |
| 89 | Chin (2009) (ID:72016297) | -EXCLUDE on "did not focus on loneliness experience" |
| 90 | Chronister (2015) (ID:72013342) | -EXCLUDE on "did not meet the 50% threshold of psychosis sample"  *Less than 50% had schizophrenia/schizoaffective disorder* |
| 91 | Chung (2015) (ID:72013535) | -EXCLUDE on "did not meet the 50% threshold of psychosis sample"  *Although participants with a psychosis diagnosis made up more than 50% of the, it wasn't specified which qoutes were gathered from those participants.* |
| 92 | Chung-Zou (2023) (ID:92340877) | -EXCLUDE on "did not meet the 50% threshold of psychosis sample" |
| 93 | Cimo (2018) (ID:72015669) | -EXCLUDE on "did not meet the 50% threshold of psychosis sample" |
| 94 | Cimo (2020) (ID:72083380) | -EXCLUDE on "did not focus on loneliness experience" |
| 95 | Cinnirella (1999) (ID:72015155) | -EXCLUDE on "did not include population with psychosis" |
| 96 | Cloutier (2023) (ID:92340697) | -EXCLUDE on "did not focus on loneliness experience" |
| 97 | Coffey (2008) (ID:72014592) | -EXCLUDE on "did not focus on loneliness experience" |
| 98 | Cohen (1974) (ID:72020377) | -EXCLUDE on "non-primary research" |
| 99 | Cohen (1978) (ID:72020333) | -EXCLUDE on "solely using a quantitative method" |
| 100 | Cohen (2017) (ID:72013026) | -EXCLUDE on "did not focus on loneliness experience" |
| 101 | Cole (1995) (ID:72015212) | -EXCLUDE on "solely using a quantitative method" |
| 102 | Connell (2015) (ID:72013400) | -EXCLUDE on "did not focus on loneliness experience" *Loss of friendships was talked about but this was more objective whereas subjective feelings of loneliness were not focused on.* |
| 103 | Contreras (2016) (ID:72083031) | -EXCLUDE on "did not focus on loneliness experience" |
| 104 | Contreras (2016) (ID:72082980) | -EXCLUDE on "did not meet the 50% threshold of psychosis sample"  *Less than 50% of the participants were diagnosed with psychosis.* |
| 105 | Coombe (1995) (ID:72019963) | -EXCLUDE on "did not include population with psychosis" |
| 106 | Corin (1990) (ID:72015262) | -EXCLUDE on "did not focus on loneliness experience" |
| 107 | Corin (1992) (ID:72015253) | -EXCLUDE on "single case studies" |
| 108 | Corring (2007) (ID:72016423) | -EXCLUDE on "did not meet the 50% threshold of psychosis sample" |
| 109 | Cotter (2019) (ID:72012574) | -EXCLUDE on "did not include population with psychosis" *According to the paper non of the participants had passed the threshold for a psychosis diagnosis at the time the study was conducted.* |
| 110 | Courtet (2018) (ID:72012803) | -EXCLUDE on language |
| 111 | Cresswell (1992) (ID:72015246) | -EXCLUDE on "solely using a quantitative method" *Data reported numerically* |
| 112 | Crotty (1986) (ID:72020204) | -EXCLUDE on "solely using a quantitative method" |
| 113 | Davidson (1995) (ID:72019989) | -EXCLUDE on "loneliness as a fleeting topic" |
| 114 | Davidson (2001) (ID:72082175) | -EXCLUDE on "did not meet the 50% threshold of psychosis sample" |
| 115 | Davies (1989) (ID:72016901) | -EXCLUDE on "solely using a quantitative method" |
| 116 | Davis (2005) (ID:72014849) | -EXCLUDE on "did not meet the 50% threshold of psychosis sample"  *Although participants with a schizophrenia diagnosis made up the majority of the participants in the paper, it wasn't specified which quotes were obtained from the participants with schizophrenia.* |
| 117 | De Pater (2012) (ID:72014168) | -EXCLUDE on "dissertation & conference abstracts" |
| 118 | de Sousa (2015) (ID:72013375) | -EXCLUDE on "solely using a quantitative method" |
| 119 | De Vries (2013) (ID:72013907) | -EXCLUDE on "non-primary research" |
| 120 | Delespaul (1987) (ID:72015279) | -EXCLUDE on "solely using a quantitative method" |
| 121 | Depp (1986) (ID:72020205) | -EXCLUDE on "solely using a quantitative method" |
| 122 | Deshpande (2021) (ID:72012282) | -EXCLUDE on "non-primary research" |
| 123 | Dias (2011) (ID:72014233) | -EXCLUDE on language |
| 124 | Diaz-Caneja (2004) (ID:72014905) | -EXCLUDE on "did not focus on loneliness experience" |
| 125 | Digman (2004) (ID:72019591) | -EXCLUDE on "dissertation & conference abstracts" |
| 126 | Dinos (2004) (ID:72016570) | -EXCLUDE on "did not focus on loneliness experience" |
| 127 | Dittmann (1990) (ID:72016888) | -EXCLUDE on "did not focus on loneliness experience" |
| 128 | Dixon (2017) (ID:72013102) | -EXCLUDE on "dissertation & conference abstracts" |
| 129 | Dobbins (2020) (ID:72082105) | -EXCLUDE on "did not meet the 50% threshold of psychosis sample" |
| 130 | Dolman (2011) (ID:72014262) | -EXCLUDE on "dissertation & conference abstracts" |
| 131 | Dominguez (2013) (ID:72013983) | -EXCLUDE on "dissertation & conference abstracts" |
| 132 | Donnals (2014) (ID:72018228) | -EXCLUDE on "dissertation & conference abstracts" |
| 133 | Donnelly (2011) (ID:72018800) | -EXCLUDE on "did not include population with psychosis" |
| 134 | Doran (2023) (ID:92339823) | -EXCLUDE on "did not meet the 50% threshold of psychosis sample" |
| 135 | Doucet (2012) (ID:72014213) | -EXCLUDE on "did not meet the 50% threshold of psychosis sample" |
| 136 | Doucet (2013) (ID:72018398) | -EXCLUDE on "dissertation & conference abstracts" *Dissertation* |
| 137 | Dozier (1988) (ID:72016915) | -EXCLUDE on "solely using a quantitative method" |
| 138 | Drake (2014) (ID:72018262) | -EXCLUDE on "non-primary research" |
| 139 | Dulek (2022) (ID:72017248) | -EXCLUDE on "dissertation & conference abstracts" |
| 140 | Dunn (2010) (ID:72083158) | -EXCLUDE on "did not focus on loneliness experience" |
| 141 | Dutta (2019) (ID:72012701) | -EXCLUDE on "did not focus on loneliness experience" |
| 142 | Earl (2011) (ID:72014331) | -EXCLUDE on "did not include population with psychosis" |
| 143 | Edmondson (2018) (ID:72012862) | -EXCLUDE on "did not meet the 50% threshold of psychosis sample"  *Also, could be excluded on focus* |
| 144 | Edwards (2005) (ID:72014860) | -EXCLUDE on "did not focus on loneliness experience" |
| 145 | Eisenstadt (2010) (ID:72014446) | -EXCLUDE on "did not focus on loneliness experience" |
| 146 | Eklund (2003) (ID:72014882) | -EXCLUDE on "did not focus on loneliness experience" |
| 147 | Eliacin (2013) (ID:72013991) | -EXCLUDE on "did not meet the 50% threshold of psychosis sample" |
| 148 | Elmoudden (2019) (ID:72083517) | -EXCLUDE on "non-primary research" |
| 149 | England (2009) (ID:72014525) | -EXCLUDE on "did not include population with psychosis" |
| 150 | Engqvist (2013) (ID:72014010) | -EXCLUDE on "did not focus on loneliness experience" *While loneliness may be inferred from quotes and was mentioned briefly, there is no clear focus on/dedication to subjective experience of loneliness.* |
| 151 | Estrella (2019) (ID:72082243) | -EXCLUDE on "did not focus on loneliness experience" |
| 152 | Experience of psychosis... (Lebovitz) (ID:72082220) | -EXCLUDE on "did not focus on loneliness experience" *Paper focus on the impact of COVID-19 on voice-content, not on loneliness. Touches on social isolation but not enough data* |
| 153 | Faigin (2015) (ID:72081624) | -EXCLUDE on "did not include population with psychosis" |
| 154 | Faith (2019) (ID:72017542) | -EXCLUDE on "did not focus on loneliness experience" |
| 155 | Falloon (1981) (ID:72020304) | -EXCLUDE on "did not focus on loneliness experience" |
| 156 | Farrelly (2015) (ID:72015824) | -EXCLUDE on "did not meet the 50% threshold of psychosis sample" |
| 157 | Faulkner (1999) (ID:72019843) | -EXCLUDE on "non-primary research" |
| 158 | Feeney (2021) (ID:72011871) | -EXCLUDE on "did not meet the 50% threshold of psychosis sample" |
| 159 | Fekete (2022) (ID:92340487) | -EXCLUDE on "dissertation & conference abstracts" |
| 160 | Fernandes (2020) (ID:72017392) | -EXCLUDE on "did not include population with psychosis" *voice hearers however not diagnosed or screened* |
| 161 | Fernandes (2021) (ID:72012037) | -EXCLUDE on "did not include population with psychosis" |
| 162 | Firmin (2015) (ID:72013360) | -EXCLUDE on "did not focus on loneliness experience" |
| 163 | Firmin (2016) (ID:72013302) | -EXCLUDE on "did not focus on loneliness experience" |
| 164 | Firth (2016) (ID:72013241) | -EXCLUDE on "did not focus on loneliness experience" *The main objective of the paper was to assess the impact of exercise on patients after a first episode of psychosis; neither of the two mentioned themes focused on loneliness or other related concepts* |
| 165 | Fixsen (2021) (ID:72011934) | -EXCLUDE on "non-primary research" |
| 166 | Florence (2021) (ID:72011855) | -EXCLUDE on "did not meet the 50% threshold of psychosis sample"  *Not clear what the participants diagnosis was* |
| 167 | Fogarty (2005) (ID:72016512) | -EXCLUDE on "did not focus on loneliness experience" *An evaluation of an exercise programme, all 6 participants diagnosed with schizophrenia but focus of study is not on loneliness. The overall aim of the study was to determine the impact of a structured exercise program on the physical and psychological well-being* |
| 168 | Forchuk (2015) (ID:72015835) | -EXCLUDE on "did not focus on loneliness experience" |
| 169 | Forrest (1971) (ID:72017079) | -EXCLUDE on "solely using a quantitative method" |
| 170 | Forrester-Jones (2008) (ID:72083109) | -EXCLUDE on "loneliness as a fleeting topic" |
| 171 | Fortuna (2018) (ID:72017669) | -EXCLUDE on "did not meet the 50% threshold of psychosis sample" |
| 172 | Fortuna (2019) (ID:72012652) | -EXCLUDE on "did not meet the 50% threshold of psychosis sample" |
| 173 | Fortuna (2022) (ID:72011856) | -EXCLUDE on "solely using a quantitative method" *Feasibility trial for a digital peer support intervention. Used quantitative data to compare baseline and 12 week follow up.* |
| 174 | Fossey (2020) (ID:72017442) | -EXCLUDE on "did not meet the 50% threshold of psychosis sample"  *Only 41% with psychotic disorders* |
| 175 | Fox (2017) (ID:72017906) | -EXCLUDE on "did not focus on loneliness experience" |
| 176 | Fox (2020) (ID:72017425) | -EXCLUDE on "did not focus on loneliness experience" |
| 177 | Fox (2021) (ID:72017232) | -EXCLUDE on "did not focus on loneliness experience" |
| 178 | Francoeur (2010) (ID:72016202) | -EXCLUDE on "did not focus on loneliness experience" |
| 179 | Frank (2012) (ID:72018649) | -EXCLUDE on "did not focus on loneliness experience" |
| 180 | Franz (2012) (ID:72016067) | -EXCLUDE on "did not focus on loneliness experience" |
| 181 | Friedrich (2014) (ID:72018273) | -EXCLUDE on "non-primary research" |
| 182 | Friesen (2021) (ID:72082848) | -EXCLUDE on "did not focus on loneliness experience" |
| 183 | Frounfelker (2010) (ID:72014368) | -EXCLUDE on "did not meet the 50% threshold of psychosis sample"  *Participants with psychosis made up less than 50% of the participants included in the paper.* |
| 184 | Fusar-Poli (2022) (ID:92340391) | -EXCLUDE on "non-primary research" |
| 185 | Gabrielian (2019) (ID:72081642) | -EXCLUDE on "did not focus on loneliness experience" *Also did not meet 50% psychosis threshold* |
| 186 | Gaine (2022) (ID:72082067) | -EXCLUDE on "did not meet the 50% threshold of psychosis sample" |
| 187 | Gandhi (2019) (ID:72012601) | -EXCLUDE on "did not focus on loneliness experience" |
| 188 | Gard (2009) (ID:72014553) | -EXCLUDE on "non-primary research" |
| 189 | Gee (2019) (ID:72012573) | -EXCLUDE on "did not focus on loneliness experience" |
| 190 | Georgaca (2018) (ID:72017725) | -EXCLUDE on "did not focus on loneliness experience" |
| 191 | Georgaca (2019) (ID:72017541) | -EXCLUDE on "did not focus on loneliness experience" |
| 192 | Ghebrehiwet (2020) (ID:72012529) | -EXCLUDE on "did not meet the 50% threshold of psychosis sample" |
| 193 | Giacco (2022) (ID:92340383) | -EXCLUDE on "non-primary research" |
| 194 | Gill (2022) (ID:72017305) | -EXCLUDE on "dissertation & conference abstracts" |
| 195 | Girard (2021) (ID:72081872) | -EXCLUDE on "loneliness as a fleeting topic" |
| 196 | Glover (2014) (ID:72013641) | -EXCLUDE on "did not focus on loneliness experience" |
| 197 | Goldberg (2004) (ID:72014934) | -EXCLUDE on "non-primary research" |
| 198 | Goldfarb (2021) (ID:72012148) | -EXCLUDE on "did not include population with psychosis" |
| 199 | Gong (2023) (ID:92340949) | -EXCLUDE on "did not focus on loneliness experience" |
| 200 | Gonzalez-Torres (2007) (ID:72014691) | -EXCLUDE on "did not meet the 50% threshold of psychosis sample"  *The participants who had a psychosis diagnosis were 18 out of the 44 participants (the rest were family members) meaning that it doesn't pass the 50% threshold.* |
| 201 | Gooding (2019) (ID:72012616) | -EXCLUDE on "did not focus on loneliness experience" |
| 202 | Graham (2014) (ID:72013577) | -EXCLUDE on "did not meet the 50% threshold of psychosis sample" |
| 203 | Grassi (2022) (ID:72011764) | -EXCLUDE on "did not focus on loneliness experience" |
| 204 | Green (2012) (ID:72014212) | -EXCLUDE on "did not meet the 50% threshold of psychosis sample"  *Participants with a psychosis diagnosis only made up 42%* |
| 205 | Green (2015) (ID:72015877) | -EXCLUDE on "did not meet the 50% threshold of psychosis sample" |
| 206 | Gurcan (2021) (ID:72012001) | -EXCLUDE on "non-primary research" |
| 207 | Habtamu (2015) (ID:72081787) | -EXCLUDE on "did not meet the 50% threshold of psychosis sample" |
| 208 | Hackethal (2013) (ID:72013814) | -EXCLUDE on "did not meet the 50% threshold of psychosis sample" |
| 209 | Hailemariam (2017) (ID:72081786) | -EXCLUDE on "did not meet the 50% threshold of psychosis sample" |
| 210 | Hamilton (2014) (ID:72013595) | -EXCLUDE on "did not meet the 50% threshold of psychosis sample" |
| 211 | Hampson (2020) (ID:72017408) | -EXCLUDE on "did not meet the 50% threshold of psychosis sample" |
| 212 | Han (2021) (ID:72011995) | -EXCLUDE on "did not include population with psychosis" |
| 213 | Hansen (2001) (ID:72015057) | -EXCLUDE on "did not focus on loneliness experience" |
| 214 | Harris (1990) (ID:72015265) | -EXCLUDE on "did not focus on loneliness experience" |
| 215 | Harris (2005) (ID:72014847) | -EXCLUDE on "did not meet the 50% threshold of psychosis sample"  *On the basis of chart diagnoses, 21 (78 percent) women had a primary affective disorder diagnosis, three (11 percent) had a diagnosis of psychotic spectrum disorders, and three (11 percent) had an anxiety disorder.* |
| 216 | Harris (2012) (ID:72014207) | -EXCLUDE on "did not focus on loneliness experience" |
| 217 | Harris (2018) (ID:72012939) | -EXCLUDE on "dissertation & conference abstracts" |
| 218 | Hartley (2014) (ID:72013642) | -EXCLUDE on "non-primary research" |
| 219 | Hassanat (2018) (ID:72012911) | -EXCLUDE on "dissertation & conference abstracts" |
| 220 | Hawkins (2007) (ID:72081844) | -EXCLUDE on "did not meet the 50% threshold of psychosis sample" |
| 221 | Hayden-Lewis (2014) (ID:72013712) | -EXCLUDE on "dissertation & conference abstracts" |
| 222 | Heasman (2004) (ID:72014917) | -EXCLUDE on "did not meet the 50% threshold of psychosis sample"  *Also not a focus on loneliness* |
| 223 | Hedlund (2010) (ID:72016192) | -EXCLUDE on "did not focus on loneliness experience" |
| 224 | Helu-Brown (2023) (ID:92340705) | -EXCLUDE on "loneliness as a fleeting topic" |
| 225 | Henriksen (2017) (ID:72013118) | -EXCLUDE on "non-primary research" |
| 226 | Hensley (2002) (ID:72015019) | -EXCLUDE on "non-primary research" |
| 227 | Hernandez (2017) (ID:72013138) | -EXCLUDE on "did not meet the 50% threshold of psychosis sample"  *The number of participants with a psychosis diagnosis was 14 out of the 34 participants (the remaining were family members)* |
| 228 | Hernandez (2019) (ID:72012768) | -EXCLUDE on "did not focus on loneliness experience" |
| 229 | Heron (2012) (ID:72014041) | -EXCLUDE on "did not focus on loneliness experience" |
| 230 | Herrera (2022) (ID:92340225) | -EXCLUDE on "did not focus on loneliness experience" |
| 231 | Herrman (2004) (ID:72014939) | -EXCLUDE on "solely using a quantitative method" |
| 232 | Heydari (2017) (ID:72012998) | -EXCLUDE on "did not meet the 50% threshold of psychosis sample" |
| 233 | Higgins (2017) (ID:72012988) | -EXCLUDE on "did not include population with psychosis" |
| 234 | Hodes (2005) (ID:72014846) | -EXCLUDE on "non-primary research" |
| 235 | Holgersen (2023) (ID:92339936) | -EXCLUDE on "did not focus on loneliness experience" |
| 236 | Holttum (2019) (ID:72081886) | -EXCLUDE on "non-primary research" |
| 237 | Honary (2018) (ID:72082815) | -EXCLUDE on "did not meet the 50% threshold of psychosis sample"  *Of the caregivers, two provided care to a spouse, three to a sibling and two to a child (over 18). One participant both had experience of providing care to a parent with SMI and was a patient themselves. The final three participants were patients.* |
| 238 | Howes (2023) (ID:92340159) | -EXCLUDE on "did not focus on loneliness experience" |
| 239 | Hsiao (2004) (ID:72014901) | -EXCLUDE on "did not focus on loneliness experience" |
| 240 | Hubbard (2013) (ID:72018397) | -EXCLUDE on "dissertation & conference abstracts" |
| 241 | Hughes (2009) (ID:72014559) | -EXCLUDE on "did not meet the 50% threshold of psychosis sample"  *The participants with a psychosis diagnosis were only 2 out of the 12 participants in the paper.* |
| 242 | Huguelet (2015) (ID:72018197) | -EXCLUDE on "solely using a quantitative method" |
| 243 | Hultsjo (2013) (ID:72014003) | -EXCLUDE on "loneliness as a fleeting topic" |
| 244 | Hultsjo (2022) (ID:72011827) | -EXCLUDE on "did not focus on loneliness experience" |
| 245 | Huszonek (1987) (ID:72020180) | -EXCLUDE on "non-primary research" |
| 246 | Ienciu (2010) (ID:72016176) | -EXCLUDE on "did not focus on loneliness experience" |
| 247 | Irarrazaval (2014) (ID:72018211) | -EXCLUDE on "did not focus on loneliness experience" |
| 248 | Isik (2020) (ID:72012542) | -EXCLUDE on "loneliness as a fleeting topic" |
| 249 | Itten (2006) (ID:72019465) | -EXCLUDE on "dissertation & conference abstracts" |
| 250 | Jabardo-Camprubi (2022) (ID:92340303) | -EXCLUDE on "did not focus on loneliness experience" |
| 251 | Jackson (2011) (ID:72016165) | -EXCLUDE on "did not meet the 50% threshold of psychosis sample"  *Participants with and without a psychiatric diagnosis were included in the study - not sure how many had psychosis diagnosis as not reported* |
| 252 | Jackson (2021) (ID:72011851) | -EXCLUDE on "did not include population with psychosis" |
| 253 | Jackson-Blott (2019) (ID:72012620) | -EXCLUDE on "did not focus on loneliness experience" |
| 254 | Jacob (1967) (ID:72017096) | -EXCLUDE on "did not focus on loneliness experience" |
| 255 | Jacobs (1974) (ID:72017056) | -EXCLUDE on "solely using a quantitative method" |
| 256 | Jacques (2019) (ID:72012765) | -EXCLUDE on "did not focus on loneliness experience" *Filter model is the focus - paper relates more to coping/ barriers to coping* |
| 257 | Jameel (2020) (ID:72017278) | -EXCLUDE on "did not focus on loneliness experience" *The main topic discussed was the role of social relations in the treatment process of patients with schizophrenia, it didn't really discusses the loneliness experience of the participants.* |
| 258 | Jankowski (2023) (ID:92339814) | -EXCLUDE on "did not focus on loneliness experience" |
| 259 | Jansen (2015) (ID:72013392) | -EXCLUDE on "did not focus on loneliness experience" |
| 260 | Jansen (2016) (ID:72013246) | -EXCLUDE on "did not focus on loneliness experience" |
| 261 | Jaramillo (2012) (ID:72018577) | -EXCLUDE on "dissertation & conference abstracts" |
| 262 | Jenkins (2008) (ID:72014654) | -EXCLUDE on "non-primary research" |
| 263 | Jepson (2019) (ID:72015570) | -EXCLUDE on "non-primary research" |
| 264 | Jerwood (2021) (ID:72012024) | -EXCLUDE on "did not focus on loneliness experience" |
| 265 | Johnston (2014) (ID:72018363) | -EXCLUDE on "did not include population with psychosis" |
| 266 | Johnstone (2009) (ID:72014538) | -EXCLUDE on "did not focus on loneliness experience" |
| 267 | Jong (2006) (ID:72014785) | -EXCLUDE on "solely using a quantitative method" |
| 268 | Jordan (2020) (ID:72017407) | -EXCLUDE on "did not focus on loneliness experience" *Non of the themes mentioned in the qualitative part of the paper conveyed the participants' subjective experience with loneliness.* |
| 269 | Jordan (2021) (ID:72017351) | -EXCLUDE on "dissertation & conference abstracts" |
| 270 | Jormfeldt (2012) (ID:72018682) | -EXCLUDE on "did not focus on loneliness experience" |
| 271 | Joung (2021) (ID:72012078) | -EXCLUDE on "did not focus on loneliness experience" *There wasn't any real focus on the loneliness experience itself but rather just a description of the participants life story and what they have been through.* |
| 272 | Judge (2008) (ID:72016348) | -EXCLUDE on "did not focus on loneliness experience" |
| 273 | Kaewprom (2011) (ID:72014259) | -EXCLUDE on "did not include population with psychosis" |
| 274 | Kalofonos (2023) (ID:92339852) | -EXCLUDE on "loneliness as a fleeting topic" |
| 275 | Kaltenboeck (2023) (ID:92339980) | -EXCLUDE on "loneliness as a fleeting topic" |
| 276 | Kaltiala-Heino (2013) (ID:72013838) | -EXCLUDE on "did not focus on loneliness experience" |
| 277 | Kamens (2017) (ID:72017878) | -EXCLUDE on "dissertation & conference abstracts" |
| 278 | Kane (2019) (ID:72012684) | -EXCLUDE on "non-primary research" |
| 279 | Karanci (2017) (ID:72013112) | -EXCLUDE on "did not focus on loneliness experience" *The paper focuses on social support and not lack of social support or loneliness, there is a little relevant data but in comparison to irrelevant data, it is not much at all.  As much as participants wanted their social network to interact with them either by just saying ‘hi’ or ‘asking them how they are doing’, the absence of these kind of interactions was mentioned as unsupportive: I mean, my father never calls me and asks me how I am doing. I have been to hospital four times, up to this current hospital. Not even once did he visit me. (Participant 3, female, 44) Do they not care about me or do I care too much. I do not see their interest in me in return. People don’t care about greeting at all. (Participant 18, female, 54)* |
| 280 | Karanci (2022) (ID:72011797) | -EXCLUDE on "did not focus on loneliness experience" |
| 281 | Kashiwagi (2020) (ID:72012308) | -EXCLUDE on "solely using a quantitative method" |
| 282 | Katz (2022) (ID:72082504) | -EXCLUDE on "did not meet the 50% threshold of psychosis sample" |
| 283 | Kavanaugh (2015) (ID:72018183) | -EXCLUDE on "did not include population with psychosis" |
| 284 | Keith (1993) (ID:72015237) | -EXCLUDE on "non-primary research" |
| 285 | Kennedy-Jones (2005) (ID:72014867) | -EXCLUDE on "did not focus on loneliness experience" |
| 286 | Khalid (2023) (ID:92341320) | -EXCLUDE on "did not focus on loneliness experience" |
| 287 | Khare (2020) (ID:72012448) | -EXCLUDE on "solely using a quantitative method" *Although the method of data collection was semi-structured interview based, the data was presented in a quantitib=ve manner.* |
| 288 | Kidd (2016) (ID:72013224) | -EXCLUDE on "loneliness as a fleeting topic" |
| 289 | Kilian (2011) (ID:72014271) | -EXCLUDE on "dissertation & conference abstracts" |
| 290 | Kinderman (2015) (ID:72013419) | -EXCLUDE on "non-primary research" |
| 291 | Kistler (1977) (ID:72017024) | -EXCLUDE on "non-primary research" |
| 292 | Knafo (2020) (ID:72012545) | -EXCLUDE on "non-primary research" |
| 293 | Ko (2021) (ID:72012222) | -EXCLUDE on "did not focus on loneliness experience" |
| 294 | Kohn (1997) (ID:72019907) | -EXCLUDE on "did not include population with psychosis" |
| 295 | Kokanovic (2018) (ID:72015623) | -EXCLUDE on "did not meet the 50% threshold of psychosis sample" |
| 296 | Koletsi (2009) (ID:72016276) | -EXCLUDE on "did not meet the 50% threshold of psychosis sample"  *Although the population does include a majority of patients with a psychosis diagnosis(33 out of 49), it is not specified which information/quotes are gathered from the patients with a psychosis diagnosis.* |
| 297 | Kollmann (2011) (ID:72014275) | -EXCLUDE on "solely using a quantitative method" |
| 298 | Kolvin (1971) (ID:72017076) | -EXCLUDE on "solely using a quantitative method" |
| 299 | Kordas (2015) (ID:72015828) | -EXCLUDE on "did not meet the 50% threshold of psychosis sample" |
| 300 | Koschorke (2014) (ID:72013570) | -EXCLUDE on "did not focus on loneliness experience" |
| 301 | Koslander (2021) (ID:72083197) | -EXCLUDE on "did not meet the 50% threshold of psychosis sample" |
| 302 | Kranke (2010) (ID:72081835) | -EXCLUDE on "did not meet the 50% threshold of psychosis sample" |
| 303 | Kreyenbuhl (2019) (ID:72012703) | -EXCLUDE on "did not meet the 50% threshold of psychosis sample" |
| 304 | Kriegel (2019) (ID:72012603) | -EXCLUDE on "did not meet the 50% threshold of psychosis sample"  *A majority of participants reported diagnoses of schizophrenia, schizoaffective, or bipolar disorders (n = 22) = 61% Not sure how many with BD diagnosis?* |
| 305 | Kristoffersen (2000) (ID:72015101) | -EXCLUDE on "did not include population with psychosis" |
| 306 | Krupa (2000) (ID:72019789) | -EXCLUDE on "dissertation & conference abstracts" |
| 307 | Krupchanka (2016) (ID:72015799) | -EXCLUDE on "did not include population with psychosis" *Relatives of people diagnosed with schizophrenia* |
| 308 | Krutis (2015) (ID:72018070) | -EXCLUDE on "dissertation & conference abstracts" |
| 309 | Kuek (2022) (ID:92341140) | -EXCLUDE on "did not meet the 50% threshold of psychosis sample" |
| 310 | Laing (2016) (ID:72013317) | -EXCLUDE on "did not focus on loneliness experience" |
| 311 | Lake (2020) (ID:72017443) | -EXCLUDE on "dissertation & conference abstracts" |
| 312 | Lal (2018) (ID:72012833) | -EXCLUDE on "did not focus on loneliness experience" |
| 313 | Laliberte-Rudman (2000) (ID:72015027) | -EXCLUDE on "loneliness as a fleeting topic" |
| 314 | Landeen (2000) (ID:72019788) | -EXCLUDE on "dissertation & conference abstracts" |
| 315 | Lariviere (2010) (ID:72082994) | -EXCLUDE on "did not meet the 50% threshold of psychosis sample" |
| 316 | Larkings (2017) (ID:72082603) | -EXCLUDE on "did not meet the 50% threshold of psychosis sample" |
| 317 | Larsen (2019) (ID:72012663) | -EXCLUDE on "did not focus on loneliness experience" |
| 318 | Larsen-Barr (2021) (ID:72012294) | -EXCLUDE on "did not meet the 50% threshold of psychosis sample" |
| 319 | Lauveng (2015) (ID:72018076) | -EXCLUDE on "did not meet the 50% threshold of psychosis sample" |
| 320 | Lauveng (2016) (ID:72013308) | -EXCLUDE on "did not meet the 50% threshold of psychosis sample" |
| 321 | Lavis (2014) (ID:72013720) | -EXCLUDE on "dissertation & conference abstracts" |
| 322 | Lawrence (2021) (ID:72012220) | -EXCLUDE on "did not focus on loneliness experience" |
| 323 | Le (2022) (ID:72017259) | -EXCLUDE on "did not focus on loneliness experience" *The topic wasn't focused on social relations in general but rather the participants' experience and personal thoughts on peer support work. There wasn't any illustration of the subjective experience of the participants with loneliness.* |
| 324 | Lebovitz (2023) (ID:92340040) | -EXCLUDE on "loneliness as a fleeting topic" |
| 325 | LeCroy (2012) (ID:72018612) | -EXCLUDE on "non-primary research" |
| 326 | Lee (2013) (ID:72018395) | -EXCLUDE on "dissertation & conference abstracts" |
| 327 | Lee (2015) (ID:72013532) | -EXCLUDE on "did not focus on loneliness experience" |
| 328 | Leendertse (2023) (ID:92340221) | -EXCLUDE on "loneliness as a fleeting topic" |
| 329 | Lencucha (2008) (ID:72014658) | -EXCLUDE on "did not focus on loneliness experience" |
| 330 | Lerbaek (2021) (ID:72011933) | -EXCLUDE on "did not focus on loneliness experience" *The topics discussed in this paper aren't really related to loneliness but rather just a description of how the participants deal with physical health problems. Even when the mechanism of "retreating" as a way to self management is mentioned it is only described in the context of physical symptoms and not related to schizophrenia.* |
| 331 | Leutwyler (2009) (ID:72019020) | -EXCLUDE on "did not focus on loneliness experience" *Related concepts were included but not subjective experience of loneliness.* |
| 332 | Leutwyler (2010) (ID:72016188) | -EXCLUDE on "did not focus on loneliness experience" |
| 333 | Li (2023) (ID:92340204) | -EXCLUDE on "solely using a quantitative method" |
| 334 | Lim (2020) (ID:72012546) | -EXCLUDE on "loneliness as a fleeting topic" |
| 335 | Lin (2006) (ID:72014757) | -EXCLUDE on "did not focus on loneliness experience" |
| 336 | Lindberg (2019) (ID:72012454) | -EXCLUDE on "did not include population with psychosis" |
| 337 | Liparini (2011) (ID:72018732) | -EXCLUDE on "dissertation & conference abstracts" |
| 338 | Liu (2012) (ID:72014223) | -EXCLUDE on "did not focus on loneliness experience" *None of the themes mentioned in results focus on experience of loneliness* |
| 339 | Lloyd (2004) (ID:72019589) | -EXCLUDE on "did not focus on loneliness experience" |
| 340 | Lloyd (2005) (ID:72014865) | -EXCLUDE on "did not focus on loneliness experience" |
| 341 | Lloyd (2017) (ID:72013113) | -EXCLUDE on "did not focus on loneliness experience" |
| 342 | Lu (2016) (ID:72083537) | -EXCLUDE on "did not meet the 50% threshold of psychosis sample" |
| 343 | Ludwig (2022) (ID:72017287) | -EXCLUDE on "dissertation & conference abstracts" |
| 344 | Luzius-Vanin (2023) (ID:92340055) | -EXCLUDE on "dissertation & conference abstracts" |
| 345 | Lyons (2022) (ID:72011779) | -EXCLUDE on "did not focus on loneliness experience" |
| 346 | Ma (2005) (ID:72014835) | -EXCLUDE on "did not focus on loneliness experience" |
| 347 | Mall (2017) (ID:72012993) | -EXCLUDE on "did not meet the 50% threshold of psychosis sample" |
| 348 | Mancini (2005) (ID:72014840) | -EXCLUDE on "did not meet the 50% threshold of psychosis sample"  *It is not specified what proportion if the participants have a psychosis diagnosis.* |
| 349 | Manuel (2012) (ID:72081628) | -EXCLUDE on "did not meet the 50% threshold of psychosis sample" |
| 350 | Martin (2011) (ID:72016133) | -EXCLUDE on "did not focus on loneliness experience" |
| 351 | Mathias (2015) (ID:72082682) | -EXCLUDE on "did not focus on loneliness experience" |
| 352 | May (2014) (ID:72018341) | -EXCLUDE on "did not meet the 50% threshold of psychosis sample" |
| 353 | Mayers (2010) (ID:72014372) | -EXCLUDE on "did not meet the 50% threshold of psychosis sample" |
| 354 | Mbuthia (2018) (ID:72083179) | -EXCLUDE on "did not meet the 50% threshold of psychosis sample" |
| 355 | McCann (2000) (ID:72016691) | -EXCLUDE on "did not focus on loneliness experience" |
| 356 | McCann (2004) (ID:72014904) | -EXCLUDE on "did not focus on loneliness experience" |
| 357 | McCann (2010) (ID:72016198) | -EXCLUDE on "did not focus on loneliness experience" |
| 358 | McCorkle (2009) (ID:72016264) | -EXCLUDE on "did not meet the 50% threshold of psychosis sample"  *It is not specified what proportion if the participants have a psychosis diagnosis.* |
| 359 | Mccoy (1997) (ID:72019908) | -EXCLUDE on "dissertation & conference abstracts" |
| 360 | McGranahan (2021) (ID:72082803) | -EXCLUDE on "did not focus on loneliness experience" |
| 361 | McGuire (2020) (ID:72017381) | -EXCLUDE on "did not focus on loneliness experience" |
| 362 | McNeely (2023) (ID:92340064) | -EXCLUDE on "did not focus on loneliness experience" |
| 363 | Meesters (2019) (ID:72012339) | -EXCLUDE on "did not focus on loneliness experience" |
| 364 | Melamed (2019) (ID:72012539) | -EXCLUDE on "did not focus on loneliness experience" |
| 365 | Mezzina (2006) (ID:72014775) | -EXCLUDE on "did not meet the 50% threshold of psychosis sample" |
| 366 | Mishara (2013) (ID:72013941) | -EXCLUDE on "dissertation & conference abstracts" |
| 367 | Mitchell (2022) (ID:72011741) | -EXCLUDE on "did not include population with psychosis" |
| 368 | Mizock (2014) (ID:72015913) | -EXCLUDE on "did not meet the 50% threshold of psychosis sample" |
| 369 | Moll (2009) (ID:72016239) | -EXCLUDE on "did not focus on loneliness experience" |
| 370 | Moltke (2014) (ID:72013715) | -EXCLUDE on "dissertation & conference abstracts" |
| 371 | Montross (2014) (ID:72013789) | -EXCLUDE on "did not meet the 50% threshold of psychosis sample" |
| 372 | Mora-Rios (2016) (ID:72015819) | -EXCLUDE on "did not focus on loneliness experience" |
| 373 | Morant (2011) (ID:72014270) | -EXCLUDE on "did not focus on loneliness experience" |
| 374 | Moses (2015) (ID:72083615) | -EXCLUDE on "did not meet the 50% threshold of psychosis sample" |
| 375 | Mulfinger (2019) (ID:72012679) | -EXCLUDE on "did not meet the 50% threshold of psychosis sample" |
| 376 | Murphy (2015) (ID:72013429) | -EXCLUDE on "did not focus on loneliness experience" |
| 377 | Mushkin (2018) (ID:72012780) | -EXCLUDE on "did not focus on loneliness experience" *Belongingness was explored but not loneliness* |
| 378 | Myers (2023) (ID:92340044) | -EXCLUDE on "dissertation & conference abstracts" |
| 379 | Narita (2017) (ID:72013062) | -EXCLUDE on "solely using a quantitative method" |
| 380 | Ng (2008) (ID:72014640) | -EXCLUDE on "did not focus on loneliness experience" *Although some of the themes "categories" were mildly related to the topic of loneliness but they weren't really focused on the loneliness experience but rather a general description of the patients thoughts about social relations and not specifically their own.* |
| 381 | Ng (2012) (ID:72014068) | -EXCLUDE on "did not focus on loneliness experience" |
| 382 | Nguyen (2022) (ID:92340419) | -EXCLUDE on "loneliness as a fleeting topic" |
| 383 | Nirmala (2020) (ID:72083545) | -EXCLUDE on "did not meet the 50% threshold of psychosis sample" |
| 384 | Nithsdale (2008) (ID:72014626) | -EXCLUDE on "did not focus on loneliness experience" |
| 385 | Noh (2008) (ID:72014584) | -EXCLUDE on "loneliness as a fleeting topic" |
| 386 | Noiseux (2008) (ID:72014591) | -EXCLUDE on "did not meet the 50% threshold of psychosis sample" |
| 387 | Noiseux (2010) (ID:72082670) | -EXCLUDE on "did not meet the 50% threshold of psychosis sample" |
| 388 | Nordstrom (2009) (ID:72014405) | -EXCLUDE on "did not meet the 50% threshold of psychosis sample" |
| 389 | Nxumalo (2019) (ID:72015542) | -EXCLUDE on "did not focus on loneliness experience" |
| 390 | Oliveira (2021) (ID:72011735) | -EXCLUDE on "did not include population with psychosis" |
| 391 | Oloyede (2023) (ID:92340875) | -EXCLUDE on "did not focus on loneliness experience" |
| 392 | Ostman (2013) (ID:72016012) | -EXCLUDE on "did not focus on loneliness experience" |
| 393 | Ostman (2014) (ID:72013687) | -EXCLUDE on "did not meet the 50% threshold of psychosis sample"  *55% of total sample (n=80) diagnosed with schizophrenia but only 20 interviewed and not sure how many of these met threshold?* |
| 394 | Palacios-Cena (2018) (ID:72017731) | -EXCLUDE on "did not meet the 50% threshold of psychosis sample" |
| 395 | Panczak (2016) (ID:72015757) | -EXCLUDE on "did not focus on loneliness experience" |
| 396 | Park (2017) (ID:72013120) | -EXCLUDE on "did not focus on loneliness experience" |
| 397 | Parry (2021) (ID:72012088) | -EXCLUDE on "did not include population with psychosis" |
| 398 | Parry (2021) (ID:72011909) | -EXCLUDE on "did not include population with psychosis" |
| 399 | Patel (2018) (ID:72012979) | -EXCLUDE on "did not focus on loneliness experience" |
| 400 | Pattison (2020) (ID:72012048) | -EXCLUDE on "single case studies" |
| 401 | Paul (2016) (ID:72017967) | -EXCLUDE on "loneliness as a fleeting topic" |
| 402 | Pedley (2018) (ID:72012861) | -EXCLUDE on "did not focus on loneliness experience" |
| 403 | Pentland (2003) (ID:72014968) | -EXCLUDE on "non-primary research" |
| 404 | Pereira (2005) (ID:72014737) | -EXCLUDE on "loneliness as a fleeting topic" |
| 405 | Perry (2009) (ID:72081665) | -EXCLUDE on "did not include population with psychosis" *It is not specified if any of the participants have a psychosis diagnosis.* |
| 406 | Pescosolido (1998) (ID:72015175) | -EXCLUDE on "did not meet the 50% threshold of psychosis sample" |
| 407 | Piat (2008) (ID:72016314) | -EXCLUDE on "did not meet the 50% threshold of psychosis sample" |
| 408 | Pilling (2017) (ID:72012987) | -EXCLUDE on "did not meet the 50% threshold of psychosis sample"  *Unsure how many of the participants diagnosed with schizophrenia or bipolar* |
| 409 | Pitt (2009) (ID:72014551) | -EXCLUDE on "did not focus on loneliness experience" |
| 410 | Pope (2019) (ID:72012764) | -EXCLUDE on "did not meet the 50% threshold of psychosis sample" |
| 411 | Poremski (2016) (ID:72013289) | -EXCLUDE on "did not meet the 50% threshold of psychosis sample"  *"We interviewed 20 service providers, 25 service users, and 9 caregivers."  service users made up less than 50%of the total number of participants.* |
| 412 | Potter (2015) (ID:72013448) | -EXCLUDE on "dissertation & conference abstracts" |
| 413 | Power (2010) (ID:72018887) | -EXCLUDE on "dissertation & conference abstracts" |
| 414 | Prout (2016) (ID:72013313) | -EXCLUDE on "did not focus on loneliness experience" |
| 415 | Provencher (2002) (ID:72015037) | -EXCLUDE on "did not meet the 50% threshold of psychosis sample" |
| 416 | Quin (2009) (ID:72014565) | -EXCLUDE on "did not meet the 50% threshold of psychosis sample" |
| 417 | Raghavan (2023) (ID:92341205) | -EXCLUDE on "did not meet the 50% threshold of psychosis sample" |
| 418 | Rai (2020) (ID:72082064) | -EXCLUDE on "did not meet the 50% threshold of psychosis sample" |
| 419 | Rambarran (2013) (ID:72014006) | -EXCLUDE on "did not focus on loneliness experience" |
| 420 | Ramon (2011) (ID:72014250) | -EXCLUDE on "did not meet the 50% threshold of psychosis sample"  *percentage of participants diagnosed with psychosis isn"t specified.* |
| 421 | Rapsey (2022) (ID:92340873) | -EXCLUDE on "did not focus on loneliness experience" |
| 422 | Redmond (2010) (ID:72014391) | -EXCLUDE on "did not focus on loneliness experience" |
| 423 | Roberts (2013) (ID:72014004) | -EXCLUDE on "did not meet the 50% threshold of psychosis sample" |
| 424 | Robertson (2003) (ID:72014990) | -EXCLUDE on "did not focus on loneliness experience" |
| 425 | Roby (2018) (ID:72082907) | -EXCLUDE on "did not focus on loneliness experience" |
| 426 | Ronngren (2018) (ID:72081650) | -EXCLUDE on "did not meet the 50% threshold of psychosis sample" |
| 427 | Roos (2016) (ID:72013288) | -EXCLUDE on "did not meet the 50% threshold of psychosis sample"  *The percentage of participants with a psychosis diagnosis isn't specified.* |
| 428 | Royce-Davis (2001) (ID:72016649) | -EXCLUDE on "did not focus on loneliness experience" *There wasn't any real focus on the subjective experience of participants diagnosed with schizophrenia.* |
| 429 | Rubio (2015) (ID:72013482) | -EXCLUDE on "dissertation & conference abstracts" |
| 430 | Rufato (2023) (ID:92339946) | -EXCLUDE on "did not focus on loneliness experience" |
| 431 | Rujkorakarn (2018) (ID:72012978) | -EXCLUDE on "did not focus on loneliness experience" |
| 432 | Ryan (2014) (ID:72018260) | -EXCLUDE on "did not meet the 50% threshold of psychosis sample" |
| 433 | Sangeorzan (2019) (ID:72012611) | -EXCLUDE on "did not meet the 50% threshold of psychosis sample"  *13 out of the 28 participants were had a psychosis diagnosis.* |
| 434 | Sariah (2014) (ID:72013646) | -EXCLUDE on "did not focus on loneliness experience" *There wasn't any real focus on the loneliness experience. even when social support and social network were mentioned there wasn't any description of a discrepancy between the actual social relations and the desired ones.* |
| 435 | Sathiyaseelan (2019) (ID:72012661) | -EXCLUDE on "did not include population with psychosis" |
| 436 | Schon (2009) (ID:72014535) | -EXCLUDE on "did not meet the 50% threshold of psychosis sample" |
| 437 | Secker (2002) (ID:72082349) | -EXCLUDE on "did not meet the 50% threshold of psychosis sample"  *Even though majority of participants have schizophrenia, results section does not differentiate data between the various other diagnoses.* |
| 438 | Shepherd (2012) (ID:72014063) | -EXCLUDE on "did not focus on loneliness experience" |
| 439 | Sheridan (2018) (ID:72081976) | -EXCLUDE on "did not focus on loneliness experience" |
| 440 | Shulman (2018) (ID:72012918) | -EXCLUDE on "dissertation & conference abstracts" |
| 441 | Sikira (2022) (ID:92340906) | -EXCLUDE on "did not focus on loneliness experience" |
| 442 | Simmons (2021) (ID:72012208) | -EXCLUDE on "dissertation & conference abstracts" |
| 443 | Simonsen (2023) (ID:92340054) | -EXCLUDE on "dissertation & conference abstracts" |
| 444 | Singh (2016) (ID:72013192) | -EXCLUDE on "did not meet the 50% threshold of psychosis sample" |
| 445 | Skjærpe (2023) (ID:92341034) | -EXCLUDE on "loneliness as a fleeting topic" |
| 446 | Skodlar (2008) (ID:72014627) | -EXCLUDE on "did not focus on loneliness experience" |
| 447 | Slasor (2005) (ID:72019493) | -EXCLUDE on "dissertation & conference abstracts" |
| 448 | Small (1984) (ID:72020256) | -EXCLUDE on "solely using a quantitative method" |
| 449 | Smartt (2021) (ID:72012189) | -EXCLUDE on "did not focus on loneliness experience" *No illustration of discrepancy between desired and actual social relations.* |
| 450 | Social Isolation and... (Xanthopoulou) (ID:72081655) | -EXCLUDE on "did not meet the 50% threshold of psychosis sample" |
| 451 | Spaniol (2002) (ID:72015056) | -EXCLUDE on "did not focus on loneliness experience" |
| 452 | Spies (2017) (ID:72017799) | -EXCLUDE on "did not include population with psychosis" |
| 453 | Spoorthy (2018) (ID:72012941) | -EXCLUDE on "did not focus on loneliness experience" |
| 454 | Stanton (2005) (ID:72016490) | -EXCLUDE on "did not focus on loneliness experience" |
| 455 | Stefanidou (2021) (ID:72012262) | -EXCLUDE on "did not include population with psychosis" |
| 456 | Stein (2001) (ID:72083460) | -EXCLUDE on "did not meet the 50% threshold of psychosis sample" |
| 457 | Stein (2013) (ID:72014020) | -EXCLUDE on "solely using a quantitative method" |
| 458 | Stopa (2013) (ID:72016027) | -EXCLUDE on "did not focus on loneliness experience" |
| 459 | Subandi (2022) (ID:92341231) | -EXCLUDE on "did not focus on loneliness experience" |
| 460 | Sun (2023) (ID:92340066) | -EXCLUDE on "did not meet the 50% threshold of psychosis sample" |
| 461 | Tanaka (2018) (ID:72082600) | -EXCLUDE on "did not focus on loneliness experience" |
| 462 | Tarko (2003) (ID:72019669) | -EXCLUDE on "dissertation & conference abstracts" |
| 463 | Tee (2022) (ID:72011848) | -EXCLUDE on "solely using a quantitative method" |
| 464 | Teferra (2013) (ID:72013852) | -EXCLUDE on "did not meet the 50% threshold of psychosis sample"  *Participants diagnosed with schizophrenia made up less than 50% of the total number of participants (24 out 51).* |
| 465 | Tennant (2023) (ID:92340059) | -EXCLUDE on "dissertation & conference abstracts" |
| 466 | Thomas (2019) (ID:72012593) | -EXCLUDE on "did not meet the 50% threshold of psychosis sample" |
| 467 | Thompson (2014) (ID:72013598) | -EXCLUDE on "did not focus on loneliness experience" |
| 468 | Tjornstrand (2020) (ID:72083349) | -EXCLUDE on "did not meet the 50% threshold of psychosis sample" |
| 469 | Topor (2014) (ID:72018288) | -EXCLUDE on "did not focus on loneliness experience" |
| 470 | Topor (2016) (ID:72081676) | -EXCLUDE on "did not meet the 50% threshold of psychosis sample"  *It is not mentioned what severe mental illness the participants have.* |
| 471 | Turner (2023) (ID:92340109) | -EXCLUDE on "did not focus on loneliness experience" |
| 472 | Twyman (2019) (ID:72081636) | -EXCLUDE on "did not meet the 50% threshold of psychosis sample" |
| 473 | Van Beek (2022) (ID:72011830) | -EXCLUDE on "did not focus on loneliness experience" *Only one of the themes was related to topics of loneliness and social support. However, it barely mentioned the subjective experience of the participants with a psychosis diagnosis.* |
| 474 | Walton (2000) (ID:72015010) | -EXCLUDE on "did not focus on loneliness experience" *Isolation is talked about in results but not loneliness.* |
| 475 | Wan (2008) (ID:72016343) | -EXCLUDE on "did not include population with psychosis" |
| 476 | Weiss (1992) (ID:72020059) | -EXCLUDE on "did not focus on loneliness experience" |
| 477 | Wheeler (2018) (ID:72081766) | -EXCLUDE on "did not meet the 50% threshold of psychosis sample" |
| 478 | Windell (2013) (ID:72013996) | -EXCLUDE on "did not focus on loneliness experience" |
| 479 | Wong (2014) (ID:72082309) | -EXCLUDE on "did not meet the 50% threshold of psychosis sample" |
| 480 | Wong (2018) (ID:72012889) | -EXCLUDE on "did not focus on loneliness experience" |
| 481 | Wood (2013) (ID:72013994) | -EXCLUDE on "solely using a quantitative method" |
| 482 | Woodside (2008) (ID:72016298) | -EXCLUDE on "did not focus on loneliness experience" |
| 483 | Wright (2007) (ID:72082826) | -EXCLUDE on "did not focus on loneliness experience" |
| 484 | Yarborough (2016) (ID:72013323) | -EXCLUDE on "did not meet the 50% threshold of psychosis sample" |
| 485 | Yennari (2012) (ID:72018576) | -EXCLUDE on "dissertation & conference abstracts" |
| 486 | Zubi (2013) (ID:72013816) | -EXCLUDE on "did not focus on loneliness experience" |
