# Supplementary material for: The experience of loneliness among people with psychosis: Qualitative meta-synthesis
Source: PLoS One. 2024 Dec 31;19(12):e0315763. doi: 10.1371/journal.pone.0315763 (PMC11687762; doi:10.1371/journal.pone.0315763)
Supplement: S5 Appendix — (DOCX) [file pone.0315763.s005.docx]

**Appendix S5: Table presenting characteristics of eligible articles**

| Citation | Study setting | Study aims | Sample size (n), diagnosis | Participant characteristics (age, gender, ethnicity) | Data collection method | Data analysis method | Major themes | CASP assessment (score out of 20) |
| --- | --- | --- | --- | --- | --- | --- | --- | --- |
| **Andersson et al., 2015**  **Aloneness and loneliness - persons with severe mental illness and experiences of being alone** | Psychiatric sector organization in Stockholm, Sweden | To explore experiences of being alone: what factors seem to have an impact on becoming alone and how people with SMI experience being alone | n = 19  Diagnosed with psychosis (ICD-10 F20–F29) | Aged 20-69 years  10 females  9 males | Interviews | Grounded theory | 1. To deselect 2. Not wanting 3. To get deselected 4. Wanting but not getting 5. Never had | 13 |
| **Avieli et al., 2016**  **Aging With Schizophrenia: A Lifelong Experience of Multidimensional Losses and Suffering** | Community members identified by rehabilitation facilities, Israel | To explore the subjective experience of suffering in aging individuals with schizophrenia | n = 18  Diagnosed with schizophrenia | Aged 60-69 years  7 females  11 males | Semi-structured in-depth interviews | Thematic content analysis | 1. “They throw me out of society like garbage” − the suffering of social rejection 2. “I have a mother and I don’t have a mother” − the complexity of family relationships 3. “It’s like a wound in your head” – the torture of symptoms 4. “It makes me sleepy, weak and crazy” – suffering caused by the side effects of medication 5. “They just kill me” – suffering caused by cumulative hospitalizations 6. “I could have had all this” - suffering caused by loss of employment potential 7. “I wish I was free in my own home” – loss of independent accommodation 8. “It is hard for me to find friends” – loss of social life 9. “This is the greatest tragedy” − loss of intimate relationships and parenthood | 20 |
| **Barut et al., 2016**  **Sense of Belonging and Hope in the Lives of Persons with Schizophrenia** | Freestanding psychiatric hospital that is part of a tertiary academic medical center, United States | To explore sense of belonging and hope in the lived experience of people with chronic schizophrenia spectrum disorders, and to provide insight into patients’ perceptions of treatment for their mental illness | n = 20  Primary diagnosis of a chronic schizophrenia spectrum disorder | Aged 18-65 years  7 females  13 males  14 white  6 black | Semi-structured interviews | Not explicitly stated | 1. Sense of Belonging 2. Not belonging 3. Goal and purpose 4. Absence of hope 5. Treatment | 17 |
| **Blajeski, 2022**  **How early stigmatizing experiences, peer connections, and peer spaces influenced pathways to employment or education after a first-episode of psychosis** | Young Adult Leadership Council of the Early Assessment and Support Alliance, United States | To explore the pathways to employment, education, and/or disability among young adults with First-Episode Psychosis (FEP) with the intent to reduce poverty and disability among this population | n = 10  Lived experience of first-episode psychosis | Aged 21-28 years, mean age of 26 (SD = 1.60)  5 females  5 males  7 white  1 Mexican-American  1 Asian/Pacific Islander  1 Asian-American | Semi-structured interviews | Template analysis | 1. Hospitalization and Psychiatric Labeling Construct Stigma 2. “Yeah, I Know What That’s Like”: Connecting With Peers to Resolve Stigma 3. Employment Connection and Peer Mentoring Through the Young Adult Leadership Council | 19 |
| **Bögle & Boden et al., 2022**  **'It was like a lightning bolt hitting my world': Feeling shattered in a first crisis in psychos** | Mental health charities, United Kingdom | To gain an in-depth understanding of the lived, felt experiences of a first crisis in psychosis | n = 7  Recently experienced a first crisis in psychosis | Aged 15-70 years  2 females  4 males  1 transgender  6 white  1 black | Semi-structured interviews | Interpretative phenomenological analysis | 1. ‘It was like a lightning bolt hitting my world’: Feeling shattered 2. ‘That’s really strange’: An all-enveloping strangeness and lingering threat 3. ‘I was left stranded in the dark’: Lacking a sense of belonging | 18 |
| **Boydell et al., 2003**  **Interpreting narratives of motivation and**  **schizophrenia: A biopsychosocial understanding** | A self-help organization, a first-episode hospital clinic, and a participant's place of employment, Canada | To explore the subjective experience of motivation as revealed in the narratives of persons diagnosed with schizophrenia and the meanings individuals attached to this experience | n = 6  Diagnosed with schizophrenia | Ranging in age from the early twenties to the mid-forties  2 females  4 males | In-depth interviews | The seven stages of analysis as outlined in the work of Diekelmann (1992) | 1. Experiencing schizophrenia 2. Being on “meds” 3. Spirit making/breaking 4. Experiencing stigma 5. Adopting a personal stance toward the world | 15 |
| **Budziszewska et al., 2020**  **Love and Romantic Relationships in the Voices of Patients Who Experience Psychosis: An Interpretive Phenomenological Analysis** | Outpatient psychiatric clinic in Warsaw, Poland | To understand how patients, diagnosed with schizophrenia and with the experience of psychosis, approach and give meaning to love, especially in the context of romantic relationships | n = 10  Had experienced severe mental illness and diagnosed with schizophrenia | Mean age of 34.4 (SD = 6.07)  5 females  5 males | Semi-structured interviews | Interpretative phenomenological analysis | 1. Illness adjustment is all-consuming at first and can lead to isolation 2. Inner obstacles: illness-induced changes in experiencing love can be obstacles to entering and maintaining romantic relationships 3. External obstacles: low status, discrimination, and poverty make it difficult to establish and maintain close relationships 4. Experiencing sexuality becomes more challenging 5. Ways of coping | 18 |
| **Chernomas et al., 2000**  **Perspectives of women living with schizophrenia** | Community members recruited through advertisements and information sheets provided by health care professionals who work with this population, Canada | To investigate the perceptions of women with schizophrenia or schizoaffective disorder about their illness in the context of their life stages and corresponding health needs | n = 28  Self-identified as having schizophrenia or schizoaffective disorder  Schizophrenia = 18 Schizoaffective disorder = 10 | Aged 21-over 60 years  28 females | Focus group interviews | Thematic analysis | 1. Work 2. Stigma and rejection 3. Relationships and intimacy 4. Pregnancy and motherhood 5. Responsibility for illness 6. Physical health and interaction with a physician 7. Hope and spirituality | 13 |
| **Deland et al., 2011**  **A Phenomenological Analysis of the Psychotic Experience** | Psychiatric inpatient and outpatient services, Sweden | To trace out the meaning structure of the lived experience of psychosis | n = 6  Unspecified nonorganic psychosis = 4  Schizophrenia = 1  Postpartum psychosis = 1 | Aged 20-40 years  4 females  2 males | Semi-structured interviews | Phenomenological-psychological method | 1. The feeling of estrangement in relationship to the world 2. The dissolution of time 3. The loss of intuitive social knowledge 4. The alienation of oneself 5. The loss of intentionality/loss of agency | 15 |
| **DeNiro, 1995**  **Perceived alienation in individuals with residual-type schizophrenia** | Inpatient services, United States | To explore the experience of alienation from the perspective of a convenience sample of 20 individuals with residual-type schizophrenia | n = 20  Diagnosed with schizophrenia, with the fifth digit of the DSM-III-R classification coded as 2 (chronic) or 4 (chronic with acute exacerbation) | Mean age of 35.37  6 females  14 males | Semi-structured interviews | Content analysis | 1. Loneliness 2. Social isolation 3. Lack of solidarity or feelings of nonbelonging 4. Effect of psychiatric medication and treatment on alienation 5. Additional influences on alienation 6. Closeness and separation at specified age ranges | 10 |
| **Firmin et al., 2021**  **How psychosis interrupts the lives of women and men differently: a qualitative comparison** | All participants were initially recruited from a period spanning 2004 until 2016, for three separate studies, United States | To seek a better understanding of the potential effects of gender, particularly whether the kinds of disruptions psychosis bring to one’s sense of self and the world differ for men and  women | n = 53  Diagnosed with schizophrenia or schizoaffective disorder | Aged 26-59 years, mean age of 43 (SD = 8.8)  27 females  26 males  33 Black/African American  19 Caucasian  1 Hispanic | The Indiana Psychiatric Illness Interview (IPII) | Interpretative phenomenological analysis | 1. Parenting and loss of parenting roles 2. Work and loss or changes in work trajectories 3. Isolation and strain on interpersonal relationships 4. Manifestations of stigma | 18 |
| **Gajwani et al., 2016**  **"What is the point of life?": An interpretative phenomenological analysis of suicide in young men with first-episode psychosis** | Early Intervention Service (EIS) for psychosis, United Kingdom | To examine the personal meaning of their own suicide attempts for young men with a diagnosis of first-episode psychosis, and to explore young men’s experience of emerging psychosis and its relation to the suicide attempt, within the context of their life script | n = 7  Diagnosed with schizophrenia or related disorders (ICD-10 F20, 22, 23) and have made a suicide attempt in the last 24 months that has led to medical intervention | Aged 18-35 years, mean age of 22.85 (SD = 3.48)  7 males  2 British Asian/Asian Pakistani  1 British African Caribbean  4 White British | Semi-structured interviews | Interpretative phenomenological analysis | 1. Self-as-vulnerable (intra- and inter-personal relationships) 2. Appraisal of cumulative life events as unbearable 3. Meaning of recovery marked by shared sense of hope and imagery for the future | 17 |
| **Gee et al., 2003**  **Quality of life in schizophrenia: A grounded theory approach** | Local Community Mental Health Teams and an acute inpatient unit, United Kingdom | To elicit subjective reflections regarding the impact of schizophrenia on health related quality of life | n = 6  Diagnosed with schizophrenia | Aged 20-55 years, mean age of 33.3  3 females  3 males | Interviews | Grounded theory | 1. Barriers placed on interpersonal relationships 2. Reduced control of behaviours and actions 3. Loss of opportunity to fulfil occupational roles 4. Financial constraints on activities and plans 5. Subjective experience of psychotic symptoms 6. Side effects and attitudes to medication 7. Psychological responses to schizophrenia 8. Labelling and attitudes from others 9. Concerns for the future 10. Positive outcomes from experiences | 20 |
| **Gunnmo & Bergman, 2011**  **What do individuals with schizophrenia need to increase their well-being** | Outpatient ward for individuals with psychosis, Sweden | To deepen the knowledge of how individuals with schizophrenia themselves describe what they need in order to increase their well-being in everyday life | n = 7  Medical history of psychosis (all but one have been diagnosed with schizophrenia according to DSM-IV) | Aged 33-66 years  6 females  1 male | Interviews with the explorative approach, open and focused on the question: “What is important for you to get help with?” | Grounded theory | 1. Striving for a normal life  - Receiving information about the illness - Taking part in secure professional relationships - Taking part in social contacts - Having a meaningful employment - Receiving help with practical matters during psychotic relapse | 16 |
| **Hansen et al., 2020**  **"Needing different things from different people"-A qualitative exploration of recovery in first episode psychosis** | Early detection team for psychosis (TOPS) at Haukeland University Hospital, Norway | To explore how young adults with first episode psychosis experienced relationships inside and outside the mental healthcare services related to their early recovery processes | n = 10  Having psychotic symptoms, defined as a score of four or higher on any of the following PANSS items: P1 (delusions), P2 (disorganized thought), P3 (hallucinatory behaviour), P4 (excitement), P5 (grandiosity), P6 (suspiciousness), and G9 (unusual thought content) | Aged 19-32 years  7 females  3 males | In-depth semi-structured interviews | Thematic and reflexive team-based analytical methods | 1. Being seen as a unique person with preferences: “simply being met” 2. Getting help when you are lost: “A personal assistant” 3. Supportive family networks: “I know they are out there cheering for me” 4. They cannot understand everything: “after all, it’s my problem” 5. Different kinds of friendships: “friends with whom … I am just myself” | 18 |
| **Harris et al., 2019**  **Factors that contribute to psychological resilience to suicidal thoughts and behaviours in people with schizophrenia diagnoses: qualitative study** | National Health Service (for example community, early intervention and recovery mental health services, rehabilitation units) and self-help groups, United Kingdom | To investigate factors that contribute to psychological resilience to suicidal thoughts and behaviours from the perspectives of people with a diagnosis of schizophrenia | n = 20  Diagnosed with non-affective psychosis or schizophrenia diagnoses who had experience of suicide thoughts and behaviours  Schizophrenia = 5  Paranoid schizophrenia = 8  Chronic schizophrenia = 1  Treatment resistant schizophrenia = 1  Schizoaffective disorder = 2  Psychotic disorder = 1  Acute psychosis = 1  Unspecified non-organic psychosis = 1 | Aged 23-75 years, mean age of 48 (SD = 15.5)  10 females  10 males  16 White British  1 Black British  3 mixed ethnicity | Semi-structured interviews | Thematic analysis | 1. Understanding experiences 2. Active behaviours 3. Relationship dynamics | 17 |
| **Hogg et al., 2022**  **The nuances of "the social cure" for people who experience psychosis** | UK Early Intervention for Psychosis services (EIP), the UK branch of the charity The Hearing Voices Network (HVN), and a UK university | To explore the social identities of people  with psychosis, perceptions of how and why they have changed with  the advent of psychosis, and their relationship to wellbeing | n = 26  Psychotic experiences screened with Psychosis Screening Questionnaire PSQ (Bebbington & Nayani, 1995) | Aged 18-45 years, mean age of 27 (SD = 6.4)  12 females  14 males  22 White British  1 White other  1 Black Caribbean  1 Black African  1 Middle Eastern | Semi-structured interviews | Thematic analysis | 1. The development of psychosis as a turning point in social group memberships 2. Healing power of social connections 3. Identity compartmentalisation | 20 |
| **Huckle et al., 2021**  **Experiences of friendships of young people with first-episode psychosis: A qualitative study** | NHS early intervention service for psychosis in London, United Kingdom | To explore experiences of friendships of young people with first-episode psychosis, focusing  especially on any perceived changes in their friendships or approach to peer relationships  as a result of the illness | n = 14  Had first-episode psychosis | Aged 19-31 years  7 females  7 males  2 Asian  1 Black African  2 Black British  3 White British  6 White European | Semi-structured interviews | Thematic analysis | 1. Self stigma: Participant directed loss of social contact 2. Symptoms ended friendships as I knew them 3. Friendships incompatible with recovery 4. Getting better: What can friends do to help? 5. Assessment of current social situation 6. Making new friends 7. The role of services | 17 |
| **Humberstone, 2002**  **The experiences of people with schizophrenia living in supported accomodation: A qualitative study using grounded theory methodology** | Registered local community mental health accommodation with 24-hour staffing in South and Central Auckland, Australia | To develop a detailed analysis of the subjective experiences of people with schizophrenia living in highly staffed supported accommodation | n = 13  Diagnosed with schizophrenia | Age not stated  3 females  10 males | Semi-structured interviews | Grounded theory | 1. A way to survive 2. What had to be survived 3. Survival strategies | 15 |
| **Jenkins & Carpenter-Song, 2009**  **Awareness of stigma among persons with schizophrenia: marking the contexts of lived experience** | Community mental health outpatient facilities in a northeastern US metropolitan area that served Euro-American and African-American ethnic groups | To investigates the subjective experience of stigma attached to schizophrenia-related disorders | n = 90  81.1% participants had schizophrenia and 18.9% had schizoaffective disorder, assessed through the Structured Clinical Interview for DSM-IV | Mean age of 40.7 (SD = 7.9)  Females = 45.5%  Males = 54.4%  Euro-Americans = 77.8%  African-Americans = 22.2% | Semi-structured open-ended ethnographic interview | Grounded theory | 1. Social relations 2. Domains of identity | 15 |
| **Johnson & Montgomery, 1999**  **Chronic mentally ill individuals reentering the community after hospitalization. Phase II: the urban experience** | A large provincial psychiatric hospital located in an urban centre in Ontario, Canada | To examine the experiences of a group of chronic mentally ill individuals who were reentering the community after hospitalization. It focused on the expectations they had before discharge as well as the reality of being in the community after discharge | n = 8  Six of the participants were diagnosed with schizophrenia | Aged early 30s-mid-50s  3 females  5 males | Long interview (Moustakas, 1994) | A process described by Giorgi (1985), which involves multiple readings of the interviews to uncover commonalities | Three themes (authors did not title the themes)  The first theme reflects the notion that ties to the hospital remained strong after discharge, and that hospitalization  and living in the community are phases of a recurring pattern  The second theme refers to the interaction between symptoms of illness and personal circumstances, each exacerbating the other  The third theme refers to the participants’ goals and the barriers to achieving their goals | 9 |
| **Knight et al., 2023**  **It just feels like an invasion': Black first-episode psychosis patients' experiences with coercive intervention and its influence on help-seeking behaviours** | The First Episode Psychosis Program (FEPP), Canada | To (a) explore their experiences with coercive interventions and (b) describe how these experiences may have influenced help-seeking behaviours | n = 5  Had first-episode psychosis | Aged 19-38 years  5 males  5 black | Semi-structured interviews | Interpretative phenomenological analysis | 1. Sense of confusion 2. Forced medication 3. Feelings of loneliness 4. Police contact 5. Discrimination and prejudice 6. Feeling unheard 7. Mistrust 8. Powerlessness | 20 |
| **Ko et al., 2022**  **Re-figuration of suffering: Transitional phenomena of people living with schizophrenia in group analysis** | Psychiatric rehabilitation ward of a teaching hospital in Southern Taiwan | To examine how people living with schizophrenia recognise their lived experiences in free-floating group discussions | n = 14  Had a schizophrenia diagnosis with alleviated psychiatric symptoms | Aged 33-61 years  7 females  7 males | Free-floating group discussions | Interpretative phenomenological analysis | 1. Being treated as an object (others and me as an object) 2. Encountering the self through others (me and others) 3. Returning to the self (others, me, and myself) | 19 |
| **Ludwig et al., 2022**  **Lived experience of loneliness in psychosis: A qualitative approach** | Schizophrenia Treatment and Evaluation Program (STEP) community outpatient clinics in North Carolina, United States | To explore loneliness among persons with schizophrenia spectrum disorders, and to examine which aspects of living with psychosis impact perceptions of loneliness, including perceived social support and symptoms | n = 16  Schizophrenia = 7  Schizoaffective = 9 | Mean age of 39.0 (SD = 7.15)  6 females  10 males  12 Caucasian  3 African American  1 other | Semi-structured interviews | Thematic analysis | 1. Physical Barriers to Adequate Social Engagement or Community Involvement 2. Patterns of Social Contact that Contribute to Lonely Feeling 3. Psychological Variables 4. Coping Strategies to Manage Loneliness | 16 |
| **Macdonald et al., 2005**  **What happens to social relationships in early psychosis? A phenomenological study of young people's experiences** | Recovery Group Program (see Albiston et al., 1998) at the Early Psychosis Prevention and Intervention Centre (EPPIC) in metropolitan Melbourne, Australia | To explore young people’s experiences of social relationships during the recovery phase of first-episode psychosis in order to facilitate their social relations | n = 6  Had first-episode psychosis | Aged 19.50-25.25 years, mean age of 21.99  1 female  5 males | In-depth interviews | Colaizzi’s (1978) and Moustakas’  (1994) accounts of phenomenological analysis | 1. Hanging out with people I like and who understand me 2. Valuing families and other supports 3. Spending less time with old friends 4. Something happened to me - being different now 5. Building new relationships | 19 |
| **Mauritz & Meijel, 2009**  **Loss and Grief in Patients With Schizophrenia: On Living in Another World** | University hospital, Amsterdam, Netherlands | To address the lived experience of grief in schizophrenia | n = 10  Diagnosed with schizophrenia according to the Diagnostic and Statistical Manual of Mental Disorders, Fourth Edition | Aged 21-38 years, mean age of 26.6  1 female  9 males | Semi-structured interviews | Grounded theory | 1. Internal Loss: Living in a Different World 2. External Loss: Not Belonging 3. Experiences of Grief 4. Coming to Terms | 18 |
| **Mawson et al., 2011**  **Voice hearing within the context of hearers’ social worlds: An interpretative phenomenological analysis** | NHS mental health services in North West England, United Kingdom | To provide a deeper understanding of the interpersonal context of voice hearing by exploring participants’ relationships with their voices and other people in their lives | n = 10  Schizophrenia = 8  Schizoaffective disorder = 1  Paranoid Schizophrenia = 1 | Aged 26-51 years | Semi-structured interviews | Interpretative phenomenological analysis | 1. Person and voice: Similar identities, varied relationships 2. Voices confirming and changing relationship with the self 3. A ‘Battle’ for control 4. Friendships facilitating the ability to cope 5. ‘Sometimes I do think there is a bit of a boundary’: Voices creating distance in social relationships | 20 |
| **Nilsson et al., 2007**  **The tune of want in the loneliness melody - Loneliness experienced by people with serious mental suffering** | The participants’ home, Norway | To present the results of a study of the significance of loneliness in patients with serious psychiatric disease, who live alone at home | n = 8  Diagnosed with schizophrenia | Aged 20-50 years  Gender not stated | Qualitative participatory observation, ethnographical approach and conversational research interview | Hermeneutic interpretation | 1. The experience of loneliness at a doing level 2. The experience of loneliness at an existential level 3. The experience of loneliness at an ontological level 4. What relieves the informants’ feeling of loneliness? | 16 |
| **Nilsson et al., 2019**  **Social Life in the Schizophrenia Spectrum:**  **A Phenomenological Study of Five Patients** | A psychiatric facility in Copenhagen, Denmark | To explore a less ill subgroup of schizophrenia spectrum patients’ ways of navigating the social world and examine potential links to anomalous self-experiences by applying key insights from phenomenology and anthropology | n = 5  Schizophrenia = 4  Schizotypal disorder = 1 | Aged 24-52 years  4 females  1 male | Semi-structured interviews | Thematic analysis laid out by Braun and Clarke (2006) | 1. Social detachment 2. Void time 3. The importance of particular activities or circumstances 4. The organizing function of social engagement 5. The Positive effect of clearly defined roles 6. Positive withdrawal | 15 |
| **Ogden, 2014**  **Interpersonal Relationship Narratives of Older Adults With Schizophrenia-Spectrum Diagnoses** | Three sample sites: (1) a senior residence for older adults with serious mental illnesses, (2) a day treatment program run by a larger nonprofit organization that operated housing programs for persons with serious mental illnesses, and (3) an inpatient psychiatric unit, United States | To explore how older adults with ongoing symptoms of schizophrenia-spectrum diagnoses understood and expressed their life stories in the face of life course and present-time adversities and resilience, focusing on narratives of interpersonal relationships | n = 7  Had a primary diagnosis of schizophrenia with no constraints on secondary or additional diagnoses | Aged 56-73 years, mean age of 66.3  3 females  4 males  3 white  4 black | Life history calendars and time diaries, interviews, and field observations | Thematic narrative analysis | 1. Relational losses 2. Relational voids 3. Need for solitude 4. Relational adjustments 5. Relational adaptations. | 20 |
| **Rose et al., 2011**  **Reported stigma and discrimination by people with a diagnosis of schizophrenia** | 15 sites representing the International Discrimination and Stigma Outcomes (INDIGO) project (including ‘old’ EU countries, newly acceded countries, candidate countries for the EU and countries outside the EU) | To examine the extent of stigma and discrimination as reported by people with a diagnosis of schizophrenia | n = 75  Diagnosed with schizophrenia | Aged 19-51 years  30 females  45 males | Interviews with the Discrimination and Stigma Scale v10 | Thematic analysis | 1. Shunned 2. Social withdrawal 3. Entitlements because of mental illness | 16 |
| **Shin & Joung, 2023**  **COVID-19 coping experiences of individuals with schizophrenia: A grounded theory approach in Korea** | A mental rehabilitation facility and the online community for self-help groups, Korea | To explore  the experiences of individuals with schizophrenia coping with COVID-19 | n = 17  Diagnosed with schizophrenia spectrum disorder | Aged 21-50 years  9 females  8 males | Interviews | Grounded theory | 1. Disparity in care 2. Adaptation through tailored care 3. Return to regular lives | 20 |
| **Sheaves et al., 2021**  **The challenges and opportunities of social connection when hearing derogatory and threatening voices: A thematic analysis with patients experiencing psychosis** | Clinical teams within Oxford Health NHS Foundation Trust, United Kingdom | To learn about the patient experience of being around other people when hearing derogatory and threatening voices (DTVs) | n = 15  Schizophrenia = 10 Schizoaffective disorder = 1  Psychosis NOS = 4 | Aged ≤20-60 years  Gender not stated  13 White British  1 Chinese  1 Black British | Semi-structured interviews | Thematic analysis | 1. Reasons why interacting with people is difficult when hearing DTVs 2. The relationship between social connection and DTVs 3. Factors which enable voice hearers to connect with others | 19 |
| **Strand et al., 2015**  **'I divide life into different dimensions, one mental and one physical, to be able to handle life, you know?' Subjective accounts of the content of psychotic symptoms** | Psychiatric outpatient unit specializing in psychosis, located on the outskirts of an urban area in the south of Sweden | To provide a deeper understanding of how individuals with psychosis make sense of the content of their psychotic symptoms | n = 12  Diagnosed with psychosis (9 participants were experiencing active symptoms, and 3 other participants had experienced symptoms within the last 12 months) | Aged 29-63 years  5 females  7 males | Semi-structured interviews | Interpretive phenomenological analyses | 1. Grandiose content 2. Harassing content 3. Commanding content 4. Supportive content | 16 |
| **Sung et al., 2006**  **Comparing life experiences of college students with differing courses of schizophrenia in Korea: Case studies** | University hospitals in Korea | To identify how daily experiences of college students with schizophrenia reflected the course of their disease | n = 8  Diagnosed with schizophrenia | Aged 21-26 years  3 females  5 males | In-depth interviews | Qualitative content analysis and in-depth analysis of case studies | 1. Recovering course 2. Deteriorating course 3. Fluctuating course | 17 |
| **Sung & Puskar, 2006**  **Schizophrenia in college students in Korea: A qualitative perspective** | University hospitals in Korea | To identify the salient themes that characterize the life experiences of college students with schizophrenia | n = 21  Diagnosed with schizophrenia according to DSM-IV | Aged 20-27 years, mean age of 23.1  8 females  13 males | Semi-structured interviews | Qualitative content analysis | 1. Experiences involving family interactions 2. Experiences involving interactions with friends 3. Experiences of school life 4. Experiences of everyday life 5. Experiences of social role performance 6. Experiences of a mental illness | 18 |
| **Tan et al., 2014**  **Distress, trauma, and recovery: adjustment to first episode psychosis** | Early Intervention Service in the West Midlands, United Kingdom | To understand the experiences that people with early psychosis are adjusting to and their perceived barriers to recovery | n = 8  Had first-episode psychosis | Aged 22-26 years, mean age of 24.25 (SD = 1.16)  2 females  6 males  8 White British | Semi-structured interviews | Grounded theory | 1. The distress of life experiences 2. Psychological distress 3. Distress due to systems | 20 |
| **White et al., 2021**  **"Sex isn't everything": views of people with experience of psychosis on intimate relationships and implications for mental health services** | Community mental health services in the North West of England, United Kingdom | To investigate how people who experience psychosis conceptualise romantic relationships, and to identify whether/how people with experience of psychosis would like community mental health services to provide support with romantic relationship issues | n = 10  Had either a diagnosis of psychosis or experience of psychosis that met the criteria for acceptance into early intervention services | Aged 21-64 years  4 females  6 males  7 White British/English /Irish  2 White British with mixed heritage (Polish and Afro-American)  1 French Jewish | Semi-structured interviews | Reflexive thematic analysis | 1. Conceptualising romantic relationships: ‘Sex isn’t everything’ 2. Stigma and discrimination: ‘… they don’t give you a chance if you’re mentally ill’ 3. Support from mental health services: ‘I’d have to trust them’ | 19 |
| **Williams & Collins, 2002**  **The social construction of disability in schizophrenia** | The Clarke Site of the Centre for Addiction and Mental Health in Toronto, Canada | To explore the potential for social construction of disability through secondary analysis of qualitative data | n = 15  Diagnosed with schizophrenia | Aged 21-47 years, mean age of 29.6  2 females  13 males  8 Canadian  4 European  2 African  1 Caribbean | Secondary analysis of qualitative data collected by Williams & Collins (1999) | A modified editing approach to analysis, in which the researcher systematically reduces and reassembles data (McCracken, 1988; Glaser & Strauss, 1967) | 1. The Construction of Disconnection: “It Made Me Feel Alienated From All My Friends” 2. The Construction of Dependence: “I Feel Like I’m Going to Be Dependent All My Life” 3. The Construction of Devaluation: “Why Isn’t Society Looking After Them?” 4. The Construction of Dysfunction: “I Lost Everything I Had” 5. Resistance | 14 |
| **Yen et al., 2020**  **The self-stigmatization of patients with schizophrenia: A phenomenological study** | Outpatient clinic, Taiwan | To explore the self-stigma of patients with schizophrenia | n = 15  Diagnosed with schizophrenia | Aged 25-62 years  6 females  9 males | Unstructured interviews | Colaizzi's seven-step method (1978) | 1. Experience of the origin of the self-stigma 2. Suffering experience of self-stigma 3. Experience of coping with self-stigma | 20 |
| **Zheng & Zhang, 2022**  **“Why I stay in community psychiatric**  **rehabilitation": a semi-structured survey**  **in persons with schizophrenia** | 6 community psychiatric rehabilitation centers of Beijing  Haidian district, China | To explore the underlying causes of patients with Schizophrenia in China staying in rehabilitation centers for longer periods of time and subsequently failing  to integrate, and to provide valuable  insights to guide effective and applicable rehabilitation  policies | n = 28  Diagnosed with Schizophrenia | Aged 35-67 years, mean age of 50.5  17 females  11 males | Semi-structured interviews | Thematic analysis | 1. Rehabilitation effect 2. Attractiveness 3. Difficulties in social communication | 16 |

CASP (Critical Appraisal Skills Programme): a score of 2 denotes that criterion is fully met, a score of 1 denotes that criterion is partially met, a score of 0 denotes that criterion is not met

SD: Standard deviation
