# Supplementary material for: The experience of loneliness among people with psychosis: Qualitative meta-synthesis
Source: PLoS One. 2024 Dec 31;19(12):e0315763. doi: 10.1371/journal.pone.0315763 (PMC11687762; doi:10.1371/journal.pone.0315763)
Supplement: S6 Appendix — (DOCX) [file pone.0315763.s006.docx]

**Appendix S6: Evaluation of study quality according to the Critical Appraisal Skill Programme (CASP) Qualitative Checklist**

| Study name & date | CASP Quality Assessment Score Categories | | | | | | | | | | Total score |
| --- | --- | --- | --- | --- | --- | --- | --- | --- | --- | --- | --- |
|  | Validity | | | | | | Results | | | Value of research |  |
|  | Clear aims | Appropriate qualitative methodology | Appropriate research design | Appropriate recruitment strategy | Appropriate data collection | Consideration for reflexivity | Consideration of ethical issues | Sufficiently rigorous data analysis | Clear statement of findings |  |  |
| **Andersson et al., 2015** | 2 | 2 | 1 | 2 | 1 | 0 | 1 | 1 | 2 | 1 | 13 |
| **Avieli et al., 2016** | 2 | 2 | 2 | 2 | 2 | 2 | 2 | 2 | 2 | 2 | 20 |
| **Barut et al., 2016** | 2 | 2 | 2 | 2 | 2 | 0 | 2 | 1 | 2 | 2 | 17 |
| **Blajeski et al., 2022** | 2 | 2 | 2 | 2 | 1 | 2 | 2 | 2 | 2 | 2 | 19 |
| **Bögle & Boden, 2022** | 2 | 2 | 2 | 2 | 2 | 2 | 1 | 2 | 1 | 2 | 18 |
| **Boydell et al., 2003** | 2 | 2 | 1 | 2 | 2 | 0 | 2 | 1 | 2 | 1 | 15 |
| **Budziszewska et al., 2020** | 2 | 2 | 2 | 2 | 1 | 2 | 2 | 2 | 2 | 1 | 18 |
| **Chernomas et al., 2000** | 2 | 2 | 2 | 1 | 1 | 0 | 1 | 1 | 1 | 2 | 13 |
| **Deland et al., 2011** | 2 | 2 | 2 | 2 | 1 | 2 | 2 | 0 | 1 | 1 | 15 |
| **DeNiro, 1995** | 2 | 2 | 0 | 1 | 2 | 0 | 0 | 1 | 1 | 1 | 10 |
| **Firmin et al., 2021** | 2 | 2 | 2 | 1 | 2 | 2 | 1 | 2 | 2 | 2 | 18 |
| **Gajwani et al., 2016** | 2 | 2 | 2 | 2 | 2 | 0 | 2 | 2 | 2 | 1 | 17 |
| **Gee et al., 2003** | 2 | 2 | 2 | 2 | 2 | 2 | 2 | 2 | 2 | 2 | 20 |
| **Gunnmo & Bergman, 2011** | 2 | 2 | 2 | 2 | 2 | 0 | 2 | 2 | 1 | 1 | 16 |
| **Hansen et al., 2020** | 2 | 2 | 2 | 2 | 2 | 1 | 2 | 2 | 2 | 1 | 18 |
| **Harris et al., 2019** | 2 | 2 | 2 | 2 | 2 | 0 | 1 | 2 | 2 | 2 | 17 |
| **Hogg et al., 2022** | 2 | 2 | 2 | 2 | 2 | 2 | 2 | 2 | 2 | 2 | 20 |
| **Huckle et al., 2021** | 2 | 2 | 2 | 2 | 2 | 0 | 1 | 2 | 2 | 2 | 17 |
| **Humberstone, 2002** | 2 | 2 | 2 | 2 | 2 | 0 | 0 | 2 | 2 | 1 | 15 |
| **Jenkins & Carpenter-Song, 2009** | 2 | 2 | 2 | 2 | 2 | 0 | 0 | 2 | 2 | 1 | 15 |
| **Johnson & Montgomery, 1999** | 2 | 2 | 0 | 0 | 1 | 0 | 0 | 1 | 2 | 1 | 9 |
| **Knight et al., 2023** | 2 | 2 | 2 | 2 | 2 | 2 | 2 | 2 | 2 | 2 | 20 |
| **Ko et al., 2022** | 2 | 2 | 2 | 2 | 2 | 2 | 2 | 2 | 2 | 1 | 19 |
| **Ludwig et al., 2022** | 2 | 2 | 2 | 2 | 2 | 0 | 1 | 2 | 2 | 1 | 16 |
| **Macdonald et al., 2005** | 2 | 2 | 2 | 2 | 2 | 2 | 1 | 2 | 2 | 2 | 19 |
| **Mauritz & Meijel, 2009** | 2 | 2 | 2 | 1 | 2 | 2 | 1 | 2 | 2 | 2 | 18 |
| **Mawson et al., 2011** | 2 | 2 | 2 | 2 | 2 | 2 | 2 | 2 | 2 | 2 | 20 |
| **Nilsson et al., 2008** | 2 | 2 | 2 | 1 | 2 | 0 | 2 | 2 | 2 | 1 | 16 |
| **Nilsson et al., 2019** | 2 | 2 | 1 | 1 | 2 | 0 | 1 | 2 | 2 | 2 | 15 |
| **Ogden, 2014** | 2 | 2 | 2 | 2 | 2 | 2 | 2 | 2 | 2 | 2 | 20 |
| **Rose et al., 2011** | 2 | 2 | 2 | 2 | 2 | 1 | 0 | 2 | 2 | 1 | 16 |
| **Sheaves et al., 2021** | 2 | 2 | 2 | 1 | 2 | 2 | 2 | 2 | 2 | 2 | 19 |
| **Shin & Joung, 2023** | 2 | 2 | 2 | 2 | 2 | 2 | 2 | 2 | 2 | 2 | 20 |
| **Strand et al., 2015** | 2 | 2 | 2 | 1 | 1 | 0 | 2 | 2 | 2 | 2 | 16 |
| **Sung et al., 2006** | 2 | 2 | 2 | 2 | 2 | 0 | 2 | 1 | 2 | 2 | 17 |
| **Sung & Puskar, 2006** | 2 | 2 | 2 | 2 | 2 | 0 | 2 | 2 | 2 | 2 | 18 |
| **Tan et al., 2014** | 2 | 2 | 2 | 2 | 2 | 2 | 2 | 2 | 2 | 2 | 20 |
| **White et al., 2021** | 2 | 2 | 2 | 2 | 2 | 2 | 1 | 2 | 2 | 2 | 19 |
| **Williams & Collins, 2002** | 1 | 2 | 2 | 2 | 1 | 0 | 1 | 1 | 2 | 2 | 14 |
| **Yen et al., 2020** | 2 | 2 | 2 | 2 | 2 | 2 | 2 | 2 | 2 | 2 | 20 |
| **Zheng & Zhang, 2022** | 2 | 2 | 1 | 2 | 2 | 0 | 2 | 2 | 2 | 1 | 16 |

CASP (Critical Appraisal Skills Programme): a score of 2 denotes that criterion is fully met, a score of 1 denotes that criterion is partially met, a score of 0 denotes that criterion is not met
