# Supplementary material for: The experience of loneliness among people with psychosis: Qualitative meta-synthesis
Source: PLoS One. 2024 Dec 31;19(12):e0315763. doi: 10.1371/journal.pone.0315763 (PMC11687762; doi:10.1371/journal.pone.0315763)
Supplement: S7 Appendix — (DOCX) [file pone.0315763.s007.docx]

**Appendix S7: Themes, sub-themes and illustrative quotations by authors and participants contributing to meta-synthesized themes**

| Meta-synthesized themes | Themes, sub-themes and quotes from authors | Illustrative quotations from participants | Source |
| --- | --- | --- | --- |
| **Theme 1:**  Loneliness in the form of disconnection  Participants described feeling like outcasts who were disconnected from the others. Loneliness was experienced at an ontological and existential level. Some felt a meaningless existence, and some feared for getting left behind. | 1. Internal Loss: Living in a Different World:  - Living with schizophrenia can therefore mean “living in another world” – the world of the psychotic experience. It is also the world of a person experiencing a chronic illness with long-term symptoms and permanent impairments as the patient's own psychotic reality conflicts with the surrounding reality.  1. External Loss: Not Belonging:  - The theme is based on the reality of literally no longer being able to take part and on the feelings of “being different,” as described under internal loss. This is where internal and external loss meet. - The respondents experienced external losses in all important areas of life. Their social network had become very small. They described the loss of friends, acquaintances, and colleagues and sometimes had not a single friend left in the world. In many cases, it had become extremely difficult if not impossible to enter into and maintain a relationship with a partner.  1. The experience of loneliness at an ontological level:  - It is at loneliness’ ontological level that loneliness’ unbearableness transforms into a splitting alienation in the form of inner strangeness and threatening guests, through which hallucinatory voices will torture and destroy the informants. - It is possible to observe the informants’ greatest loneliness – a fundamental loneliness – which appears to be connected to God having abandoned them.  1. The experience of loneliness at an existential level:  - Loneliness is experienced as aloneness and isolation from ‘normal life’. - Loneliness manifests itself as pain and is almost always present as an invisible companion, in the form of an outer isolation of the self.  1. The Construction of Disconnection: “It Made Me Feel Alienated From All My Friends”:  - Participants suggested that the experience of mental illness separated them from the rest of the world. - The interviews indicated that disconnection could be constructed with all of the reference groups. Disagreements in defining problems and the need for help could precipitate disconnection from family and professionals.  1. Social Isolation:  - Feelings of isolation did not decrease when study participants resided with other people or when they were married.  1. Loneliness:  - Descriptions of loneliness exemplified emotional detachment from others. - Loneliness related to increased anxiety, and panic led to suicidal thoughts for 1 of the participants. - The involuntary and enduring aspects of loneliness were expressed as locked in. - Loneliness was also described as lifelong.  1. Lack of Solidarity of Feelings of Nonbelonging:  - Fourteen of the 20 participants expressed awareness of feelings of differentness from other people. - Two people had thoughts about being in the wrong species.  1. Relationships and intimacy:  - Participants talked about the loss of friendships and relationships with family members who didn’t understand their illness and with the difficulty they now have relating with and connecting to the world around them.  1. The Loss of Intuitive Social Knowledge:  - It is not only one's relationship to the other that becomes disturbed in one's social life, but also one's perception of the others' way of relating to oneself is deeply affected in a very negative way. The patients describe that they experience others as having something in common, which the patients do not share.  1. The alienation of oneself:  - During a psychotic experience, interviewees reported an altered experience of some internal change; they felt alienated to themselves, even to their own experiences. They didn't recognize themselves and felt alienated to their own feelings.  1. Lost self-identity:  - For the young men in this study, rapid changes in subjective experiences and the loss of self-identity emerged as a recurring theme, linked to their suicide attempts.  1. Social isolation:  - As illustrated in Chris’s account, young men in this study often described themselves as “outcasts,” who were attempting to “engineer a life” to “reduce as much suffering as possible.”  1. Relationship quality (deductive):  - Several participants commented that feeling misunderstood, disconnected, and/or underappreciated by loved ones triggered loneliness.  1. Loneliness: 2. Not belonging: 3. ‘I was left stranded in the dark’: lacking a sense of belonging:  - A sense of disengagement and alienation from others and the world.  1. We don’t share the same reality:  - A sense of disconnect from friends because the others’ lives had not been impacted on by voices. - Hearing DTVs reduced the common ground with other people. - Voices were not a shared experience. For some, this led to loneliness.  1. To get deselected:  - Iréne was often quite alone even earlier, but she did not feel lonely: she was in demand as a wife, a mother, a grandmother and as a friend. - Her family and friends, then, had lives of their own; they did not have time to socialize and a social imbalance appeared between Iréne and those she cared about.  1. Case of Currently Fluctuating Course:  - They experienced the common themes of motivation loss in school life, loneliness, denial of self-identification as a mental illness patient, and despair due to mental illness during the deteriorating course.  1. Case of Currently Deteriorating Course:  - They encountered the common themes of verbal interaction deficiency with family, difficulty making friends, loneliness, difficulty with academic performance, motivation loss with school life, loss of interests, and despair due to mental illness.  1. “This is the greatest tragedy”– loss of intimate relationships and parenthood:  - Menashe’s lifelong experience has taught him that he, as a person with schizophrenia, is destined to live without love. - It seems that, for him, the illness prevents him from experiencing a basic human feeling. Thus, the suffering is intensiﬁed and leads him to the existential equation regarding the meaning of his life.  1. “It is hard for me to ﬁnd friends”– loss of social life:  - For Zippora, the suffering from a loss of social life is described by the gap between her old life, before the onset of the disease, and her current life.  1. Not knowing/understanding:  - The effect of not being understood had implications for the way participants felt (e.g., feeling lonely because nobody could relate to them), but it also meant other people could not provide the support they needed.  1. Barriers placed on interpersonal relationships:  - Respondents indicated that generally, they felt alone and isolated because of their mental health problems and were worried about what others thought of them. There was concern about family and close companions because they were worried, helpless and unsure about what to do.  1. *“*Symptoms ended friendships as I knew them”:  - Six participants described an experience of stagnation during their illness, as symptoms forced a period of absence from ‘normal life’ while peers continued to progress with expected milestones. Participants seemed to indicate a sense of being left behind with less things in common with their friends than before their illness. Talking to old friends seemed to make participants’ lack of progress more painful as it highlighted this period of stagnation:  1. Feelings of loneliness  - Feelings of loneliness also emerged when participants discussed their experience of being diagnosed with psychosis or schizophrenia. Participant 4 explained that he felt alone in his illness because he could not talk to others about it. - On the other hand, Participant 5 felt his friends and family were supportive, but still felt alone in that his mind was “different.”  1. Emotional comfort from a situation that affects everyone:  - Individuals with schizophrenia could remain relatively calm through the COVID- 19 pandemic because they believe they are not alone in the crisis and that everyone is struggling from the disaster. This consoled them deeply as they recognized that others who had not previously experienced significant hardship could now empathize with the suffering they had previously endured alone.  1. Spending less time with old friends: 2. Parenting  - Female participants discussed needing to attend to themselves when unwell or in order to stay well and be the parent they wished to be. These challenges were described as burdens women carried on their own, often without support or understanding from family or children, and women felt this lack of awareness and understanding further strained relationships at times. | *“Because I know you can't tell by looking at me, so at first it won't even occur to people. But that does tend to make life very complicated, and it sometimes makes me extremely lonely. Just this week I had the feeling that I was awake and* *the rest were asleep. Other people haven't been through any of this, they are sleeping, and they have no idea what it is like to…Do you know what I'm saying? And for me there is no way back.”*  *“I don't feel completely safe in the dorm, especially not with what I have. Everything seems to go too fast. I don't fit in because I am not really studying anymore anyway. Writing e-mails, making new friends—it is such hard work. How to put it…I haven't seen them for a year and I just have the feeling that I don't belong. I don't even know if I want to belong.”*  *“I heard voices and got this delusion that everyone in the city had committed suicide, and there was only me left, and then I thought I also had to commit suicide.”*  *“This is what I experience as loneliness: My God, my God why have you left me, the deep down … it sits in the chest. And he continues: it’s a sore in the soul to be left, there’s a depth to loneliness … a pain.”*  *“I’ve been put here by the Social Services without anything to do. I’m living here at their mercy, and I feel like a real case in relation to the neighbours, so I don’t have any contact with them. It’s hard to live alone, I feel forsaken, and I can’t ﬁnd any peace and quiet here.”*  “*My loneliness can be compared with an empty wall without pictures, my loneliness feels extremely cold and afterwards the anxiety comes.”*  *“I feel it in my stomach, and it is especially strong on the weekends, and the loneliness is so huge that I don’t know how to deal with it.”*  *“[watching friends move on without me] made me feel like, like I was just different from my friends. Like as if I hadn’t experienced what they’ve experienced. That’s a recurring thought for me a lot of the time.*  *“They tell me I’m not the same person I used to be, and I know that, they don’t have to tell me that.”*  *“[when I was in the hospital] nobody was giving me any reassurances. All they were doing was asking me questions... they would say, just be patient.”*  *“Even when I lived with others, they were always gone.”*  *“I’ve had relationships but they were very distant.”*  *“I felt less isolated when I was married, but I still felt isolated.”*  *“You don’t have anyone to talk to and no understanding,”*  *“being in a state of mind, nobody in a crowd can relate to you. You can be with your best friend and still suffering.”*  *“It means being without love and companionship.”*  *“Got lonely in the apartment, lonely when I’m by myself in a room. Crashes up on me, it’s a scary feeling, feel sad, feel all kinds of things. That’s when they [the professional staff] got me for suicide precautions.”*  *“You’re locked in a room all by yourself with no doors,” and “locked in your head at the world.”*  *“I’ve been described as a loner. . . . I was lonely. That’s the one thing I had trouble with.”*  *“I’ve been real lonesome. Inside or outside the boarding home, it’s still lonesome. At my mom and dad’s house, I feel lonely. I feel lonely at the boarding home, I feel lonely at my grandmother’s, and I feel lonely at the state hospital.”*  *“I don’t try to fit in. People think I’m weird. I was kind of different, I was always different.”*  *“I am separate from people, have a sense I don’t fit in and belong. I don’t know how to describe it to you.”*  *“I wasn’t part of the family, I wasn’t part of anybody.”*  *“I can understand that I feel different than other people, . . . but I can’t change.”*  *“I feel more different, being insane and having schizophrenia . . . nobody told me that I was different, but I feel that way.”*  *“I’m lost in a sea of anonymity, totally by myself.”*  *“Sometimes I feel like an alien. I’ve been almost everywhere, it’s the same. Everywhere I’ve been people never really accepted me. Maybe I don’t belong with people, maybe I belong with the animals. But I can’t talk to animals. I’ve always had that feeling.”*  *“I don’t feel I fit in anywhere, because my standards in the heart are so high that sometimes my behaviour doesn’t fit in with my standards. Even in this place, I feel I’m not one of them. I don’t fit in the church, I’m not worthy to be one of them, and in the family, I’m not well enough to be . . . they’re not happy to be with me.”*  *“Society, what society? Maybe I’ll join the dolphin clan, maybe the ocean has a society for me.”*  *“I just thought I was the one person in the world and that it wasn’t happening to anyone else in the world. That made me feel low and something different, not human.”*  *“I was spending a lot of time alone, smoking a lot of cannabis. I generally felt quite cut off from the rest of the world… Umm, because of my feelings of loneliness, I – I felt that life – don’t know, life was just very difficult… and so I thought of various ways of committing suicide.”*  *“Loneliness for me is when I feel like I can’t tell anybody that I have an issue, I can’t confide in anyone… Like nobody cares, that’s what loneliness is to me. Like nobody understands or cares that I’m going through a rough time.”*  *“We may go out and there are these women talking chitter-chatter about something I didn’t really care about, or their problems. I just felt like… there was some stuff they would talk about that was serious, but I did feel alone, a little bit, because I didn’t feel like I was connecting.”*  *“I didn’t feel like I belonged anywhere but with her. I still feel that way a lot of times…Don’t belong anywhere but in the ground with her, I guess.”*  *“I just get to the point where I figure well this is the way it’s going to be. I’m just going to be a lonely person. Or, you know, I don’t want to deal it and commit suicide. I kind of struggle between the two.”*  *“Belonging wise, I just felt like I didn’t belong anywhere. Very little value or asset to life. There are times where I felt suicidal… You start to run out of options. When you run out of options as a human, you start to question what’s left.”*  *“Why has God allowed me to suffer like this? Why has he allowed all this suffering?”*  *“[shattered trust in God as a benevolent saviour] I can’t quite figure out the puzzle,” and “sometimes reality is not quite correct in some ways.”*  *“I didn’t think I was part of it [the world], I didn’t think I was ever going to get better. I didn’t think anybody understood me, I felt alone, I wanted to achieve in the world, but didn’t think I ever would.”*  *“They have all got families of their own now and grown up, got families of their own, got careers and stuff. I just feel like I got left behind sort of thing.”*  *“I’m living two different lives at once, I have this kind of strange world in my mind and then I have the kind of physical reality of this world.”*  *“Quite, quite sad [..] Because I was the only one who was hearing voices.”*  *“Before, I often went out in the woods all alone, I was very often alone, but I didn’t feel lonely. But now I’ve noticed something that strikes me very hard, and it’s about being a burden. People explain all the time how busy they are and how much they have to do and that they don’t have time for me. And I notice this everywhere. I notice it with my children, and I notice it with my friends – not so much with my brothers and sister because they don’t live around here – but even with my very best friend I notice how she says: ‘I can’t call you so often anymore’ because she’s going to work now. She’s been unemployed for a long time, and of course it’s great that she will start working again, but all of a sudden she has not time for me at all. And this thing, that nobody has time for me, and that I must beg for social companionship with people, has hit me very hard. This has really changed to something negative, that nobody asks when I’m coming home and nobody really cares about me. And then this feeling of meaningless arises … I know it’s dangerous, the feeling of meaningless, that nothing matters.”*  *“I can’t do anything by myself. Nobody knows my dire situation. I am getting too much sleep these days; I am being stupid and getting too fat. I feel alienated from the world. I’m afraid of falling behind.”*  *“I could never find friends by myself. I’ve always been alone. Actually, I’m unaccustomed to being with somebody. So when I am with somebody, I’m uncomfortable. I don’t know what to do with them.”*  *“I’ve always felt isolated from other people. I never had a close friend to whom I could pour my heart out. I’ve always felt loneliness.”*  *“I can’t share my feeling with anybody. When I need to talk about something, I can’t touch anybody by myself. Loneliness is all I feel.”*  *“I can’t believe my current state! I’m so much worse, I feel like I’m in hell. My future is hopeless. I’ll never get married.”*  *“I have no future for a job, marriage, a family. I’m scared of what will happen to me. Gradually I’m dropping out of the world because of my mental illness.”*  *“It is sad. One cannot live without love, and people with schizophrenia live without love … It’s sad, very sad. No-one lives without love. It’s hard. It’s annoying that I have no love … I feel very jealous of healthy people, who got love and marriage and children. Only I am alone. This is the hardest thing for people with schizophrenia; the greatest tragedy.”*  *“I used to go out all the time to night clubs and cafés. I worked in a nightclub. Dancing, waltzing…all day long. My husband used to work in a factory during the day. We had a car and we’d go out to movies, discotheques … During the summer, I would travel to the mountains with my friends … as years went by with the illness, I lost all my friends … Today, it is hard for me to ﬁnd friends …”*  *‘And I didn’t want to be here, I didn’t understand what was happening to me, I didn’t understand what I was seeing. I didn’t know who the people I love around me were . . . and it was just hard and I thought . . . I just thought, “Will somebody just help me?” cos like how do I get better? I’m never going to recover from this, I’m never going to get better, I don’t even understand how I got like this. This isn’t me, where’s me gone?’*  *“It's [mental illness] had an effect on my relationships with people. I felt all alone and that I didn't want to talk to anybody. Ehmmm... I was worried about what they would think of me. I never used to go out.”*  *"It was awful........ I felt useless and helpless ....... I couldn't talk to anyone about it, I stayed in bed nearly every day.”*  *“I feel especially now they’ve all got children that their priorities have changed.”*  *“I didn’t want to talk to them because I knew it would be all about going back to uni . . .it would remind me of all the good times that I’m missing out on.”*  *“I don’t understand me, myself! I don’t understand it at the level that I feel like I could talk to someone about it”*  *“I feel like I’m different as my doctor put it…I have different moods than my surroundings, my friends, or my family or something. I know there’s something different, but I just try to not to think about it too much.”*  *“When COVID- 19 first started spreading, it was really tough for a lot of people, and many were struggling mentally. But, because so many people have been affected and experienced being in confined spaces, it felt like they might understand what I am going through, and it has helped me feel less isolated. It is like I found some comfort in knowing that others can relate to what I am going through because of COVID- 19."*  “*I do have good friends, I just, I used to have more friends though, you know, before I had the psychosis, before I ﬁrst got paranoid I used to have a lot of friends. . .I’m not really receiving much support from any of my friends, I sort of am, but I am not really getting enough, I don’t see them enough, they don’t see me enough. You know I want to see them more.”*  *“My son will say, "They don’t even know you, mom!" [when I think they’re talking about me]. And it’s hard on my son to have somebody that’s in the family--especially his mother--who has a mental illness. He doesn’t understand what I go through. He didn’t even come visit me in the hospital when I was there for pneumonia. I don’t think he wants anything to do with me...I think [my son] would rather that I wasn’t sick...I think it embarrasses him that I’m sick. He probably wants to keep it quiet around his friends, and he doesn’t understand.”* | Mauritz & Meijel, 2009  Nilsson et al., 2007  Williams & Collins, 2002  DeNiro, 1995  Chernomas et al., 2020  Deland et al., 2011  Gajwani et al., 2016  Ludwig et al., 2022  Barut et al., 2016  Bögle & Boden et al., 2022  Sheaves et al., 2021  Andersson et al., 2015  Sung et al., 2006  Avieli et al., 2016  Tan et al., 2014  Gee et al., 2003  Huckle et al., 2021  Knight et al., 2023  Shin & Joung, 2023  Macdonald et al., 2005  Firmin et al., 2021 |
| **Theme 2:**  A rejecting and stigmatising external world  Participants were mistreated and discriminated against. Some made sense of their psychotic symptoms by referring to past experiences of victimization and the resulting feelings of loneliness. Several participants found the label of psychosis and the first contact with psychiatric services damaging to their social relationships. | 1. Social Isolation:  - Social isolation from society was experienced as a stigma related to identification as a mental patient, to attachment to the mental health system, to symptoms of mental illness that interfered with employment and participation in social activities, and to observable side effects of medication. - Ten (50%) of the participants reported negative reactions from family members and from people in general that isolated them from people. - They expressed a sense of not being welcomed by others. - Some were ostracized by their families - Abuse or victimization maintained alienation and discouraged trust of people.  1. Being alienated from others:  - Being identified as a dangerous object by others led to feelings of loneliness, helplessness, and despondence, while the participants attempted to re-establish their lives in their communities.  1. Shunned:  - The examples given by the participants involved being shunned by friends, being ignored by neighbours, being isolated at school and being ignored by their family, all because of their diagnosis of mental illness. - For others, this avoidance was something too upsetting to speak about.  1. Experience of self-stigma from the outside:  - The subjects said that they had started to face discrimination or had been rejected and mistreated while suffering from schizophrenia.  1. Negative inner feelings:  - Most of the subjects said that they had to face the suﬀering process of the self-stigma alone after the onset of schizophrenia, and they seemed to feel sorrow as though they were in an endless dark tunnel.  1. Receiving information about the illness:  - Interviewees felt that society held prejudices against people with schizophrenia and other psychotic disorders, and they felt that they were being treated negatively because of their illness.  1. Anonymous Social Interactions:  - The ways in which persons felt the presence of social stigma in these diverse settings included discomfort over the sense that verbal and nonverbal communications were directed toward them to signal identiﬁcation and labelling of them as mentally ill, strange, frightening, or of lesser intellectual and social capacity.  1. Family Relations:  - Participants also describe avoidance or exclusion by family, denial of illness by family members, and the perception that family members are embarrassed or ashamed on the basis of mental illness. - A divorced 43-year-old Euro-American woman described family relations as characterized by 2 recurring patterns: either as attempts to avoid her or as efforts to make her into a kind of family scapegoat who could be pointed to as an example of someone who had more problems and was “more ill” than anyone else.  1. “They throw me out of society like garbage” - the suffering of social rejection: 2. “I have a mother but I don’t have a mother” - the complexity of family relationships:  - Gregory describes the pain and loneliness caused by his mother’s blunt rejection.  1. Not belonging: 2. Surviving alienation: 3. Social isolation: 4. The effects of being labelled (seen as different): 5. Isolation/relationship strain:  - Women discussed the signiﬁcant impact and disruption of psychosis on their social relationships Only male participants, however, discussed being perceived by others as ‘dangerous’ because of their mental illness, and this experience appeared to pose unique challenges for males as they struggled against internalizing these messages they received from others. For instance, one male explained that he became isolated after experiencing psychosis because his family members were ‘scared’ of him. Relatedly, another male participant shared his reluctance to disclose his illness and articulated the perception that doing so would precipitate the end of a relationship, this pattern leading him frequently to remain isolated.  1. Barriers placed on interpersonal relationships:  - Friends were frightened about their psychiatric status and both friends and family tried to avoid the person. There was a general perception that this was due to lack of education and ignorance about the illness. - Psychological responses to schizophrenia.  1. The development of psychosis as a turning point in social group membership:  - The experience of those interviewed in this study was that visible psychosis, or lack of concealment, often resulted in strong emotional reactions from others in existing social networks and feelings of rejection. | *“Nobody talks to me, just hello and good-bye, even the people I live with.”*  *“People react to me somewhat negatively, I’m just guessing.”*  *“People will honour a physically disabled person before they honour a mentally disabled person.’’*  *“I’ve asked for many jobs, but when you tell them you’ve got a mental illness, they look at you like you’ve got the plague.”*  *“My mother just decided she never wanted me coming home again to live . . . it seems that she doesn’t want me to be part of the family anymore.”*  *“My sister looked at me with suspicious eyes. She never talked to me even though I was recovering.”*  *“My family kept away from me; I felt so lonely.”*  *“My brother said that I am a loose cannon. Nobody cared about me.”*  *“My brother said that you had better stay in a hospital until you die.”*  *“We psychiatric patients are so problematic! Who would accept us?”*  *“My brother…he kept on telling me to stay here!”*  *“My family arranged for me to stay in different hospitals; I've been away from my home for 6 years,”*  *“My sister asked [the] doctor to keep me in the hospital [for] as long as possible!”*  *“Yes. All my friends turned away of me. They start to avoid contacts with me. They just stopped to communicate with me, broke the relations. My illness was strong disadvantage for me.”*  *“At the beginning of my illness, when my neighbours found out about it, they said: ‘This lunatic has to be left alone.’”*  *“Some do. It matters a great deal. I can’t talk about it now.”*  *“My friends were all willing to interact with me before I was diagnosed with schizophrenia. However, after I became schizophrenic, no one wanted to interact with me.”*  *“I felt that everyone downstairs was hiding from me.”*  *“ The students in the Department of Chinese call me a psychopath every time they see me… They look at me strangely.”*  *“My family views me as trash. They call me trash… They complain that I cannot work to earn a living even though I am physically healthy.”*  *“I ﬁnd it unacceptable. However… I feel very sad. When I recall my past, sometimes I shed tears…”*  *“… I am overwhelmed by such a sad feeling. It is a very pitiful and sad emotion…”*  *“I used to have friends, but they, like, took off ... so now they’re gone ... They want nothing to do with me anymore ... my friends ... I don’t know why ... It’s the mental illness they see in weird movies. Yeah, they noticed I acted a little weird sometimes ... and then are repelled by it .. . However, I got to put up with it ... You just have to ... and start over, maybe.”*  *“There’s nothing I can do about it. I get so tired of being cut off, and it’s like I’m really really frightened of it [being cut of].”*  *“I’ve never ﬁt in and I never belong.” For her, perceived stigma from persons she does not know well is so incisive to her experience of not “belonging” that it can fuel a desperate need to end her life quite distinct from the above proclivity to “just let it go.”*  *“I feel in a lot of ways mom and dad are maybe ashamed of*  *me for some reason... they are kind of hesitant and everything and... it’s a feeling, you know, they don’t know that I’ve come a long ways from what I used to be. It just seems like I’m still the same way that they think I am. You know, they just don’t realize it hurts when they go some place...ah... they don’t ask me... they just don’t want me to go. It kind of hurts.”*  *“I tried to talk to people. Only junkies would agree to accept me. The neighbours cut off all ties with me because I harassed them. They wouldn’t open their door to me … I slept out in the street. I talked to a lot of people in the streets, and collected cigarette stamps … [Silence] I felt very miserable …who would want me? They throw me out like garbage …”*  *“I’ve been through hell, I swear. I don’t wish this on anybody, and always alone, totally alone. My mother was here once for a day and ran off; she told me that being with me is torture…I went over to my parents’ house once and they threw me out. My stepfather cursed me, asked me why I am even alive…my mother is 84, I don’t acknowledge her as my mother. When she dies, I will not go to her funeral. I got nothing from her, nothing; no money, no nothing. It’s like I have a mother but I don’t have a mother …”*  *“It’s miserable because I feel like I’m at the bottom of the garbage pile, almost, if that makes any sense. Everything’s just dumped on you and nobody remembers what’s at the bottom of the garbage pile, so they empty it and throw it away.”*  *‘That’s the thing about schizophrenia, it leaves you alone emotion- ally and alone physically . . . I got really lonely. People my own age left me alone . . . the trouble with schizophrenia was loneliness.’*  *‘I was left lonely for so long it hurt, mental pain . . . I used to think what’s wrong with me.’*  *“A bit stressful cause a lot of my friends, I’ve been friends with since I was eleven years old and I’ve lost them just in the last year so … Just a bit isolated, and lonely really.”*  *“You get labelled, that’s what they say, don’t you? When you’re mad, you’re always mad. Their Dad will always says to me “you’ve always been mad, no wonder you’re on tablets” and things like that.”*  *“I wouldn’t seclude myself, and I’d get out more and do things and try to meet people. Every time I try to do something it’s just, everything backﬁres...I was going to this one church to try to meet people and made the mistake of saying that I was put on disability or something.. .and this guy goes, "So you’re the one [taking that money]!".. .so I didn’t go back and I don’t circulate with people. It just seems like every time I try to make a friend something happens.”*  *“My relationships with women, there was no problem, because I hid it most of the time. Because if they found out, it leads to the end, like ‘Oh, well, I ain’t going to go out with no nut’...so now I just keep to myself.’”*  *“Also the stigma goes along with that and you realise you're different in some way. And that's a bit of a blow when you've tried to pretend to be normal. Which I think most people do, you try very hard to appear you know like everybody else.”*  *“I can’t really go back to my old friendship group because they’re all sort of weirded out by what happened, there’s all these sort of pre- sort of like (.) judge-judgements and misconceptions and things.”* | DeNiro, 1995  Ko et al., 2022  Rose et al., 2011  Yen et al., 2020  Gunnmo & Bergman, 2011  Jenkins & Carpenter-Song, 2009  Avieli et al., 2016  Barut et al., 2016  Humberstone, 2002  Gajwani et al., 2016  Tan et al., 2014  Firmin et al., 2021  Gee et al., 2003  Hogg et al., 2022 |
| **Sub-theme 2.1:**  Sense of exclusion across the life story | 1. Relationships and intimacy:  - Pervasive in this group of women, no matter what their age, was an overwhelming sense of loneliness and isolation. - Even before the diagnosis of schizophrenia was made, loss became a dominant theme in these women’s lives—loss of jobs, relationships, and children. As the diagnosis was made and symptoms of illness interfered with the women’s ability to connect meaningfully to others, further losses occurred.  1. They cannot understand everything: “after all, it’s my problem”:  - Some participants expressed that they were facing their challenges on their own. For example, a young woman described feeling alone before her psychotic breakthrough.  1. The experience of loneliness at an existential level:  - All the informants have felt that they were lonely and different from other people before their psychotic break down.  1. Grandiose content:  - A common way to understand the symptoms was by referring to loneliness and longing for life to be different. - According to Ben, bullying victimization had provoked social anxiety and feelings of worthlessness, which also in his adult life hindered him from relating to others. A wish for vengeance was emphasized and related to his experiences of being bullied.  1. Case of Currently Deteriorating Course:  - Beginning with childhood, he showed that he was very introspective. He made few friends and was almost isolated. He had difficulties interacting with fellow students at college.  1. Loneliness: 2. Closeness and separation at specified age ranges: 3. Social Isolation:  - Subjects reported experience of interpersonal isolation as emotional separation from people that began early in life and increased with age.  1. Expectations for the Future:  - In general, the narratives of younger patients with a shorter history of illness expressed more hope for future relationships, while longer-suﬀering patients were less optimistic in their expectations.  1. Men’s experiences: Romantic Ideals and Competition:  - The brutality of the rivalry for sexual relations with women in the narratives of longer-suﬀering respondents stands in contrast to the stories of more-recently diagnosed participants. - The latter focus on their ideals—long-term, harmonious, loving relationships with women. Those are the relationships which they seek and dream of, despite having experienced plenty of rejection. For them, love is an ideal, but the naked reality they experience can be unforgiving.  1. Low Status and Discrimination:  - Persons with long-term mental illness commonly experience social stigma. They are not seen, nor do they see themselves as attractive partners in romantic relations.  1. Adversity, Advantage, and Interpersonal Relationships Across the Life Course With Schizophrenia-Spectrum Diagnoses:  - Relational voids developed longitudinally as relational losses went unrenewed or replaced across the life course, and other normative relationships did not develop.  1. Never had:  - For Hans’ part, the social difficulties had continued as an adult and after he was diagnosed with psychosis.  1. Feelings of loneliness:  - A majority of the participants reported having an absent father ﬁgure. Participant 2, who explained that he experienced auditory hallucinations because he wanted to hear his father’s voice, considered that this directly affected him. Participant 2 expressed feelings of loneliness after his father’s passing, saying that it was hard on him. Participant 3 expressed further feelings of isolation when he discussed moments he felt alone at school. - According to participants, experiences with discrimination and prejudice not only emerged from interactions with authorities but also through interactions with family and friends. Participant 5 explained the challenges he faced as a bi-racial child in Montreal and feeling excluded because of it. He stated. | *“Ehm, I felt a bit lonely in that period. And, additionally, my mom and dad were very distracted, with their family members being ill.”*  *“Yes, no, actually, eh … But in a way, I feel alone in this. But, at the same time, it’s okay. After all, it’s my problem.”*  *“I was different from everyone else, and I felt like an outsider. And outsiders are strange, aren’t they?”*  *“Loneliness is the most troublesome thing throughout my whole life. Perhaps one wants life to be different, yes. I have noticed that loneliness is the reason for my thoughts. Don’t know. If that’s a cause, then, yes.”*  *“I think it comes from being bullied as a child; I was always a lonely child. They bullied me. They commented on everything I did, for example, how I walked. They said; ‘Why do you walk like that?’… I’ve always wanted to get back at those who did it, perhaps I long for that.”*  *“I don’t think I’ve ever fit in (laugh). I’ve never felt like I fit in. Even when I was little, I never felt like I fit in. I just…I’m different in a lot of ways. But I’m okay with that usually.”*  *“When I went to school, people gave me dirty looks. I would feel uncomfortable, start to shake. . . . People used to give me a hard time in school.”*  *“In grade school, I had no friends. I tried to play with the other kids. I was separate from the other kids.”*  *“They let me join in, but then I felt lonely, like they didn’t want me around.”*  *“In grade school, [other students] bothered me. They threw stones on me.”*  *“I didn’t like school, it was hard for me to make friends.”*  *“I was to myself. Nobody really bothered me, I felt separate from people.”*  *“I wasn’t part of any groups in high school.”*  *“Didn’t feel close to people, wasn’t in any clubs,”*  *“In high school, I felt lonesome and separated.”*  *“I was always isolated, I was always by myself.”*  *“I felt separate from people, they were kind of away from me. I was never close to anybody.”*  *“I’ve always felt separated from other people. I didn’t think much about it. I never did think much about it. I’d think ‘you’re just getting separated.’”*  *“Emotional emptiness associated with isolation - Nobody calls me, nobody ever has.”*  *“I wasn’t part of the family, I wasn’t part of anybody. I would feel uncomfortable, start to shake, I would try to take a walk by myself.”*  *“That alcoholic. . . I was just sitting on a park bench. He came up to me. We started talking. Later he invited me to his place, we had sex, but . . . he said he doesn’t want to end it. . . he doesn’t want to just use me. . . He was a good guy. Unfortunately, he found out I’m in [name of major psychiatric hospital] and he didn’t want to be with me anymore.”*  *“And I notice, even to this day, how hard it is to relate to people because I’ve been let down all my life … that’s how I feel, and I begin to understand a little what it’s all about, I begin to realize that I have problems making friends.”*  *“When I meet new people, I just don’t know how to make friends,”* and at court, *“I had nobody at court. My parents weren’t at court. It was only me.”*  *“I’m not Black, I’m not White, I’m in between the two so I was, and I didn’t really know what to say or what to do so I was, I didn’t ask myselfquestions but I don’t have so much problem, like it’s hard for me to ﬁnd the right type of friends because some friends will, well some people won’t include me in the group so much because I’m always different. There’s not a lot of biracial people in Montreal.”* | Chernomas et al., 2020  Hansen et al., 2020  Nilsson et al., 2007  Strand et al., 2015  Sung et al., 2006  Barut et al., 2016  DeNiro, 1995  Budziszewska et al., 2020  Ogden, 2014  Andersson et al., 2015  Knight et al., 2023 |
| **Sub-theme 2.2:**  Stigma and loneliness in early contact with psychiatric services | 1. Getting help when you are lost: “A personal assistant”:  - Many participants described feeling lost in the system. They did not understand what was going on or why. Others called for more assistance with putting their troubles and challenges into words, particularly at the beginning of their contact with mental health services. - Some found themselves in new and strange surroundings, not knowing who to contact or how to proceed. - Several of them implied experiencing a kind of inner loneliness during the early contact with or admission to mental health services.  1. Hospitalization and Psychiatric Labelling Construct Stigma:  - Mark described feeling stigmatized by his early psychiatric experiences.  1. Impact of First Hospitalizations:  - Participants name it as a factor that most strongly pulls them out of their contemporary lives, including their relationships, and most strongly stigmatizes them.  1. The effects of being labelled (seen as different): | *“But when I was so locked up inside myself, I felt that it might have been useful to, in a way, get some help to talk […] at the time, it was a really heavy responsibility.”*  *“I mean when you’re in the hospital, the way you’re treated, you’re not treated the way you would normally treat someone in the grocery store or like a quote–unquote normal person, you’re treated less-than, you’re treated inferior than, and so it’s really difﬁcult to come out of that situation and then try to interact with other people…”*  *“I felt alone and isolated, I kind of felt like I was lower than everybody else in a certain way… I felt kind of like I had been tainted because they told me I had psychosis and that I might have schizophrenia that I might be bipolar, you know [the hospital] just kept throwing different things at me so it was hard to socialize with others because it was hard for me to feel like other people had those problems and it turns out a lot of people actually have those problems. It was just hard because I felt like I was alone, I felt like nobody really had been through what I had been through…”*  *“My partner was a girl younger than me. She left me when I was in. . .after I got out of the hospital. I think my illness terriﬁed her to some extent. Meanwhile, she said this phrase, that. . .when we were separating, she said that she has no intention of crying due to my illness. (. . .) Maybe she was scared of the future that didn’t look so rosy. Being with someone suﬀering from an illness like that.”*  *‘I received quite a bit of mocking I remember when I was being taken into hospital and that really . . . by the bloody ambulance people coming in and I really, thinking back did not need that at the time.’* | Hansen et al., 2020  Blajeski, 2022  Budziszewska et al., 2020  Tan et al., 2014 |
| **Theme 3:**  Loneliness and acute psychotic episodes and symptoms  This theme illustrated that loneliness was at its peak during acute psychotic episodes, particularly when symptoms such as delusions of persecution, paranoia, and anhedonia were visible and active. During this period, participants described socially withdrawing due to fear of acting on their symptoms of psychosis in public, and experiencing relational losses. | 1. Reduced control of behaviour and actions:  - Respondents commented that their mental health problems led them to behave in ways they would not choose to. This also involved choosing to be isolated because of concerns regarding how they may appear and trying to appear normal. Avoiding situations that they had previously enjoyed because of fear of how they would appear or whether the stress associated with these situations would mean deterioration in mental health. Other behaviours such as disinhibition, staying in bed all day, lowered personal hygiene and self-harm were also detailed.  1. To deselect:  - Several of the persons interviewed described what happened to them socially when they had a mental crisis; one did not simply have the energy to be social. Hans said that all his energy was needed to concentrate on ‘being a person’.  1. Hospitalisation and psychiatric labelling construct stigma: 2. ‘I was left stranded in the dark’: Lacking a sense of belonging:  - In crisis, Elias felt hurt and alone: ‘left stranded in the dark’. The abusive behaviour he had suffered seemed to have eroded his sense of basic trust in others as benevolent and predictable social being. As Elias lost trust in others, he experienced social relations as potentially threatening, and felt the need to withdraw. Mark also expressed the need to avoid others. - Henry described how he felt isolated, and physically isolated himself from others, because of what he was experiencing. In crisis, Henry came to inhabit a solitary experiential realm, to which he felt no one could relate. - Similarly, Matthew described a sense of disengagement and alienation from others and the world.  1. Impact of first hospitalisation:  - Several participants experienced the severing of both romantic relationships and friendships during the ﬁrst hospitalization, or some time later. Either the patients were isolating themselves from their loved ones as the psychosis progressed or their partners were leaving some time after the onset, scared oﬀ by the illness and its consequences.  1. Intimacy and emotions:  - Several participants experienced the severing of both romantic relationships and friendships during the ﬁrst hospitalization, or some time later. Either the patients were isolating themselves from their loved ones as the psychosis progressed or their partners were leaving some time after the onset, scared oﬀ by the illness and its consequences. - The personal nature of the symptoms, and the distrust that comes with certain forms of schizophrenia can all preclude any communication on this topic within a relationship. John mentioned when talking about his former partner.  1. Suspicions and distrust:  - It is diﬃcult to think of interpersonal relations as a source of support and satisfaction when such threatening thoughts overﬂow the consciousness. The blending of experiences and symptoms, along with the frequent paranoid component of the illness, can result in the emergence of deep mistrust. Suspicions, anxiety, and fear can aﬀect the patient’s attitude toward love. Negative expectations often characterized the participants’ narratives.  1. Additional influences on alienation:  - Anhedonia, defined as “the inability to experience pleasure or to imagine a pleasurable emotion’’ (Chesla, 1988, p. 410) is a characteristic associated with schizophrenia. Most respondents lacked even the anticipation of enjoyment or satisfaction, although some had experienced feeling good in the past and wanted to feel that way again. - Loneliness related to increased anxiety, and panic led to suicidal thoughts for 1 of the participants.  1. Psychotic symptoms (inductive):  - Several participants described instances of loneliness that stemmed from or were exacerbated by psychotic symptoms. Paranoia was the most frequently reported psychotic symptom associated with loneliness across interviews.  1. Social anxiety (inductive) and social anhedonia (deductive):  - Other participants noticed that social anhedonia and social anxiety served as barriers to connecting. Although participants commented on the aversive experience of loneliness, many endorsed some disinterest in engaging socially and a preference for spending time alone. Relatedly, interviewees commented that social anxiety contributed to loneliness, oftentimes preventing or reducing the pleasure gleaned during social interactions.  1. Relational losses:  - One of the participants experienced a relational loss of his or her parents, children, or both and believed those losses were connected to symptoms of schizophrenia-spectrum diagnoses. - Edward had been close with his family, however upon the onset of his auditory hallucinations, he began to experience social “withdrawal” and “estrangement” from others.  1. Reason why interacting with people is difficult whilst hearing DTVs:  - Mistrust towards the voices also became generalized to everyone. - The majority of participants felt that people around them do not understand voice hearing. For some this was due to people’s lack of experience. In some instances, the lack of understanding led to people being perceived as dismissive of the participants’ experiences.  1. Case of currently deteriorating course:  - Three participants were in the deteriorating course at the time of the study. They were getting worse positive and negative schizophrenic symptoms than before. They showed diminished participation in social activities, more absences from school, and fewer peer relationships.  1. ‘Symptoms ended friendships as I knew them’:  - Eleven participants described experiences of reduced social contact as a result of the explicit manifestation of both negative and positive symptoms - Nine participants described friends becoming integrated into their experience of their symptoms. For some this led to reduced contact as a result of fear. For others, their behavioural reaction to their friends during their illness meant that relationships were damaged.  1. Rejection by others:  - Five participants interviewed described experiences of friends ‘disowning’ them as a result of “strange” or unusual behaviour during their illness.  1. Absence of shared history: “the place of old friends is always different, you can never replace them”:  - Five participants highlighted the importance of a shared history and its contribution to a strong and reliable friendship. There was a sense that new friends (without a shared history) could not be fully trusted until some sort of hardship had been experienced (and weathered) together and so a sense of vulnerability prevailed.  1. “Sometimes I do think there is a bit of a boundary”: voices creating distance in social relationship:  - Part of this boundary involved voices causing participants to feel distrusting and paranoid about others, therefore developing and maintaining close relationships was difﬁcult.  1. Theme two:  - Sam’s illness also caused him to be uncomfortable with other people, especially in crowds, and suspicious of them. The result was social isolation. - Manuel’s illness also affected his ability to relate to other people and increased his isolation.  1. Loneliness: 2. Social isolation 3. The Feeling of Estrangement in Relationship to the World:  - The interviewees describe how the notion that what happens in the life-world is razed in psychosis. The world is no longer the safe and familiar place it was. They feel a loss of control, cause vs. effect, why things happen; none of this is clear anymore. They perceive the world as unpredictable. | *“They [voices] have affected me quite a lot. They've made me try to harm myself and do things like that, that I don't want to do. I didn't feel that I could talk to people about these things because of what they may think. I felt that I had a problem that I had to deal with on my own. Yes, I felt like that.......... on my own.”*  *“So when it happens, this thing that happened that summer, the psychosis, it is such an enormous amount of work just to stand up on your legs and be a human. You lose the energy to contact your friends and be social.”*  *“I got ill in such an overpowering way, so I had to break with my friends because they did not understand at all, they didn’t understand what was happening, they thought it was really weird that I couldn’t work…”*  *“I mean when you’re in the hospital, the way you are treated... you are treated less than, you are treated inferior than, and so its really difficult to come out of that situation and then try to interact with other people. I mean my social skills went out the window after the hospital, the second time, because I was in the hospital for a month I felt like I didn’t know how to socialize with people, it took a long time for me to get back to the point where I could like talk to people in a normal way.”*  *“I couldn’t understand why people were doing what they were to me, doing what they did to me. So like everyone treated me pretty awfully. So like I didn’t know what was happening. That made me think I’ve done something wrong and I never did anything wrong.”*  *“You know, there’s no one here like me.”*  *“I didn’t think I was part of it [the world], I didn’t think I was ever going to get better. I didn’t think anybody understood me, I felt alone, I wanted to achieve in the world, but didn’t think I ever would.”*  *“I had this method where I (. . .) [while in the hospital] cut myself oﬀ from everyone. I just didn’t want anyone seeing me in this state. My friends knew about it, but I just didn’t want anyone to see me.”*  *“My partner was a girl younger than me. She left me when I was in. . .after I got out of the hospital. I think my illness terriﬁed her to some extent. Meanwhile, she said this phrase, that. . .when we were separating, she said that she has no intention of crying due to my illness. (. . .) Maybe she was scared of the future that didn’t look so rosy. Being with someone suﬀering from an illness like that.”*  *“My ideal. . .well, right now, it’s a problem because I’m after psychosis, really severe psychosis, where I was hearing voices all day, non-stop. For me that’s a very intimate form of bond. If you hear someone’s voice in your head, and that voice is with you non-stop, then it’s like no one else can become closer to you than that voice in your head. So in a way, this feeling has remained, that the greatest form of intimacy I ever had was through the illness, and I’ll never have this rapport with anyone like that ﬂow of thoughts in my head, like a connection of thoughts. (. . .) So, for me, the perfect guy would be a guy that could read my mind and could inﬂuence my thoughts, something like in “Twilight”.”*  *“I want to have friends, I want to party. I want to feel good, I see everybody else doing that.”*  *“Got lonely in the apartment, lonely when I’m by myself in a room. Crashes up on me, it’s a scary feeling, feel sad, feel all kinds of things. That’s when they [the professional staff] got me for suicide precautions.”*  *“Well, some of it could be paranoia and thinking people are talking about me. Stuff like that. I try to tell myself; I can’t really hear what they’re saying so why should I worry about that? It’s not interesting to talk about. I feel lonely, but it helps when I test reality.”*  *“I’m a very solitary person. I don’t have many friends. I just enjoy being alone sometimes or having time to myself… The anxiety I usually have doesn’t help so I was isolating and stewing and stuff… It makes me feel less connected. Well, it was just kind of like loneliness. I pull away from the plans and I feel bad about, I feel anxiety about making the plans and anticipating the plans and sort of pull back from my family.”*  *“You can’t trust people [..] it sort of reﬂects into my social life because I ﬁnd it difﬁcult to trust people.”*  *“It’s difﬁcult for anyone who hasn’t been through it [..] to understand.”*  *“She doesn’t really understand. I think she has always been one to say that I should just get on and kind of you know forget about [the voices].”*  *“They all say it’s completely in my head and there’s no reality attached to it whatsoever, [..] no one really understands it.”*  *“Because of the kind of more of a focus on trying to rid myself of the noise it’s like socially you know I have very little to say in a [..] social situation like I’m living two different lives at once, I have this kind of strange world in my mind and then I have the kind of physical reality of this world.”*  *“Quite, quite sad...Because I was the only one who was hearing voices.”*  *“They have all got families of their own now and grown up, got families of their own, got careers and stuff. I just feel like I got left behind sort of thing.”*  *“I have nearly no interaction with anyone; I am just alone at home. Now everyday life annoys me. I lost my interests in.”*  *“I am doing nothing even though bored. As a result, I am much worse, and once tried to commit suicide.”*  *“I accused him of doing things which I believed he did, but they weren’t true.”*  *“When my mental problems started happening, I stopped being sociable with my friends. That’s when I used to lock myself away and used to have bad dreams and bad nightmares, and my friends would be in some of my dreams and nightmares.”*  *“I think I lost them before I fell ill, or I was already ill but not diagnosed. I just . . . I was not nice or polite to them anymore so we lost contact.”*  *“I went to see him and then over there I was acting really weird as well and I got arrested by the police over there. It was like a really small village and it, sort of, brought shame on them. . . .‥they found out here in London, then they just stopped talking to me.”*  *“You don’t really meet someone and you’re like ‘oh you’re my friend’; it takes a lot of tests to pass before you can say that person is a really good friend.”*  *“I thought they were gonna poison me cause the voices said they was going to.”*  *“Dealing with people and dealing with patients – it makes me sicker. . . . Going outside [is a difﬁculty]”*  *“When people get close to me, it can get screwed up sometimes. . . . It just gets a bit crowded sometimes. With everyone downstairs, it gets a bit crowded. But sometimes it’s okay when I go down there, hang around for a while. . . . Every time my mood changes, I see something different with crowds of people. . . . But the last week or two, I’ve been paranoid again. So I stay away from people.”*  *“I can’t deal with people that aren’t sick, because I think they’re smarter than me, and this makes me depressed. When I talk I don’t remember the names. . . . I’m scared to go home, because when I get sick, I hear voices of people laughing at me over there. Sometimes I want to lie down on the ﬂoor. The people no understand that I have very bad voices.”*  *“I just get to the point where I figure well this is the way it’s going to be. I’m just going to be a lonely person. Or, you know, I don’t want to deal it and commit suicide. I kind of struggle between the two.”*  *“I was spending a lot of time alone, smoking a lot of cannabis. I generally felt quite cut off from the rest of the world… Umm, because of my feelings of loneliness, I – I felt that life – don’t know, life was just very difficult… and so I thought of various ways of committing suicide.”* | Gee et al., 2003  Andersson et al., 2015  Blajeski., 2022  Bogle et al.,2022  Budziszewka et al., 2020  DeNiro et al., 1995  Ludwig et al., 2022  Ogden et al ., 2014  Sheaves et al., 2021  Sung et al., 2006  Huckle et al., 2021  Mawson et al., 2011  Johnson et al., 1999  Barut et al., 2016  Gajwani et al., 2016  Deland et al., 2011 |
| **Themes 4:**  Thwarted longing for connection  Participants expressed strong desire to connect, but were unable to do so due to social anxiety and ineptness at social skills. Financial insecurity also added to the burden of diagnosis and made it difficult for them to sustain relationships. | 1. Not belonging:  - Feeling isolated and alone compounded this lack of connection, and participants described a thwarted longing to be able to simply talk to other people in a way that made them feel accepted and understood. When this didn’t happen, it had negative impact on their emotional state.  1. Being an outsider:  - There were stark descriptions of feeling different from others and not being accepted for being one’s self. Participants described powerlessness over not belonging because of having a mental illness, and wanting to connect with others but not knowing how.  1. Loneliness:  - Participants described that the experience of not belonging resulted in feelings of isolation and loneliness that was hard to cope with.  1. ‘I was left stranded in the dark’: lacking a sense of belonging:  - Emotionally deprived, Matthew longed for connectedness and love, which he experienced as an embodied craving and described as feeling ‘hungry’.  1. Loneliness:  - When speaking of these intense feelings of loneliness, there was often a despairing quality to the discussion, and many did not have hope for increased connection with others and seemed resigned to being isolated and alone.  1. Relationships and intimacy:  - Pervasive in this group of women, no matter what their age, was an overwhelming sense of loneliness and isolation. Knowing how to connect with other people in a way that was psychologically, physically, and emotionally safe was a challenge voiced over and over again.  1. Loneliness: 2. Additional Influences on Alienation:  - Participants lacked a repertoire of responses to deal with feelings of loneliness and isolation. Twenty percent had no thoughts about specific actions that would increase their interaction with people.  1. Family:  - Every participant spontaneously discussed their family and their desire to be closer to them. - Alienation from families had multiple causes including the impact of psychosis, separation because of hospitalization and subsequent residence in supported accommodation, poverty and the death or disability of elderly parents.  1. Family Relations: 2. Absence of a companion or romantic partner (inductive):  - Several barriers to finding a partner were identified, including inexperience or insecurity around dating.  1. Case of Currently Recovering Course: 2. Case of Currently Fluctuating Course: 3. Case of Currently Deteriorating Course: 4. Isolation: 5. Spending less time with old friends:  - Overall, participants were spending less time with friends they had prior to the onset of their illness and spoke reﬂectively of wanting this to be different.  1. Assessment of current social situation:  - Three participants specifically highlighted the wish for a romantic relationship, and the absence of this was directly attributed to experiences of illness. | *“Sometimes I feel outcast, and I don’t talk a lot. I don’t know how to talk a lot unless I really have to…. It feels bad, like I ain’t got nobody to share my feelings with.”*  *“Alone is even worse, especially with such a diagnosis. You don’t want to be alone with this diagnosis or you’ll just become Galileo. You’ll go crazy in a cave. That’s not something anyone wants to be alone with.”*  *“You’re very alone. When you’re alone like that, it damages the mind. It weakens it especially. If you don’t get ahold of it or a grip of it, it destroys you ultimately.”*  *“I’ve been hungry … for life, for hope, recovery, to find my place in the world, hungry for love. I mean hunger is a basic need that needs to be … it’s to be … everybody needs … hungry for attention, everything.”*  *“I think that (managing life) would be easier with someone. It’s deﬁnitely easier to deal with any problem if you have someone to help you. (. . .) Maybe I’ve gotten a little bit used to loneliness. I mean not that sort of painful, piercing loneliness, even though it’s like that sometimes. There are times when I feel that kind of loneliness. I would deﬁnitely prefer it with someone, but time will tell.”*  *“This is as lonely as it will ever get. This is the worst. I hope it doesn’t get worse.”*  *“I’m missing things, I don’t have a relationship, don’t have a family.”*  *“I think I feel lonely because I’m not married. I think I should be married. I could take my problems to my wife and she could help me. I know there’s a woman out there somewhere for me.”*  *‘I love Dad and I think it’s good if I can spend more time with my*  *family you know.’*  *‘My Mother’s house . . . is not much to look at. It’s actually a cracker bomb palace if you know what I mean? It’s actually the bombiest [sic] place, but I just want to be with Mum, to live with Mum.’*  *“I -it bothered me a lot. I used to be jealous, I know some people where their families are really close, you know, and I don’t have that.”*  *“I would like companionship with a woman. I just like the idea of being close and people being nice to you. And you share your life with that person. There’s a bond there… I always see boys and girls, girls and boys holding hands places… That kind of makes me feel lonely. I just thought, ‘if he can get a girl, I can get a girl. What’s wrong with me?’ I mean it’s pretty much every day that this makes me feel lonely.”*  *“Sometimes I’m afraid about my future, especially finding someone to marry who will understand my illness. I am still a mentally disabled person. And the antipsychotic drugs make me gain weight. Although I have some male friends at school, I have a poor self-image and I’m afraid I’m not attractive to men. How can I ever hope to marry a normal guy? I feel really discouraged about the possibility of marriage in my future.*  *“I really want to go to school because if I could meet my friends, I don’t think I’d feel so isolation [ sic ] and alone. I’d feel like I was still a member of society. Now I can only talk with my friends by phone. If they’d be available, I’d be willing to meeting them.”*  *“I’m not the type to go out and make friends. I don’t know how to do it. But I really wish I could have a friend.”*  *‘You can’t just pick someone off the street and say ‘excuse me, can you be my friend?’ or whatever. It takes a lot of time, it takes, it just takes time to create something like that. So that’s what I’m doing at the moment.’*  *“I do have good friends, I just, I used to have more friends though, you know, before I had the psychosis, before I ﬁrst got paranoid I used to have a lot of friends. . .I’m not really receiving much support from any of my friends, I sort of am, but I am not really getting enough, I don’t see them enough, they don’t see me enough. You know I want to see them more.”*  *“Thoughts of like having a girlfriend have pretty much gone out of the window over the last couple of years since being in hospital, so that’s something I definitely think about.”* | Barut et al., 2016  Bögle & Boden et al., 2022  Budziszewska et al., 2020  Chernomas et al., 2020  DeNiro, 1995  Humberstone, 2002  Jenkins & Carpenter-Song, 2009  Ludwig et al., 2022  Sung et al., 2006  Tan et al., 2014  Macdonald et al., 2005  Huckle et al., 2021 |
| **Sub-theme 4.1:**  Psychological barriers hindering potential connections | 1. Intimacy and Emotions:  - The desire for intimacy and closeness is a deep-seated need. For Alice, the intense experiences associated with psychosis stand in stark contrast to her normal life and the relationships in it, relationships that do not have the same immediacy and intensity. - After having experienced psychosis, Alice sees herself as muted, unable to feel as intensely. She relates her unreadiness—for initiating romantic relationships, for falling in love—to her diﬃculties in experiencing things.  1. Identity Work and Adjustment:  - For many participants, the experience of psychosis resulted in the need for a redeﬁnition of the self-image and their image of the world. - First-time illness experiences seem to pull patients out of the world of interpersonal relationships and drag them into loneliness.  1. Suspicions and Distrust  - The blending of experiences and symptoms, along with the frequent paranoid component of the illness, can result in the emergence of deep mistrust. Suspicions, anxiety, and fear can aﬀect the patient’s attitude toward love. Negative expectations often characterized the participants’ narratives. - Other patients’ narratives also highlighted that the eﬀort put into making sense of the fragmented experience, including sexual experience, can be remarkable.  1. The Loss of Intuitive Social Knowledge:  - While psychotic the respondents describe that other people's behaviour suddenly becomes difficult to interpret. - The individuals seem to have lost what is usually called their "common sense," and they may have a hard time remembering how they should be and behave with others. - It becomes difficult to spontaneously throw oneself into a social situation. Instead the social contact is characterized by distance and cognitive calculation on the part of the psychotic subject. - The experience of psychosis is characterized by a deep sense of loneliness, involving an experience of not being able to communicate. - Another interviewee describes a desperate quest for contact with others, yet he no longer clear how to begin. He has a certain awareness of the wrong way approach others, but is unable to compensate. He is afraid of making mistake afraid to connect the wrong way.  1. Relational voids:  - Participants directly connected relational voids to their experience of symptoms of schizophrenia-spectrum diagnoses or to consequences of the diagnosis that they had identified. - For all participants, the experience of relational voids involved a sense of loneliness and of having missed out. - Participants believed the relational voids in their lives told of schizophrenia’s toll through dreams of a relationship that had not come true.  1. Experiences Involving Interactions with Friends:  - Loneliness was operationalized by such instances as the inability to share deep feelings (10), feeling withdrawn from friends (9), feeling alone (6), and feeling disregarded (2). - These experiences were defined as difficulty with making friends. These difficulties may have been related to their psychiatric symptoms and their developmental needs.  1. Social isolation:  - Ineptness at elementary social interactions and the inability to execute social interactions appropriately increased the distance between the participants and society. - When participants misunderstood expectations in a social setting, their consequent inappropriate responses resulted in rejection, exploitation, and even incarceration.  1. Friendships facilitating the ability to cope:  - However, there appeared to be a somewhat anxious dilemma between needing friendships to cope with voice hearing, but ﬁnding these difﬁcult to maintain. | *“My ideal. . .well, right now, it’s a problem because I’m after psychosis, really severe psychosis, where I was hearing voices all day, non-stop. For me that’s a very intimate form of bond. If you hear someone’s voice in your head, and that voice is with you non-stop, then it’s like no one else can become closer to you than that voice in your head. So in a way, this feeling has remained, that the greatest form of intimacy I ever had was through the illness, and I’ll never have this rapport with anyone like that ﬂow of thoughts in my head, like a connection of thoughts. (. . .) So, for me, the perfect guy would be a guy that could read my mind and could inﬂuence my thoughts, something like in “Twilight”.”*  *“The thing is, right now my emotions are sort of extinguished, they’re the opposite of vibrant. (. . .) It’s just that some things don’t reach me, or they bounce oﬀ.”*  *“Because if you’re falling in love, those emotions have to be there. They just have to . . .”*  *“The beginning of this illness is that you don’t know what you want, where you stand, and who you are. You have millions of questions in your head so you just forget about stuﬀ like love. It’s set on the back burner, right? Only after a certain period of time, when you pull yourself together, you start thinking about stuﬀ like that, right? The ﬁrst stage is ME, only.”*  *“I didn't approach anyone ... no way. Then I just drove around in my car. Then I went home and slept, and I changed my mind when I got there, so to speak . . . So you had decided something in advance , but it didn't turn out as you had thought , or what? Yes exactly, I didn't dare make a mistake, because it would be, like, wrong to meet someone by just going up and ringing the doorbell. So I get there and decide ... 'nooo ... I don't fucking dare, I don't dare do a thing, because I am so damn weird right now.' I understood, I understood at the end that I was in a terrible shape, so therefore, ... nooo, I didn't dare, so I drove home again. Or went to sleep when / . . . / so it ended with being alone in my apartment for ten months or thereabout.”*  *“I can’t cope with the problems that come with having a husband . . . [but] I’m sorry that I didn’t have more children.”*  *“[schizophrenia spectrum diagnosis had contributed to] a lot of loneliness—a lot of loneliness...I would have liked to have had a family...And I would have liked, in lieu of a family, to have some sort of decent sex life. That would have been nice.”*  *“The dream that I have hasn’t come true...The person of my, the man of my dreams—I ain’t gonna lie—The man of my dreams, and we have a beautiful home with everything nicely done in it and everything...I probably would have been all that. And then I would have said I was successful. And not just say “I” was successful, I’d say “we” was successful—I would not exclude him! Now we are successful! Now we’ve made it. Now we have it all. And then we would return our minds over to our religion... because when you have everything then we can go into our religion and live a nice quiet life, a quiet peaceful life.”*  *“…if I ever undervalued friendship, I don’t now, you know? Deﬁnitely don’t undervalue it. It’s really tough because it puts you under a lot of pressure as well to maintain contact with people and you can’t always deal with that pressure all the time doing that all the time...”* | Budziszewska et al., 2020  Deland et al., 2011  Ogden, 2014  Sung & Puskar, 2006  DeNiro, 1995  Mawson et al., 2011 |
| **Sub-theme 4.2:**  Structural barriers to social contact | 1. Physical barriers to adequate social engagement or community involvement:  - Participants described feeling unable to connect with new people or adequately engage in social activities due to financial limitations and issues with transportation. Furthermore, many interviewees were economically inactive at the time of the interview and described their primary source of income as disability payments and/or financial support from family.  1. Wanting but not getting:  - Klara was longing for friends and for love but felt trapped in her apartment, with very limited economic resources to convert her social longing to social places. She would have loved to go out to public arenas such as pubs and restaurants. It was hard for her to stand the loneliness.  1. Poverty:  - Currently, the frequency of Eva’s social outings and interactions—both romantic with her special friend and non-romantic with her other friends—is determined by the contents of her wallet. - Participants often see ﬁnancial resources as a deterrent to attractiveness and independence in establishing and maintaining relationships. Poverty adds to the illness and becomes a determinant of multiple, cross-sectional stigma.  1. Work:  - Both men and women identiﬁed negative consequences from a disrupted work trajectory due to psychosis. - Men, in particular, reﬂected on the effect of a lack of work on their ability to achieve particular work milestones compared to their family or peers. Many men made social comparisons and discussed a sense of shame that contributed to self-initiated isolation.  1. Not being accepted by the society:  - Nearly all respondents strongly believed that they were excluded from society and had experienced social rejections. Specific shows include discrimination and prejudice, sarcasm and ridicule, social isolation and lack of job opportunities. | *“I didn’t drive. It did add to my isolation and my loneliness… I just spent more time in my room, but I drive now, and it adds to my freedom and ability to be around people… I’m broke and don’t have money to do things… I mean I have disability to pay my bills, but I don’t have money to do extra stuff. I don’t want to bring up [hanging out with my best friend] because I don’t have money to go out and I feel like I would only be able to say, ‘Hey, do you want to just come hang out and sit around my apartment.”*  *“You are like alone all the time. I’m alone in my apartment and I’m alone when I sit at the unemployment office, and the only persons I see are my mum and sometimes my only friend. It’s not enough, I need a bigger and more solid contact network.”*  *“It’s not only about the money. It’s about status, social status. If you have a job where you are appreciated and successful, you get an entirely different status, and you emanate self confidence that makes people drawn to you, people get interested in you, want to find out who you are and what you think and so on. But if you appear as a bloody loser and think ‘I have no job, I have no money, I have no health, I have no sense in my head …’ then it’s, of course, meant to fail. You might as well go to bed….”*  *“That relationship didn’t survive because we didn’t have, I didn’t have money. (. . .) That’s why I knew that after high school, I’ll probably go on disability. I had no way of supporting myself, no work opportunity, and he didn’t have work opportunities either, (. . .) The problems were mainly ﬁnancial.”*  *“I couldn’t count to ﬁve in my family because I’m not progressing like I should. I am not moving on in my life, sowhy should I be around them. Everybody else may be, but I’m not. Everybody else got a car, families, phones, and I’m still struggling.”*  *“Even my family members don't like me, let alone strangers. I know other people have looked down on me and call me a lunatic. Nobody wants to hire people like us (persons with schizophrenia). Some shopkeepers even prevent me to enter the store, for fear of affecting their business.”* | Ludwig et al., 2022  Andersson et al., 2015  Budziszewska et al., 2020  Firmin et al., 2021  Zheng et al., 2022 |
| **Theme 5:**  Paradoxes of loneliness and psychosis  Nearly no participants lacked desire for belongingness, but the majority socially withdrew to protect themselves from rejection and stigmatization. | 1. Loneliness:  - Others seemed to have hardened themselves to the experience of not belonging and described the experience with a self-protective distance or an attitude of indifference. - Participants who described this attitude of indifference also made contradictory statements about loneliness or wanting to feel a sense of belonging.  1. ‘I was left stranded in the dark’: lacking a sense of belonging:  - Several participants expressed the need to isolate themselves as others were perceived as imminent and existential threats. Their fearfulness and sense of abandonment was contrasted with a desire for connectedness and belonging. - Mark expressed the need to avoid others. He explained that he would like to build a wall around himself to protect him from others.  1. Intimacy and Emotions:  - Not all patients experience their symptoms as something to be shared with their partners—the unusual character of the experiences can also lead to secrecy.  1. Stigma and rejection: 2. Relationships and intimacy:  - Making new friends or re-establishing connections with old friends was socially difficult and could be emotionally threatening, but not making friends resulted in lives of isolation and loneliness. - Intimate relationships had proved too stressful for many of them, and they cited the strain of relationships as a reason for relapse. Some women resolved their dilemma by choosing to be alone. - Furthermore, many women reported histories of childhood abuse, rape, and physical abuse, resulting in an aversion to and fear of sexual intimacy.  1. Different kinds of friendships: “friends with whom … I am just myself”:  - Most participants described having few but close friends. For some, it was a conscious choice, while others seemed to have previous negative experiences with people, which may have made them wary of trusting others.  1. Anonymous Social Interaction:  - The ways in which persons felt the presence of social stigma in these diverse settings included discomfort over the sense that verbal and nonverbal communications were directed toward them to signal identiﬁcation and labelling of them as mentally ill, strange, frightening, or of lesser intellectual and social capacity. - Participants attempt to de-emphasize illness and do not reveal their illness to people other than close friends and family.  1. Acquaintance Relations: 2. Solitude versus proximity to others (inductive):  - Interviews revealed a tendency for persons with psychosis to self-isolate when not doing well. Some reported a general sense of “overwhelm” or perceiving social interactions as too stressful and effortful.  1. Social anxiety (inductive) and social anhedonia (deductive):  - Other participants noticed that social anhedonia and social anxiety served as barriers to connecting. - Although participants commented on the aversive experience of loneliness, many endorsed some disinterest in engaging socially and a preference for spending time alone.  1. Internalized stigma (deductive): 2. Coping strategies:  - During conversations about the utility of different approaches, participants described some apprehension about talking to friends/family about loneliness. - Some noted concerns about being perceived as a burden or appearing too “needy.”  1. Need for solitude:  - A need for solitude was expressed by five of the seven participants and describes the need for respite from challenges presented by socializing. - All participants whose narratives included this theme also experienced problems that they connected to relational voids or isolation and that equalled or surpassed the intensity of expression of his or her need for solitude. - There were no participants who lacked all desire for human contact.  1. Social withdrawal:  - There is a two-way relationship between this and ‘shunned’. - Being shunned may lead to social withdrawal but social withdrawal may lead the individual to actively cut themselves off from society. - Other respondents describe isolating themselves because they are afraid that they will be rejected if they disclose their diagnosis.  1. Relationships too emotionally demanding:  - Some participants avoided certain circumstances (e.g. marriage and having a family) because they believed that they would be too stressful.  1. Despair due to mental illness: 2. Case of Currently Deteriorating Course: 3. Experiences Involving Interactions with Friends:      1. External Loss: Not belonging:  - Respondents identified that their thought disorder led to communication problems, they deliberately avoided social situations to escape stress, and their friends and acquaintances simply did not understand. - On the basis of the interviews, it may be assumed that patients with schizophrenia experience double isolation: isolation that results from the primary symptoms and isolation due to consequences of schizophrenia. - Participants described being alone because of their illness, and some articulated that being alone with the illness was detrimental to their emotional and mental health.  1. Alienation from interaction with others:  - The subjects also indicated that they felt shameful to have suﬀered from schizophrenia. They cared about the strange attitude exhibited by others towards them, and were worried about the negative image portrayed of them when they were described as a “psychopath” or as having “mental illness.”  1. Feeling support by significant others:  - However, some participants felt that they lacked this source of support or felt unable to seek input from family or friends because they did not want to be perceived as a burden or to be rejected.  1. Taking part in social contacts:  - Interviewees felt that society held prejudices against people with schizophrenia and other psychotic disorders, and they felt that they were being treated negatively because of their illness. - The interviewees spoke of a tendency to isolate themselves, especially when they felt unwell. They expressed a desire for someone to come to their home on these occasions to help them out of their isolation.  1. Psychosis, identity and desirability:  - Additionally, participants expressed internalized stigma and objectification related to the intersection of inferior social status/identity associated with a psychosis label as well as gendered norms regarding desirability, reflecting a sense of inadequacy and low self-worth.  1. “What I’m scared about most is rejection”:  - Related to prevalent misconceptions about mental health and psychosis in particular, and internalization of illness models, participants voiced concerns about the risk of rejection. - Partial disclosures or general conversations about views of people who experience mental health difficulties were a way to ‘test the water’ with partners who may hold stigmatizing views and protect the self from direct judgement and/or rejection.  1. The Construction of Disconnection: “It Made Me Feel Alienated From All My Friends”:  - Almost all of the respondents expressed fears about what society and the local community believed about the mentally ill. - These interviews indicate that people with schizophrenia might withdraw from the world, but the withdrawal might also be a response to rejection from the people around them. Therefore, the disconnection is a construction between the individual and his or her environment.  1. To deselect:  - On the one hand, Anna deselected her friends in order to cope with her psychosis and because they did not seem to understand her experiences of mental illness. On the other hand, one could say that the friends deselected Anna as well in that particular situation. Thus, a mutual deselection took place.  1. Never had:  - Several of the persons interviewed described what happened to them socially when they had a mental crisis; one did not simply have the energy to be social. Hans said that all his energy was needed to concentrate on ‘being a person’.  1. The development of psychosis as a turning point in social group memberships:  - This interruption of social relationships and social functioning manifested in common ways. Some participants reported trying hard to appear normal and conceal their psychosis for fear of being judged but experienced this as putting a strain on themselves and their relationships. - Given the concealable nature of psychosis, people are often faced with difficult decisions about disclosure: whether to disclose their psychosis and risk the stigma, prejudice and discrimination that might ensue.  1. ‘Sometimes I do think there is a bit of a boundary’: Voices creating distance in social relationships:  - In direct contrast to the usefulness of social relationships in coping with voice hearing, some participants discussed isolating themselves both physically and emotionally from other people. - For example, participants discussed how other people did not understand their experiences; by creating social distance, participants could avoid uncomfortable or unsupportive responses.  1. Ambivalent feelings regarding social distancing:  - Wearing masks and the safety of staying in their homes helped alleviate stress and psychiatric symptoms as they could not interact much with others in person. However, they yearned for social interactions. They wished for a friend to confide in and escape from their internal worlds through such friends.  1. Self-stigma and low self-confidence:  - Patients’ self-stigma and low self-confidence were mainly displayed in self-isolation during daily life. | *“I really can’t say that I want to be a part of anybody else’s life, or something like that, you know what I mean? So I’m not trying to belong with other people. I’m just trying to be in society, mind my own business, live my life, and do what I want to do with my life, and not be involved with others.”*  *“At times, I feel like, uh, just hearing voices, stuff like that, maybe I feel like I’m … uh, I guess maybe I get lonely at times.”*  *“I couldn’t understand why people were doing what they were to me, doing what they did to me. So like everyone treated me pretty awfully. So like I didn’t know what was happening. That made me think I’ve done something wrong and I never did anything wrong.”*  *“I thought if I had no people around me, like just me, just me like that and then all these people […]. That could be like my family and stuff and my friends and the group members. I would like to build like a wall that would protect me so I would just be like that in this whole thing [draws a line that separates himself from others].”*  *“I’m afraid of rejection, that if I were to get emotionally involved, like it’s been many times before when I got involved with this or other guy and later got rejected. . .It was a very sad emotional state.”*  *“I don’t tell. I just find that telling a person . . . they don’t understand . . . especially with schizophrenia, they think they’re going to be murdered by you, so I don’t like to mention anything.”*  *“It’s really hard to find good friends, you know, like me. I had to stay away from people who drank, used drugs, and I’m in low-income housing . . . it’s really hard.”*  *“I don’t know. I have like … It didn’t go so well with friends in the past. Eh, been bullied a lot. By people who used to be my friends … So, it is, in a way, a bit hard to be able to, like, trust people. Although … I really want to.”*  *“Well, I, some people just come out and say, you know, I’m*  *sick or you know, I got a problem. I’ll just try to, try to hide it. (Uh hm.) I would just try not to make it obvious. (Uh hm.) Because some people can be really cruel, you know. Um, they can say, ‘well what’s wrong with her, you know? You know how people are. They, they can just kinda get funny about that kind of stuff.”*  *“I try not to let them know that I have a mental illness, because the minute you do, that’s it. They gonna not be bothering, they’re gonna talk about you, they’re gonna downgrade you.”*  *“It appears that establishing new relationships often seems difficult due to worries about disclosing mental health issues and the impact of symptoms on successfully connecting with others.”*  *“I’m a very solitary person. I don’t have many friends. I just enjoy being alone sometimes or having time to myself… The anxiety I usually have doesn’t help so I was isolating and stewing and stuff… It makes me feel less connected. Well, it was just kind of like loneliness. I pull away from the plans and I feel bad about, I feel anxiety about making the plans and anticipating the plans and sort of pull back from my family.”*  *“I think [loneliness] comes from the society telling me what’s normal. I have a problem with, I think I’m a very weird person… I sort of, I label myself and it really puts myself down… I have a lot of, uh, that’s a word that’s like stigma on myself.”*  *“When I first got to [city] I met a girl, some other people I’ve met. She knew I had a problem like this but I liked her a lot but I couldn’t, I just didn’t keep in touch, she went back to [country] and stuff. A few things that happened like that where I could extend myself further but it was early on when I didn’t want to, I didn’t know what to say. I felt awkward, so awkward that I didn’t know how to bring it up. I just didn’t do anything about it and I let the relationship go.”*  *“I avoid from starting a personal relationship because I fear that my partner would say ‘you are ill and that’s why you speak like that’ even if I’m right.”*  *“But I try not to speak too much about myself. I am afraid later the rumour that I had been staying in this ward can get spread.”*  *“I cannot be with a person without him knowing that I have a problem, it is not a huge problem, but it is a problem. I will have to take drugs my whole life, so, I think that this complicated, I feel insecure to disclose to the person and the person might become frightened and disappear and I then will suffer, so, I never more had a relationship, never more I was open to any relationship.”*  *“Actually I never told anybody outside of my family about my illness. It’s not that I shamed of my illness, it’s just that I know some people might stigmatize me as being some kind of ‘insane’ person. That’s a hard thing to handle. Sometimes I worry about my future, especially about finding someone to marry who will understand my illness.”*  *“I’m very afraid to let anybody know about my mental illness. I feel terribly burdened by my illness”*  *“I’d always been withdrawn from schoolmates. I was extremely ambivalent about being with somebody. When I was alone, I felt comfortable; at the same time, I was so lonely!”*  *“I would rather run away from all those things; I just can't focus. It's all so painful, a kind of paralyzing sadness. That is what I run away from. I don't want to face it. I prefer not to talk about it.”*  *“I try to stay self-contained and escape from the public.”*  *“I seldom interact with people, and I cannot face my friends.”*  *“Well, you can bounce off them [friends], can’t you? But if you become a pain, then you’re back to suicide on your own because they just reject you.”*  *“Well at times we’re left alone for quite a long time during the day and I find it quite lonely sometimes . . . I had schizophrenia and I was sort of a lonely person and I was quite withdrawn, really quite withdrawn some days on my own and quite lonely . . . I think it’s part of the illness spending lonely periods on my own.”*  *“When I get sick, I just lie on the couch ... I do everything on the couch, I eat on the couch, watch TV on the couch, sleep on the couch ... and everything else falls apart ... I fall apart ... And then it’s good if somebody comes and snaps me out of it.”*  *“I think until society changes some of its perceptions about what it’s like to be a schizophrenic, what the reality is for most of us, you know they’re not sort of raving homicidal lunatics all the time, that it’s going to be difficult because it’s against that backdrop that you drop the bombshell, so to speak, about what your diagnosis is …”*  *“I do remember telling my former partner that I was struggling with, I called it ‘depression’, I said ‘I’m suffering from depression at the minute’ I said ‘I’m taking tablets I’m seeing a counsellor’ things like this erm just to try and test the waters if that makes sense to try and get some feelers for what he would how he would react to that.”*  *“I don’t really talk to anybody about it . . . I feel almost embarrassed.”*  *“So when it happens, this thing that happened that summer, the psychosis, it is such an enormous amount of work just to stand up on your legs and be a human. You lose the energy to contact your friends and be social.”*  *“… if I just try and sort of carry on it’s almost like the act of trying to hide it makes it even more obvious and changes my behaviour so much that then it’s more noticeable.”*  *“Well I’ve actually not thought of talking to anyone because it’s like, whoever I go, if I go to talk to someone they don’t understand.”*  *“This disease (schizophrenia) is rather special, no one is willing to socialize with people with this disease. So, I’d rather stay alone, lest others disgust me.”* | Barut et al., 2016  Bögle & Boden et al., 2022  Budziszewska et al., 2020  Chernomas et al., 2020  Hansen et al., 2020  Jenkins & Carpenter-Song, 2009  Ludwig et al., 2022  Nilsson et al., 2007  Ogden, 2014  Rose et al., 2011  Sung et al., 2006  Sung & Puskar, 2006  Mauritz & Meijel, 2009  Yen et al., 2020  Harris et al., 2019    Gunnmo & Bergman, 2011  White et al., 2014  Williams & Collins, 2002  Andersson et al., 2015  Hogg et al., 2022  Mawson et al., 2011  Shin & Joung, 2023  Zheng & Zhang, 2022 |
| **Theme 6:**  Well-being embedded in the social world  Participants emphasized unwanted solitude provoked loneliness. Connecting with people who had similar experiences helped offer a sense of purpose and hope. Some participants described a positive form of loneliness as they substituted social interaction with solitary activities. | 1. Relational adaptations:  - One participant described in detail the complexity and importance of her relationship with her psychiatrist of 20 years. - Continuity and reciprocity were ongoing relationship challenges in provider-client relationships. - Furthermore, participants expressed that they would feel lonely, “lost,” and/or “adrift” without their treatment provider.  1. To deselect:  - Karin said in an interview session that she was socially active and had several friends that she socialized with. She was never alone if she did not want to be. - Karin did not want to socialize during a period of ‘feeling down’. This partly reflects that she had choices – her temporary state of being alone was in a sense self-imposed – and can also be seen as an act of enhancing well-being.  1. Sense of Belonging:  - Having a sense of belonging included contact and connection with others, and when the person felt a sense of belonging, it generated positive feelings.  1. Being with Others:  - Four participants described that being with others helped them to cope with the symptoms of schizophrenia, and in some cases being with others even lessened those symptoms. - The implication of reciprocity suggests that sense of belonging entails more than just having social support or social contacts, it may also include having opportunities to be supportive in return.  1. Peer-based versus normative relationships (inductive):  - Unwanted solitude appears to instigate or exacerbate lonely feelings. - Although having a peer community involves many benefits, including increased structure (e.g. visiting Club Houses) and regularity of social contact, many participants endorsed wanting to engage with individuals outside of their peer community.  1. Impact on wellbeing:  - The reciprocal nature of romantic relationships was also considered a component of wellbeing for participants. Being loved and cared for, but also caring and loving another person in return positively impacted one’s sense of belonging and connection.  1. Positive withdrawal:  - What emerges from these intricate parts is not an isolated or meaningless behavioral eccentricity, but a complex equilibrium – or a superordinate, general style of relating to the social world – corresponding to the concept of positive withdrawal.  1. Getting better: What can friends do to help?:  - Participants emphasised the importance of having friends around to distract from ruminating about the illness rather than focusing on or discussing illness-related experiences. - There was a shared idea that friends could provide support for participants to get back involved with everyday life, and to resume activities which might have been difficult to initiate alone.  1. Friendships facilitating the ability to cope:  - Perhaps to counteract their beliefs about the abnormality of voice hearing, participants also spoke of a need for normality, which was gained from social relationships. - Participants believed voice hearing was to some extent caused by social isolation, and as such participants placed great value on social relationships. Their ability to cope in day-to-day life and with their voices was compromised without the support of family and friends. | *“He helped me a lot, but...I was too dependent on [him]. On the other hand, I had a lot of crises in my life and he was there for me...He provided me with an intimate relationship... [and] a support structure.”*  *“Reliable people that I’ve worked with for years....I’d be worried if I lost them—to go out on my own.”*  *“I usually see people at weekends, but sometimes I don’t feel like it –‘No, now I want to stay at home and watch TV’. But more often I get together with a friend and eat dinner, do something, go to the cinema or sit and talk, that’s the usual stuff. I don’t feel lonely, and I always have someone to call, and if I want to do something most of the time I will find somebody to hang out with.”*  *“I’ve been quite alone these past six months, I haven’t felt like seeing anyone. It’s not that I have become more lonely than I was before, it’s more that I’ve been feeling down and haven’t wanted to see anyone.”*  *“It means to be part of a group where they don’t pick you out and say, “Look, this person’s something that we don’t want to be.” It’s to be part of a group and to be included in kind of a category within a group of people that people want to be like, that this person that fits into the idea of what we should be.”*  *“It feels pretty good to belong to something, it… makes you want to wake up and keep doing the things you have been doing every morning” “It makes me normal. It makes me feel like I’m really doing something right and really special in somebody’s life.”*  *“It’s like a sponge. Sometimes I’m filled with water and sometimes I’m not, just depending on how tightly the voices are squeezing at me. When I’m alone, it’s usually at it’s worse. I try to, as much as possible, try to integrate myself back into society because that’s the only thing that can fix it.”*  *“When I’m present with someone else, it’s much easier to manage. It’s much easier to control. It’s much easier to manipulate what’s really going on mentally.”*  *“You know it feels really good to be around ‘normal’ people… someone that’s a non-alcoholic or whatever. When you’re out there in the real world, working, you’re not in this protective meeting where everyone’s like you. You’re not in group therapy and everyone has the same mental health diagnosis. You’re out mixing in the community and being human… You’re just you, no label.”*  *“… with the company of somebody else it’s a bit easier sometimes to cope with life and social situations.”*  *“… it’s just really nice to be close to her and I can tell her what was going on with me (pause) suppose it made me feel needed, that she needed me and that I needed her in return.”*  *“I think it would have helped me mentally, with my mind, just not thinking about problems, thinking about the bad all the time – negative, negative, negative. Instead of thinking about the positive things in life, the good things, the simple things in life.”*  *“You need someone to help you go out more, like go to the cinema‥ like play football, like someone who’s a really close friend.”*  *“We have a good chat, not just about illness, well we don’t exactly bang the world to rights but we talk about football which is alright I suppose…”* | Ogden, 2014  Andersson et al., 2015  Barut et al., 2016  Ludwig et al., 2022  White et al., 2021  Nilsson et al., 2019  Huckle et al., 2021  Mawson et al., 2011 |
| **Sub-theme 6.1:**  Connectedness within mental health services | 1. The importance of support group:  - His description of the reciprocity of the group relationships is notable.  1. To deselect:  - A social context that during a certain period of life seems self-evident and supportive can at other times seem shackling and obstructive. One no longer wants to identify oneself with other persons with SMI.  1. Connecting/Being a Part of Something:  - Four participants identified that the primary place that they felt a sense of belonging was with other people with mental illnesses. - Belonging was related to setting goals or having a purpose in life. There was hope in being with other people they could relate to and in communicating and connecting with others.  1. “Yeah, I Know What That’s Like”: Connecting With Peers to Resolve Stigma:  - Here Mike talks about how connecting to other young adults with psychosis helped him move past feelings of abnormality and self-stigma.  1. Social isolation:  - Relationships with other patients in the hospital were avoided. Respondents were distrustful and openly critical of other patients.  1. Shared meaning and burden:  - Mutual support through shared experience was perceived as helpful in the recovery process. Good relations with their current mental health team, including access to talking therapy and peer support groups was seen as important to recovery.  1. Taking part in secure professional relationships and social contacts  - Interviewees spoke of the risk of becoming lonely and isolated and how important the opportunity for social contact is for well-being. Therefore, the respondents were attending meeting places and expressing the wish for receiving home visits. - They addressed the value of building up a more personal relationship with the healthcare system, and they were striving to be viewed as individuals and not just one in the crowd. - When interviewees were experiencing a regular, personal, and long-term contact with the same professional they pointed out how safe and useful it felt, even in those cases where they did not meet very often.  1. Talking to people:  - Talking to others was perceived as important and necessary for improving well-being and not feeling alone. - Finding out that there were other people with similar experiences, through talking, made participants feel less alone and gave them the perception that they can get through times of crisis. - The therapeutic effect talking had on the individual was a worthwhile endeavour, potentially reducing the psychological distress of psychotic and suicidal experiences and improving confidence.  1. Other people who have experienced mental illness and mental health professionals:  - Other individuals with schizophrenia could be a source of great encouragement if they were functioning well.  1. Identity compartmentalisation:  - Participants felt that retaining a sense of agency and choice over their group memberships due to the concealable nature of the psychosis identity provided a sense of control when faced with the confusing and distressing experiences of psychosis. - It is likely that there is a critical time for individuals with psychosis within which they may be more likely to seek contact with others with similar experiences. This might relate to the meaning attached to the psychotic experiences or how present and debilitating the experiences are at any point.  1. Hanging out with people I like and who understand me:  - In addition to talking to peers in the group program about personal problems, participants received advice and engaged in problem solving to feel supported in managing their illnesses.  1. A need to step away from services:  - Five participants explicitly said that they would prefer to avoid service-led activities and rejected the opportunity to engage with other service-users. - Interestingly, four of these participants had also identified a shared experience of psychosis helpful, but their rejection of services seemed to be because of wish to avoid re-visiting the distress of their experience. | *“Stuff goes on in my life. And I can discuss the problems in group . . . Sometimes I just feel depressed in my head...or just not good. It helps me to sit in there. It helps just to sit and listen...and I honestly like to help people out...The clients have their problems, and [I] give them advice... Give them feedback...I get a lot of feedback and it helps me.”*  *“I got so tired of all those sick people, sitting there, staring at the ground. ‘What the hell, wake up!’ And if I don’t make conversation, they just stay quiet. I got so tired of that, I freaked out and said: ‘I’m leaving now’. But it’s not like I have something against people that are ill, that’s not it. There are some really nice people there but I just couldn’t cope with it. I wanted to do something else, something worth something to me. I felt stuck at that place.”*  *“(Belonging means) constantly making contact with friends, no matter how few you have, family, girlfriend, whatever. You have to maintain those things in order to have a belonging, at least I feel so. Those are the things that make up a human.”*  *“Because there is this tight-knit community of people with mental illness. And they will stick together and be supportive and try to help you through anything. So if I belong anywhere, I belong with people with other disorders or mood disorders or anything.”*  *“…it kind of makes me feel like I belong because I know that they’ve also been through something similar to me. Because I can help talk to them and they can talk to me and we can be there for each other.”*  *“When I think about my experience about just being able to connect with other people in similar experiences and the impact that it made for me, I think it’s important because it’s really easy to feel isolated and alone in your experience. It’s really easy to easy to become disconnected from the world around you, and to lose that connection. I think for a long time I strived to want to feel normal, and in retrospect I think it was more that I didn’t want to feel abnormal. I think that being able to connect with other people that had that similar experience and that had that ability to say “yeah I know what that’s like” really helped me to not feel abnormal.”*  *“I was trying to carry on a conversation, and communicate in a constructive manner to keep my mind together on something other than the four brick walls staring at me with these mental cases walking around spacing my mind out.”*  *“There’s nothing to do on this ward, except watch television. Can’t talk to people about things. Some of the people don’t have brains to see past the television set. I don’t enjoy being with sick people. . . . I have a hard time communicating with most of the people of this ward . . . at certain times there were people who were in the hospital that I didn’t like very much, so I tried to avoid them. They were noisy, talked a lot, troublesome. . . . Lot of people around here are pretty weird, go off unexpectedly. I try to keep pretty quiet and stuff.”*  *“Some of the people here on the unit aren’t real pleasant to be around. It’s not uncomfortable; they’re just obnoxious people. They always mooch cigarettes and coffee from me, that’s about all they want. Some of them have been at the hospital 10 years as an inpatient and they don’t have anything to say because they never worked and they don’t have any kind of hobbies like myself. It’s kind of a sad situation, pretty depressing, those kind of people. In the hospital, there’s some pretty sick people and I don’t relate with them very well.”*  *“It’s been helpful to relate to someone really. Know that others have been through the same stuff that I went through but dealt with it better. Had different ways of coping… it’s made me realize that life is more precious really.”*  *“Thanks to the locale, I was saved. I could go there and meet other patients ... And when I met others who’d been in the hospital too, we started laughing, ‘‘Well, you were there too and now you’re here!’’ So we could laugh and joke and didn’t have to take it so seriously ... So that saved me .. . because my family sure can’t ... I’ve been very lonely.”*  *“First, there was this big ‘‘Aha-experience!’’ for me to hear all the other patients’ thoughts, because I then realized, of course, that some thoughts are actually disease-related, so I didn’t need to be ashamed of them, because everyone as crazy as me thinks this weirdly (laughs). It was very nice to have that confirmed in a way /... / and to then get an explanation for what is happening physically in the brain when things get crazy like this.”*  *“Every time you meet a new person, you meet a new universe ... so it’s good to meet the same person ... Otherwise, it can get kind of weird, and you don’t want that. You want security, someone who asks how you feel, and some help.”*  *“When I sit in the waiting room here in the psychosis clinic, I’m not just an anonymous figure in a sofa, but they greet me /.../ It is so important to know that they can see who I am as a person behind the disease.”*  *‘I don’t have any tips and pointers on how to stop feeling suicidal, other than to talk to people… I know, it might upset people but it’s just, when you are having feelings of suicide, and you feel like ending it all, it’s just, it can be a very lonely place.’*  *‘…a problem shared, is a problem halved. It shows you are not in a boat by yourself sorta thing, so… You’re not feeling this and maybe talking to someone else can help you.’*  *‘…the more I talked to people, the more I realised I weren’t on my own in this bullshit; it made me feel safer and better.’*  *‘It [talking] helps a lot. It’s good to mix with people and that… It gives you confidence.’*  *“We both have* schizophrenia, and we can talk about it. Well, he’s married with a whole family and I mean, you know, he’s got a family and kids and all that.”  *“I think it was good at the time (when psychosis worse) to be amongst others that experience the same thing and you know realise I’m not alone erm however some of the stories that I hear in there (HVN meetings) I just, you know, I don’t know, I haven’t felt any benefit from keeping going. I haven’t been going as often as I did Erm but erm yeah I know that from that group I know that people are in that state permanently and that’s lots of sad stories.”*  *“And I just want to be with these guys, and like in a group you know we can beat our psychosis. We can beat it and we can deal with it.”*  *“I’ve, sort of, moved on–I don’t want to be around people that remind me of what happened.”*  *“Coming here is a reminder of what happened before, right? Psychosis and that. Coming here for the social thing reminds me of all the bad stuff that’s happened.”* | Ogden, 2014  Andersson et al., 2015  Barut et al., 2016  Blajeski, 2022  DeNiro, 1995  Gajwani et al., 2016  Gunnmo & Bergman, 2011  Harris et al., 2019  Williams & Collins, 2002  Hogg et al., 2022  Macdonald et al., 2005  Huckle et al., 2021 |
| **Sub-theme 6.2:**  Substitutes for social connection | 1. Solitude versus proximity to others (inductive):  - Several participants indicated that proximity to others may help alleviate lonely feelings.  1. Recreation and work as protective activities (deductive):  - In contrast, participants described having a place to go away from home as helpful at alleviating loneliness and providing a sense of purpose. - Participants described running errands, completing work-related activities, or engaging in recreation to protect against or diminish loneliness severity.  1. What relieves the informants’ feeling of loneliness?:  - In a way, the informants had turned their backs on human relationships. - However, they had not given up their interest in creative and cultural activities. - The informants’ interests in art and their activities within different forms of the arts, such as painting, poetry and music etc., can be understood as the positive form of loneliness (solitude), where the different forms of art can increase the self-image and liberate positive forces.  1. Narratives:  - Glenda’s need for solitude was so great that sometimes cigarettes created an adequate replacement for socializing. - Despite her interpersonal connections, she could not feel completely at ease in the company of others, which could make her feel “exposed,” contributing to her need for solitude.  1. Additional Influences on Alienation:  - 60% described solitary activities that might help them feel less lonely, such as walking and listening to the radio.  1. Loneliness:  - Feelings of loneliness were denied by 3 patients who depended on hallucinatory voices for companionship.  1. Supportive content:  - This theme encompasses narratives about symptoms with a positive content. Generally, none of the participants described these voices as disturbing. The voices were mainly referred to as substitutes for loneliness and longing. - Positive voices were also reported with longing for a real person. Eve told of a voice belonging to a boyfriend who had broken up with her. Shortly after the breakup, she started to hear his voice. - Moreover, none of the participants expressed wishes to be rid of these symptoms.  1. Friendships facilitating the ability to cope:  - Some participants utilized their positive voices as a replacement for social relationships. These voices were used as a sounding board when making big decisions, were invited for companionship and helped participants cope with the impact of negative voices. - Participants with more depleted social networks appeared to place greater signiﬁcance on their voice relationships than participants with more social support. | *“[Loneliness] is more intense when I am physically alone… even more so at night because I am not around people and unable to reach out to people. Sometimes I’ll go to church or read in a coffee shop just so that people are there. Being around people definitely helps.”*  *“Sometimes I got so angry at my work, but then I analyzed what resulted in pleasure, and it was the drawing which gave me most joy and happiness.”*  *“I have [smoking] like it’s my second friend. That’s my friend . . . Keep me company when I am all alone...and I do like being alone you know? I do. Sometimes I like to be alone...It just relax my mind, just get away from everything, don’t think about nothing... People, you be too exposed to them, you shy away from them—you want to go home. Because there’s so much in people all the time.”*  *“Oh, I think a lot of the hallucinations fill that, always there, just a state of reality.”*  *“Not lonely because of the voices. My mother talks in my head.”*  *“The voice talks to me all day.”*  *“There was a part of me who really wanted to believe in this, that it was true, so I didn’t renew my prescription because I was feeling so bad, because at the same time this was my only link to him.”* | Ludwig et al., 2022  Ogden, 2014  DeNiro, 1995  Strand et al., 2015  Mawson et al., 2011 |
