# Supplementary material for: The experience of loneliness among people with psychosis: Qualitative meta-synthesis
Source: PLoS One. 2024 Dec 31;19(12):e0315763. doi: 10.1371/journal.pone.0315763 (PMC11687762; doi:10.1371/journal.pone.0315763)
Supplement: Attachment — Submitted filename: Review Plos 1.docx [file pone.0315763.s008.docx]

Review Plos 1

| Page | Section | Comment |
| --- | --- | --- |
| Overall |  | Thank you for this well executed, highly relevant paper. I support its publication. Below are some suggestions.  The paper is organised around two research questions: the experience of loneliness, contributory factors to loneliness, in psychosis. However, the Data Analysis and Discussion sections do not directly address these questions. The paper would be stronger if these were directly addressed.  Having meta-synthesised the themes how to these address the RQ’s. This might necessitate a more inductive/deductive analysis of the meta-synthesised themes.  To my mind there are two missed opportunities- one to propose a model of loneliness in psychosis- which is a fundamentally different experience that that of loneliness in the general population, which captures all the paradoxes inherent in psychosis and loneliness- which allows for the complexities in designing anti-loneliness interventions for this group. This is well captured in reference 44- “this study suggests nuance to the application of the Social Cure for a highly stigmatised, yet fluctuating, condition such as psychosis.”  two to create a visualisation which will capture this experience powerfully.  It would be interesting to address the RQ’s visually. How do the codes and subthemes cohere around the RQ’s. Figure 2 and S4 for instance, while accurate do not really address this. |
|  | Abstract | This seems clear and well described. The methods subsection can include whether PRISMA guidelines were followed, what the quality appraisal tool was. This guideline may be helpful [here](https://www.ncbi.nlm.nih.gov/pmc/articles/PMC3621753/) |
|  | Competing interests | The authors are funded by a loneliness charity. This is important. |
|  | Data Availability | Where are the data available?. For instance, if a researcher wished to re-analyse the data is the extracted data available? |
|  | Introduction | The introduction is clear and well written. It gives a good overview of the loneliness literature. However, it could be more specific. Considerable research has been done in psychosis/SMIs and loneliness, the specificity of this experience, and the challenges in addressing it. The introduction would be considerably stronger if it focused specifically on psychosis/loneliness. The introduction could really start on Pg4 Line 74 “Two Systematic reviews…”  The authors describe the challenge well “The  complexity of relationships between loneliness and psychosis suggests that approaches to  addressing this need to draw on an understanding of the distinctive features of experiences of  loneliness among people with psychosis”. However, in my opinion they could spend more time identifying these distinctive features in the Introduction |
|  |  | Reference 25- In one line, and one reference, the authors discuss loneliness interventions and psychosis. This is a substantial area of literature with considerable evidence already gathered, which could be further explored, especially the challenges inherent in the area- how do support someone to socialise who experiences paranoia, social anxiety, anhedonia or social withdrawal.  Many, many researchers are interested in reducing loneliness in psychosis. The question, which this review is aiming to address, is how? |
|  | Methodology | This section is well described |
|  | Thematic Synthesis | Although this is a perfectly reasonable approach to use, I wonder whether it would be supported by a more deductive approach, namely structuring the codes around the two RQs: the experience of loneliness, contributory factors loneliness in psychosis. This would necessitate two analyses rather than one. I’m loathe to suggest the authors re-analyse the data but then a better way of addressing the RQs in the Results and Discussion. Which themes/subthemes best address each question? Can this be done graphically? |
|  | Results | These are clearly described |
|  | Discussion | This is clearly described but, as noted above, the RQs aren’t addressed directly. For instance, based on these data what should a direct anti-loneliness intervention comprise? The authors cite Ref 24, but we’ve already established that the evidence base for this is poor.  There is room here to look at Social Identity Theory, or other social theories of belonging.  Indirect, public health interventions are important but often have paradoxical outcomes of increasing stigma. For instance. <https://doi.org/10.3109/09638237.2015.1057327>  Similarly, the Clinical Implications section notes some relatively generic advice, not specifically based on the data on this paper, and not addressing the challenges highlighted by Ref 24, 25, 44 |
|  | Future Research | This is a little generic, and does not seem particularly informed by the data in the study. Specifically, what does the data suggest is the next step in psychosis/loneliness research.  There is an established evidence base for self-esteem interventions.  “Further qualitative research with a specific focus on the nature of loneliness and sense of belonging, and the influences on these, is needed both among individuals with a psychotic diagnosis and those with a high risk of psychosis.” That’s this study.  There is an added level of depth which could be drawn from the data to address the “nature of loneliness” and “the influence on these” |
|  |  |  |
|  |  |  |
|  |  |  |
|  |  |  |
|  |  |  |
|  |  |  |
|  |  |  |
|  |  |  |
|  |  |  |
|  |  |  |
|  |  |  |
|  |  |  |
|  |  |  |
|  |  |  |
|  |  |  |
|  |  |  |
|  |  |  |
|  |  |  |
|  |  |  |
|  |  |  |
|  |  |  |
|  |  |  |
|  |  |  |
|  |  |  |
|  |  |  |
|  |  |  |
|  |  |  |
|  |  |  |
