# Supplementary material for: The experience of loneliness among people with psychosis: Qualitative meta-synthesis
Source: PLoS One. 2024 Dec 31;19(12):e0315763. doi: 10.1371/journal.pone.0315763 (PMC11687762; doi:10.1371/journal.pone.0315763)
Supplement: Attachment — Submitted filename: Response to Reviewers.docx [file pone.0315763.s009.docx]

**Manuscript reference number: PONE-D-24-33999**

Re: Response to Reviewers

Dear Editor,

Thank you for the opportunity to submit a revised draft of the manuscript *“The experience of loneliness among people with psychosis: qualitative meta-synthesis”.* We appreciate the time and effort the editors and peer reviewers dedicated to providing feedback on our manuscript. We have considered and addressed comments, and highlighted revisions within the manuscript using tracked changes. Please also see below for a point-by-point response to the editors’ and reviewers’ comments. All page numbers refer to the revised manuscript file with tracked changes.

**Journal Requirements:**

**Point 1:** Please ensure that your manuscript meets PLOS ONE's style requirements, including those for file naming.

**Author response:**

Thank you. We have reviewed the PLOS ONE style requirements, including those for file naming, and made the necessary changes.

**Point 2:** We note that the grant information you provided in the ‘Funding Information’ and ‘Financial Disclosure’ sections do not match. When you resubmit, please ensure that you provide the correct grant numbers for the awards you received for your study in the ‘Funding Information’ section.

**Author response:**

Thank you. We have provided the correct grant numbers (grant reference: ES/S004440/1).

**Point 3:** Thank you for stating the following financial disclosure: “SJ, AP and BLE receive salary support from the Loneliness & Social Isolation in Mental Health Research Network, which was funded by UK Research and Innovation (Grant reference: ES/S004440/1) and is now funded by the NIHR UCLH BRC. Their support is gratefully acknowledged.” Please state what role the funders took in the study. If the funders had no role, please state: "The funders had no role in study design, data collection and analysis, decision to publish, or preparation of the manuscript.". If this statement is not correct you must amend it as needed. Please include this amended Role of Funder statement in your cover letter; we will change the online submission form on your behalf.

**Author response:**

Thank you for this feedback, we have slightly revised the funding statement and included the amended Role of Funder statement (see cover letter).

**Point 4**: Please include captions for your Supporting Information files at the end of your manuscript, and update any in-text citations to match accordingly.

**Author response:**

We have included captions for our Supporting Information files at the end of the manuscript, and updated any in-text citations to match accordingly.

**Point 5:** As required by our policy on Data Availability, please ensure your manuscript or supplementary information includes the following (see email for full list, we have responded to those that are applicable to our review):

**Author response:**

All relevant data has been included in the manuscript and its supporting files. We have created a table of all studies excluded at full text with the reason for exclusion and included this in Appendix S4**.** The list of eligible articles and extracted data including study characteristics can be found in Appendix S5, the assessment of study quality according to the Critical Appraisal Skill Programme (CASP) is in Appendix S6. The themes, sub-themes and illustrative quotations by both the original authors and participants contributing to the meta-synthesized themes are reported in the results section and also recorded in Appendix S7.

**Reviewers' Comments to the Authors:**

**Reviewer 1**

**Point 1:** Thank you for this well executed, highly relevant paper. I support its publication.

**Author response**:

Thank you for your positive comments regarding our paper.

**Point 2:** The paper is organised around two research questions: the experience of loneliness, contributory factors to loneliness, in psychosis. However, the Data Analysis and Discussion sections do not directly address these questions. The paper would be stronger if these were directly addressed. Having meta-synthesised the themes how do these address the RQ’s. This might necessitate a more inductive/deductive analysis of the meta-synthesised themes.

**Author response**:

Thank you for your feedback. We agree that it is important for us to directly address the research questions set out under our aims. We have set this approach out more clearly in our methods (page 10, line 247-250), and also summarised in our discussion the main findings with reference to our original research questions. This therefore makes a clearer connection between our two research questions and our six themes – see page 34 (line 1203-1222):

“Our analysis identified two themes that mapped to our first research question about how individuals with psychosis experienced loneliness. We found that people with psychosis experienced loneliness in the form of disconnection from self and the others (theme 1), and they described a paradoxical yearning for closeness and distance, driven by fear, anxiety and self-stigma (theme 5). Our second research question related to identifying the factors people with psychosis perceive as contributing to the development and persistence of loneliness, and we identified three themes that addressed this. Individuals with psychosis consistently highlighted their experiences of rejection and stigmatisation as the source of loneliness, particularly where there was early contact with psychiatric services, which could aggravate loneliness (theme 2). Some also described acute psychotic episodes and periods of mental health crisis as constituting the most intense periods of loneliness in their lives (theme 3). Participants also shared the various psychological and structural barriers that could hinder social contact, including social anxiety, insecurity and the label of low socioeconomic status associated with psychosis (theme 4). Our analysis identified one theme (theme 6) outside the scope of our research questions: how social connections, when trusting and reciprocal, could build belongingness and reduce the feelings of loneliness. This theme influenced all other themes in our analysis by operating as a mitigating factor (i.e., influencing the onset and/or perpetuation of loneliness in people with psychosis), which was highly relevant to the exploration of the experience and perpetuating factors of loneliness among people with psychosis.”

**Point 3:** To my mind there are two missed opportunities:

1. to propose a model of loneliness in psychosis- which is a fundamentally different experience than that of loneliness in the general population, which captures all the paradoxes inherent in psychosis and loneliness- which allows for the complexities in designing anti-loneliness interventions for this group. This is well captured in reference 44- “this study suggests nuance to the application of the Social Cure for a highly stigmatised, yet fluctuating, condition such as psychosis”.
2. to create a visualisation which will capture this experience powerfully. It would be interesting to address the RQ’s visually. How do the codes and subthemes cohere around the RQ’s. Figure 2 and S4 for instance, while accurate do not really address this.

**Author response**:

Thank you for these valuable suggestions. Regarding the first point, our current paper aims to “provide a synthesis of such evidence in order to inform further work to understand the relationship between loneliness and psychosis, as well as the potential directions for intervention development,” We acknowledge the value in proposing a conceptual model of loneliness in psychosis, however the literature is not sufficiently developed for a clear conceptual model. We hope that with our meta-synthesis, we can summarise existing qualitative research on the experience of loneliness to underpin further conceptual and empirical work on the topic. We have made clearer in our discussion the gaps of understanding needed to be addressed before a conceptual model can be developed – see page 35 (line 1241-1246):

“We did not find any qualitative accounts that described how the experience of loneliness might vary with respect to the different types and phases of psychotic disorders, the content and nature of hallucinations or delusions, the balance of positive versus negative symptoms, and the specific patient characteristics (e.g. ethnicity, socioeconomic status, sexuality, and any other characteristics of interest). These are evidence gaps for future qualitative exploration.”

We have suggested the development of conceptual model under the section on clinical implications and future research (page 41, line 1428-1436):

“Given the evidence gaps we identified, there is a need for empirical studies to explore how the experience of loneliness varies with respect to factors such as stages of illness, types of psychotic disorders, or specific patient characteristics. Future qualitative interview studies should use purposive sampling to ensure that people from a range of backgrounds, stages of recovery and marginalised groups are included. This would allow us to gain an in-depth understanding of loneliness and how it is influenced by societal and social barriers. Once these phenomenological knowledge gaps are addressed, it will be appropriate to develop a conceptual model that captures the complexity of the relationships between loneliness and psychosis.”

Regarding the second point, we have now mapped the themes to the research questions and replaced the original Fig 2 with a pictorial representation.

**Point 4: Abstract:** This seems clear and well described. The methods subsection can include whether PRISMA guidelines were followed, what the quality appraisal tool was. This guideline may be helpful [here](https://www.ncbi.nlm.nih.gov/pmc/articles/PMC3621753/)

**Author response**:

Thank you. We have added the information about PRISMA guidelines and quality appraisal tool to the methods subsection (page 2, line 30-33).

**Point 5: Data availability:** Where are the data available? For instance, if a researcher wished to re-analyse the data is the extracted data available?

**Author response**:

Thank you for this comment. Our study represents a secondary analysis of the findings presented in published research papers, which we extracted based on relevant text in each article that was relevant to the study research questions (direct quotations and authors’ interpretations). All relevant data has been included in the manuscript and its supporting files. The list of eligible articles and their characteristics can be found in Appendix S5. The themes, sub-themes and illustrative quotations by both the original authors and participants contributing to the meta-synthesized themes are recorded in Appendix S7.

**Point 6:** The introduction is clear and well written. It gives a good overview of the loneliness literature.

However, it could be more specific. Considerable research has been done in psychosis/SMIs and loneliness, the specificity of this experience, and the challenges in addressing it.

The introduction would be considerably stronger if it focused specifically on psychosis/loneliness. The introduction could really start on Pg4 Line 74 “Two Systematic reviews…”

The authors describe the challenge well “The complexity of relationships between loneliness and psychosis suggests that approaches to addressing this need to draw on an understanding of the distinctive features of experiences of loneliness among people with psychosis”. However, in my opinion they could spend more time identifying these distinctive features in the Introduction

**Author response**:

Thank you for the constructive feedback. We have removed the lines about the prevalence of loneliness in the general population from the manuscript to make the introduction more concise. We acknowledge that loneliness is a growing topic within research on common mental disorder as well as severe mental illness, particularly the former. By providing a general overview of the literature on loneliness at the start of our introduction, we allowed readers who might not be familiar with the concept of loneliness to gain a clearer understanding about its definition and its relationships with different mental health conditions.

**Point 7:** Reference 25- In one line, and one reference, the authors discuss loneliness interventions and psychosis. This is a substantial area of literature with considerable evidence already gathered, which could be further explored, especially the challenges inherent in the area- how do support someone to socialise who experiences paranoia, social anxiety, anhedonia or social withdrawal.

Many, many researchers are interested in reducing loneliness in psychosis. The question, which this review is aiming to address, is how?

**Author response**:

Thank you for the comment. We acknowledge the importance of qualitative evidence for the development of direct loneliness interventions to support individuals with psychosis. As the first qualitative meta-synthesis of studies exploring the experience of loneliness in psychosis, we aimed to provide a synthesis of the existing evidence to form a foundation for informing further research and intervention development. We have made clearer links between our findings and intervention approaches – see Point 11 (page 37, line 1306-1322). We have also added specific clinical implications informed by the data of this study – see Point 14 (page 40, line 1401-1414).

**Point 8:** Methodology section is well described

**Author response**:

Thank you for the positive appraisal.

**Point 9:** Although this is a perfectly reasonable approach to use, I wonder whether it would be supported by a more deductive approach, namely structuring the codes around the two RQs: the experience of loneliness, contributory factors loneliness in psychosis. This would necessitate two analyses rather than one. I’m loathe to suggest the authors re-analyse the data but then a better way of addressing the RQs in the Results and Discussion. Which themes/subthemes best address each question? Can this be done graphically?

**Author response**:

Thank you for the comment. We have addressed this under Point 2, by making a clearer connection between our two research questions and our six themes in the discussion. We have also created a pictorial representation of the themes and research questions to replace our original Fig 2.

**Point 10:** Results are clearly described

**Author response**:

Thank you for the positive comment.

**Point 11: Discussion:** This is clearly described but, as noted above, the RQs aren’t addressed directly. For instance, based on these data what should a direct anti-loneliness intervention comprise? The authors cite Ref 24, but we’ve already established that the evidence base for this is poor.

**Author response**:

Thank you for this suggestion. Our analysis suggested that some approaches in Mann et al., 2017 may work for some people with psychosis, but further research in targeted approaches for specific sub-groups of individuals with psychosis is likely to be the most promising way in building up a set of interventions for different people with psychosis. We have added the following to make clearer links between our findings and intervention approaches – see page 37 (line 1306-1322):

“Our findings regarding the psychological and structural barriers to social engagement suggest that some direct and indirect approaches in loneliness interventions, as identified in two scoping and systematic reviews on the interventions for subjective social isolation in people with mental health problems (21,22), may be beneficial for individuals with psychosis depending on their needs. For example, the chronic nature of loneliness and its close links to symptoms and cognitions among people with psychosis suggest a need to consider direct approaches focused on social skills training and challenging unhelpful cognitions (e.g., lack of interpersonal trust), which is in keeping with previous studies on the current challenges and gaps in loneliness interventions among people with psychosis (10,12). These intervention approaches can benefit from offering people with psychosis the support in connecting with peers who have similar experiences, and improving the quality of their pre-existing relationships with friends and family. In addition, our findings on the links between stigma and loneliness highlight the importance of tackling social challenges presented by low socioeconomic status associated with psychosis (e.g., improving education and employment opportunities), as well as the significance of community-wide initiatives in addressing stigma of psychosis and facilitating integration into the wider community.”

**Point 12: Discussion:** There is room here to look at Social Identity Theory, or other social theories of belonging.

**Author response**:

Thank you for the comment. We agree that social identify theory offers important insights to the topic. We have added the following to discussion on pages 36-37 (line 1289-1306):

“These data highlight the integral role of social connection in integrating individuals with the community and bringing about a sense of normality and purpose in life, as well as the importance of having agency over group memberships. These findings align with the social identity theory, which postulates that social identification may reduce the symptoms of paranoia by increasing perceived control, trust and self-esteem (43,94,95).”

**Point 13: Discussion:** Indirect, public health interventions are important but often have paradoxical outcomes of increasing stigma. For instance. <https://doi.org/10.3109/09638237.2015.1057327>

**Author response**:

Thank you for raising this point and highlighting the research findings in O'Keeffe et al., 2016. We agree that the findings of this paper are important to mention, and have added the following text to our discussion on page 37 (line 1322-1326):

“It is important to note that educational campaigns focused on improving public mental health literacy might not reduce stigma if they promote the idea of “otherness” in relation to people with psychosis, therefore anti-stigma interventions could benefit from a focus on optimism about recovery and challenging the perceived dangerousness of psychosis (96).”

**Point 14: Discussion:** Similarly, the Clinical Implications section notes some relatively generic advice, not specifically based on the data on this paper, and not addressing the challenges highlighted by Ref 24, 25, 44.

**Author response**:

Thank you for the comment. Based on our evidence, we found that addressing maladaptive cognitions, challenging stigma, and facilitating social connections were important in alleviating loneliness among people with psychosis. We have made these pointers clearer when addressing Points 11, 12 and 13, and have added the following to the section on clinical implications – see page 40 (line 1401-1414):

“In addition, the centrality of a sense of belongingness in alleviating loneliness suggests a need to develop socially-focused interventions that connect individuals with communities that they hope to develop social group membership and sense of self in. Signposting to peer support from people with similar lived experience and social prescribing are potential approaches in linking individuals to social and emotional support, thus bringing about a sense of purpose and normality through social connections. Loneliness intervention development would benefit from community-wide campaigns that focus on addressing the marginalisation of people with psychosis, and reducing the stigma associated with psychosis. Our findings also highlight the potential importance of challenging maladaptive social cognitions in alleviating loneliness, through social skills training and challenging internalized stigma associated with psychosis. In conjunction with the evidence of high self-esteem mediating the link between social identification and reduced psychotic symptoms, such as paranoia, avolition and social withdrawal (98,99), cognitive behavioural treatment for self-esteem could be beneficial for people with psychosis.”

**Point 15: Future research:** This is a little generic, and does not seem particularly informed by the data in the study. Specifically, what does the data suggest is the next step in psychosis/loneliness research.

There is an established evidence base for self-esteem interventions.

“Further qualitative research with a specific focus on the nature of loneliness and sense of belonging, and the influences on these, is needed both among individuals with a psychotic diagnosis and those with a high risk of psychosis.” That’s this study.

There is an added level of depth which could be drawn from the data to address the “nature of loneliness” and “the influence on these”

**Author response**:

Thank you for your comment. We have added the following to offer suggestions informed by the data of the study – see pages 40-41 (line 1416-1436):

“Future studies are required to develop co-produced interventions for psychosis whose feasibility, acceptability and effectiveness can then be tested. Only four included articles explicitly aimed to investigate the experience of loneliness among individuals with psychosis, therefore further qualitative research with a specific focus on the nature of loneliness and sense of belonging, and the influences on these, is needed both among individuals with a psychotic diagnosis and those with a high risk of psychosis. It is also important for future research to probe the roles of social cognitions and stigma in the context of loneliness and psychosis. Such data can deepen our understanding of the various pathways linking loneliness and psychotic symptoms. Given the evidence gaps we identified, there is a need for empirical studies to explore how the experience of loneliness varies with respect to factors such as stages of illness, types of psychotic disorders, or specific patient characteristics. Future qualitative interview studies should use purposive sampling to ensure that people from a range of backgrounds, stages of recovery and marginalised groups are included. This would allow us to gain an in-depth understanding of loneliness and how it is influenced by societal and social barriers. Once these phenomenological knowledge gaps are addressed, it will be appropriate to develop a conceptual model that captures the complexity of the relationships between loneliness and psychosis.”

**Reviewer 2**

**Point 1:** Thank you for inviting me to review this very insightful review. This is topic requiring further research and development of co-designed interventions, so I think this review provides a good platform for that work.

**Author response**:

Thank you for the positive appraisal of our submission.

**Point 2:** Much of my feedback is methodological, in the spirit of demonstrating the quality of the review process. I am conscious that you refer to meta-synthesis but I would suggest qualitative evidence synthesis (QES) as the preferred term, within which thematic synthesis is one of the more common approaches. So, if I refer to QES you can apply that to “meta-synthesis”

**Author response**:

Thank you for the suggestion. As mentioned in our methods section, meta-synthesis follows a six-step process adapted from thematic synthesis, as described in Lachal et al., 2017. We acknowledge that QES is an umbrella term for a number of approaches to qualitative synthesis (<https://journals.sagepub.com/doi/full/10.1177/1609406921993276>), therefore we think keeping the terminology “meta-synthesis” allows us to be clear and specific about the approach we had taken in synthesising our data. We also used the term “meta-synthesis” in previous studies, such as: [Achterbergh et al., 2020](https://bmcpsychiatry.biomedcentral.com/articles/10.1186/s12888-020-02818-3); [Ikhtabi et al., 2022](https://pmc.ncbi.nlm.nih.gov/articles/PMC8855579/); [Adlington et al., 2023](https://pmc.ncbi.nlm.nih.gov/articles/PMC9970854/).

**Point 3:** First and foremost, I would urge the authors to consider ENTREQ reporting guideline (<https://www.ncbi.nlm.nih.gov/pmc/articles/PMC3552766/>) available through the EQUATOR network. The EPOC template for reporting Qualitative Evidence Synthesis (QES): <https://zenodo.org/records/10050961> Is also very useful for preparing your review report.

**Author response**:

Thank you for your valuable suggestion. We have reviewed our manuscript and ensured that our reporting adheres to the ENTREQ guidelines. To address this, we have included an ENTREQ checklist in Appendix S2, specifying the location of each reporting item within our manuscript. Additionally, we have added a sentence in the methods section (page 6, line 146-147) to indicate that:

"The reporting of this meta-synthesis adhered to the ENTREQ guideline (28) (see Appendix S2)."

**Point 4:** Using more appropriate reporting guidance would help to address some of the omissions I identified in the paper, for instance, why thematic synthesis? (as opposed to framework synthesis or meta-ethnography).

**Author response**: Thank you. We have provided an explanation for the use of thematic synthesis in the data analysis section of the methods. Please see pages 10-11 (line 255-268):

“Thematic synthesis was employed to analyse the relevant findings from the studies included in this review (34). This approach was selected due to its flexibility in synthesising findings from diverse qualitative studies (31,34) including the subjective and multifaceted experiences of loneliness, which can vary across individuals and contexts. Unlike framework synthesis, thematic synthesis allows themes to be developed inductively and without the constraints of a pre-existing framework, thus enabling the richness and depth of participants' experiences to be captured (34). Similarly, meta-ethnography is often more focused on translating and synthesising studies into new theoretical models, which is less suited to the aim of this review.”

**Point 5:** You have used literature to support your methodology (refs 31-35) but some of these are dated and there has been huge methodological advancements since. I would strongly urge you to review more contemporary literature in this area to inform your review stages.

**Author response**:

Thank you for the comment. One of the references was published in 2017 (ref 31). We have reviewed more contemporary literature and replaced ref 32 and 33 with the following literature:

Flemming K, Noyes J. Qualitative Evidence Synthesis: Where Are We at? International Journal of Qualitative Methods. 2021;20.

Leary H, Walker A. Meta-Analysis and Meta-Synthesis Methodologies: Rigorously Piecing Together Research. TechTrends. 2018;62:525–534.

**Point 6:** In terms of quality appraisal, CASP is admittedly quite difficult to use but there would be a strong feeling in the community that the studies should not be scored as it remains quite subjective. Usually, a “yes” “no” or “unclear/can’t tell” format is used.

**Author response**:

Thank you for the comment. We chose the CASP because it is the most commonly used tool for quality appraisal in health-related QES, with endorsement from the Cochrane Qualitative and the World Health Organisation (<https://pubmed.ncbi.nlm.nih.gov/29247700/>), and as evidenced by other published research (e.g., [Achterbergh et al., 2020](https://bmcpsychiatry.biomedcentral.com/articles/10.1186/s12888-020-02818-3); [Ikhtabi et al., 2022](https://pmc.ncbi.nlm.nih.gov/articles/PMC8855579/); [Adlington et al., 2023](https://pmc.ncbi.nlm.nih.gov/articles/PMC9970854/)) using the CASP scoring system. We aimed to be transparent about our methods through the reporting of quality of the included papers. Using the CASP scoring method allowed us to distinguish between higher and lower quality studies, and provided a clearer understanding of the overall quality of evidence included in the review. To reduce the risk of bias in the quality appraisal process, two reviewers of our team appraised the quality of each included study independently, and discrepancies were resolved through discussion until consensus reached.

**Point 7:** There is no reference to the confidence in your findings outside of the limitations of the primary studies. GRADE CERQual is commonly used now- I think it should be acknowledged as a limitation that there was no assessment of confidence, nor sensitivity analysis conducted for this review.

**Author response**: Thank you for this comment. We agree that this is an important limitation to highlight in our discussion. We have included the following on pages 38-39 (line 1365-1371):

“Another limitation of this review is that, due to resource and time constraints, a sensitivity analysis and an assessment of the certainty of evidence (e.g., the GRADE-CERQual approach) were not conducted for each individual finding. Therefore, the findings of this review should be interpreted with caution.”

**Point 8:** Theme development (please avoid term “emerge”): I am just wondering how subthemes 2.1 and 2.2 fit within the theme of being rejected by external world? Maybe it is just the phrasing, but it is not clear for me.

**Author response**: Thank you for the comment. We have reworded the term “emerge” in our paper.

Sub-themes 2.1 and 2.2 described how the experiences of rejection and stigmatisation across one’s life story and during early contact with psychiatric services could lead to the feelings of loneliness. We have rephrased the titles of the sub-themes to the following:

Sub-theme 2.1: Sense of exclusion across the life story

Sub-theme 2.2: Stigma and loneliness in early contact with psychiatric services

**Point 9:** With such a broad range of participants it would be helpful to know a bit more about participants when providing direct quotes, age in particular, when talking about friendships etc.

**Author response**:

Thank you for the feedback. We acknowledge that it would be helpful to know the characteristics of the participants when providing direct quotes, and we made efforts to be mindful of this by presenting the quotes with the relevant participants’ characteristics when the information was available. However, not all included research specified demographic characteristics when presenting the participants’ quotes. We included this limitation on page 39 (line 1374-1377):

“Some papers did not specify participants’ demographic characteristics or the types of psychotic disorders they had, which reduced our capacity to investigate patterns of qualitative themes by age, gender, ethnicity, and context of psychotic symptoms.”

**Point 10:** Page 33-34 of the discussion reads like a list of the themes and doesn’t add much to the discussion.

**Author response**:

Thank you for the comment. We have addressed a similar comment from Reviewer 1. To add to the discussion, we have made a clearer connection between our themes and the RQs on page 34 (line 1203-1222). We have also added more specific suggestions to our clinical implications on page 40 (line 1401-1414), including the centrality of belongingness in alleviating loneliness, and the importance of addressing stigma and unhelpful cognitions in loneliness interventions.

**Point 11:** I hope my feedback is helpful in enhancing the reporting of this very interesting review.

**Author response**:

Thank you for the constructive comments.

We would like to thank again to both reviewers for their review and comments.

Best Wishes,

The study’s author team
